# Friedel-Crafts Reaction of N,N-Dimethylaniline with Alkenes Catalyzed by Cyclic Diaminocarbene-Gold(I) Complex

**DOI:** 10.1038/s41598-018-29854-0

**Published:** 2018-07-30

**Authors:** Hangzhi Wu, Tianxiang Zhao, Xingbang Hu

**Affiliations:** 1 School of Chemistry and Chemical Engineering, Nanjing University, Nanjing, 210093 P. R. China; 2 High School Affiliated to Nanjing Normal University, Nanjing, P. R. China

## Abstract

In general, Friedel-Crafts reaction is incompatible with amines due to the Lewis acidity of the catalysts. Recently, we reported that cyclic diaminocarbene-Gold(I) can be used as catalyst for the Friedel-Crafts alkylation between aromatic amines and alkenes. Herein, a systematically theoretical research was performed on this rare Friedel-Crafts reaction. The adopted calculation method is accurate enough to reproduce the crystal structure of the catalyst. It was found that the reactions followed the electrophilic aromatic substitution mechanism. The gold cation can activate the C=C double bond and generate the electrophilic group which can be attacked by the aromatic ring. The *para*-product is more energy favorable which agrees well with the experimental results. The reaction of α-methylstyrene follows the Markovnikov rule, and the activation energy to generate the branched product of methylstyrene is lower than that producing the linear product. However, the reaction of butanone follows the anti-Markovnikov rule, and the activation energy to generate the branched product of butanone is higher than that producing the linear product. These calculation results reveal the mechanism of this new Friedel-Crafts reaction. It can well explain the high *para*-selectivity and the substrate-dependent of the product structures in the experiment.

## Introduction

Friedel-Crafts (FC) reaction is an important method to incorporate carbon skeletons into aromatic system^1,2^. Great successes have been achieved for the hydroarylation of neutral arenes (such as toluene, anisole, and their homologues)^3–14^. Because the FC reactions typically require Lewis acid catalysts, for arenes containing nitrogen atom, the substrate scope of FC reactions are quite limited due to the coordination between amine and Lewis acid catalyst, except indole and pyrrole^15–23^. Being profited from the extremely weak basic properties^24,25^, acid-catalyzed additions of indole and pyrrole to alkenes have obtained great achievements^15–23^. However, the hydroarylation of alkaline arenes to alkenes still remains many challenges. Some researches have shown the possibility of hydroarylation between the parent anilines C_6_H_5_NH_2_ and alkenes^26–28^. However, the reaction of arenes with stronger basicity (such as N,N-dimethylaniline and N,N-diethylaniline^29^) still is a big problem, due to their ability to coordinate with Lewis acid catalyst which can lead to deactivation of the aromatic ring^30^. Furthermore, alkaline arenes can trap the proton in the C-H activation process and the reaction will be terminated as result^26^.

**Figure 1:**
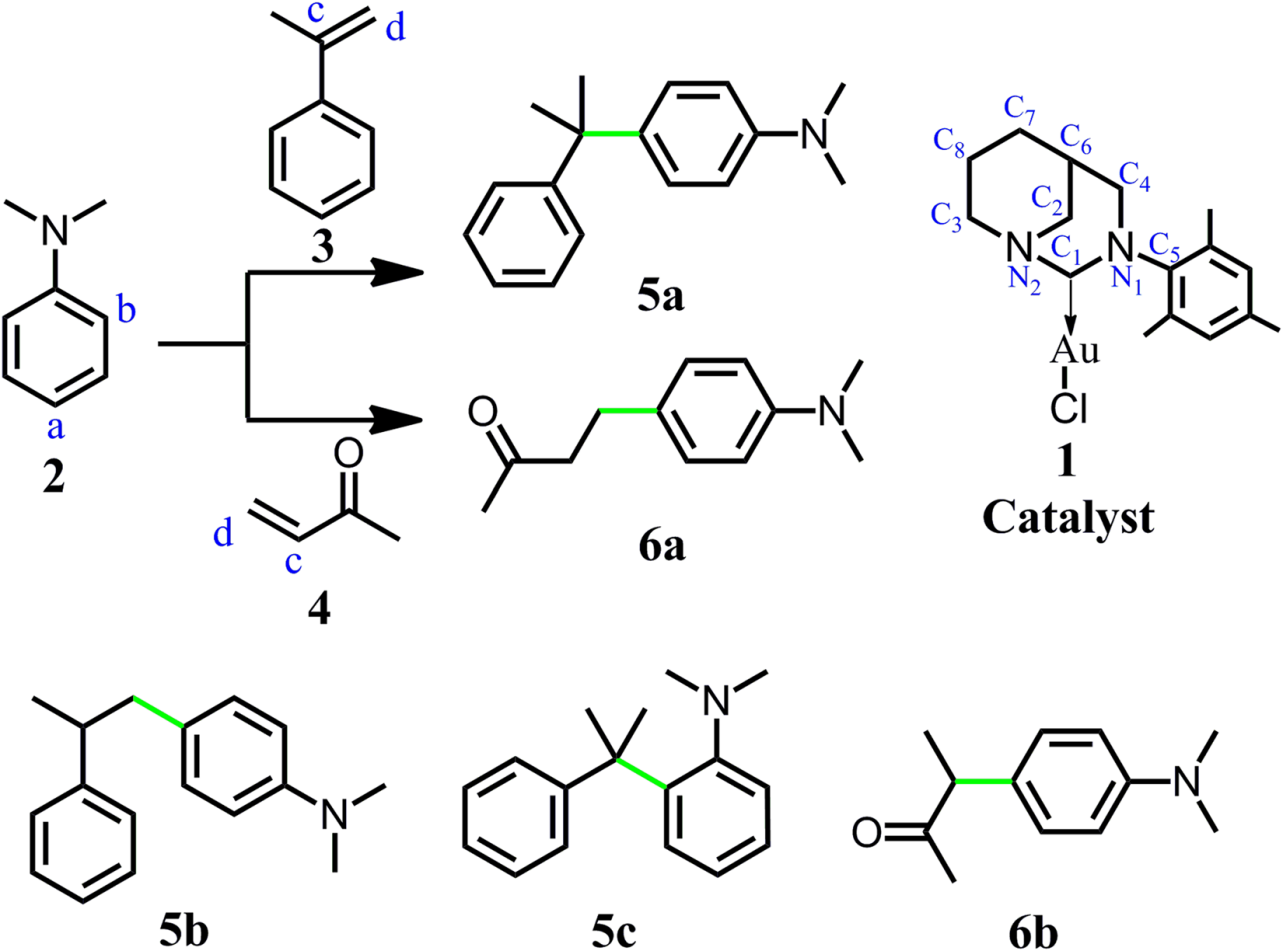
The Friedel-Crafts reactions of N,N-Dimethylaniline and corresponding products (**5a** and **6a** are the main products in the experiment^33^).

Recently, Bertrand *et al*. reported an anti-Bredt cyclic diaminocarbene which showed increased π-accepting character without diminishing its σ-donor property^31,32^. We found that Gold(I) compound derived from this new carbene can be used as effective catalyst for the FC reaction between alkenes and N,N-dialkylanilines^33^. Now, these new FC reactions are receiving more and more research interests^34–37^. As we known, most of the electrophilic substitution reactions followed the Markovnikov rule^38^. For the FC reaction of alkenes, the reactions following the Markovnikov rule should form branched product^3,4^. Only several examples were reported on the formation of linear product by anti-Markovnikov rule^39,40^. For the FC reactions between alkenes and N, N-dialkylanilines catalyzed by carbene Gold(I), both Markovnikov and anti-Markovnikov hydroarylations were observed and all these reactions gave high *para*-selectivity products (Fig. 1)^33^. The selectivity to the branched or linear product was highly depended on the structure of alkenes. The purpose of this paper is to find out the reaction mechanism of this rare FC reaction and understand the origin of the reaction selectivity.

## Results and Discussion

### The structure of the catalyst

To examine the reliability of the theoretical method, the structure of the catalyst **1** was fully optimized with both B3LYP and M06-2X methods. The calculated results were compared with the x-ray experiment data (Table 1). The calculated structure parameters match well with the experiment data. Most of the calculation errors for the bond length and angle are smaller than 1.0% and the maximum error is 1.7%. It suggests that the calculation method used here is accurate enough to reproduce the crystal structure even though the catalyst contain gold atom. Indeed, methods based on DFT using pseudopotential basis set have been widely used for the calculation of compound containing gold^41–51^. Our previous experimental results on the hydration of alkynes catalyzed by gold(I) isocyanide were successfully explained by theoretical calculation based on DFT^41^.

**Table 1:** Some important structure parameters of the carbene gold (I) catalyst **1** obtained by calculation and experiment^a^.

	Exp.^b^	Calc.	CE (%)^c^
C_1_-Au	1.987	2.013 [2.013]	1.3 [1.3]
C_1_-N_1_	1.329	1.352 [1.342]	1.7 [1.0]
C_1_-N_2_	1.375	1.382 [1.375]	0.5 [0.0]
C_2_-N_2_	1.470	1.481 [1.472]	0.7 [0.1]
C_3_-N_2_	1.488	1.487 [1.479]	−0.1 [−0.6]
C_4_-N_1_	1.491	1.506 [1.491]	1.0 [0.0]
C_5_-N_1_	1.449	1.455 [1.446]	0.4 [−0.2]
C_2_-C_6_	1.516	1.532 [1.528]	1.1 [0.8]
C_3_-C_8_	1.537	1.549 [1.543]	0.8 [0.4]
C_7_-C_8_	1.532	1.557 [1.551]	1.6 [1.2]
∠N_1_C_1_N_2_	116.5	115.4 [115.6]	−0.9 [−0.8]
∠C_1_N_2_C_2_	117.5	117.7 [117.6]	0.2 [0.1]
∠C_1_N_1_C_4_	123.7	123.3 [124.1]	−0.3 [0.3]
∠C_1_N_1_C_5_	120.9	121.3 [120.6]	0.3 [0.2]
∠N_2_C_2_C_6_	106.0	105.3 [105.4]	−0.7 [0.6]
∠C_2_C_6_C_4_	104.4	104.2 [104.2]	−0.2 [−0.2]

^a^Bond length in Å and angles in degree obtained by B3LYP [M06-2X] method; ^b^ref.^33^; ^c^CE: Calculation Error = (Calc. − Exp.)/Exp. *100%.

**Figure 2:**
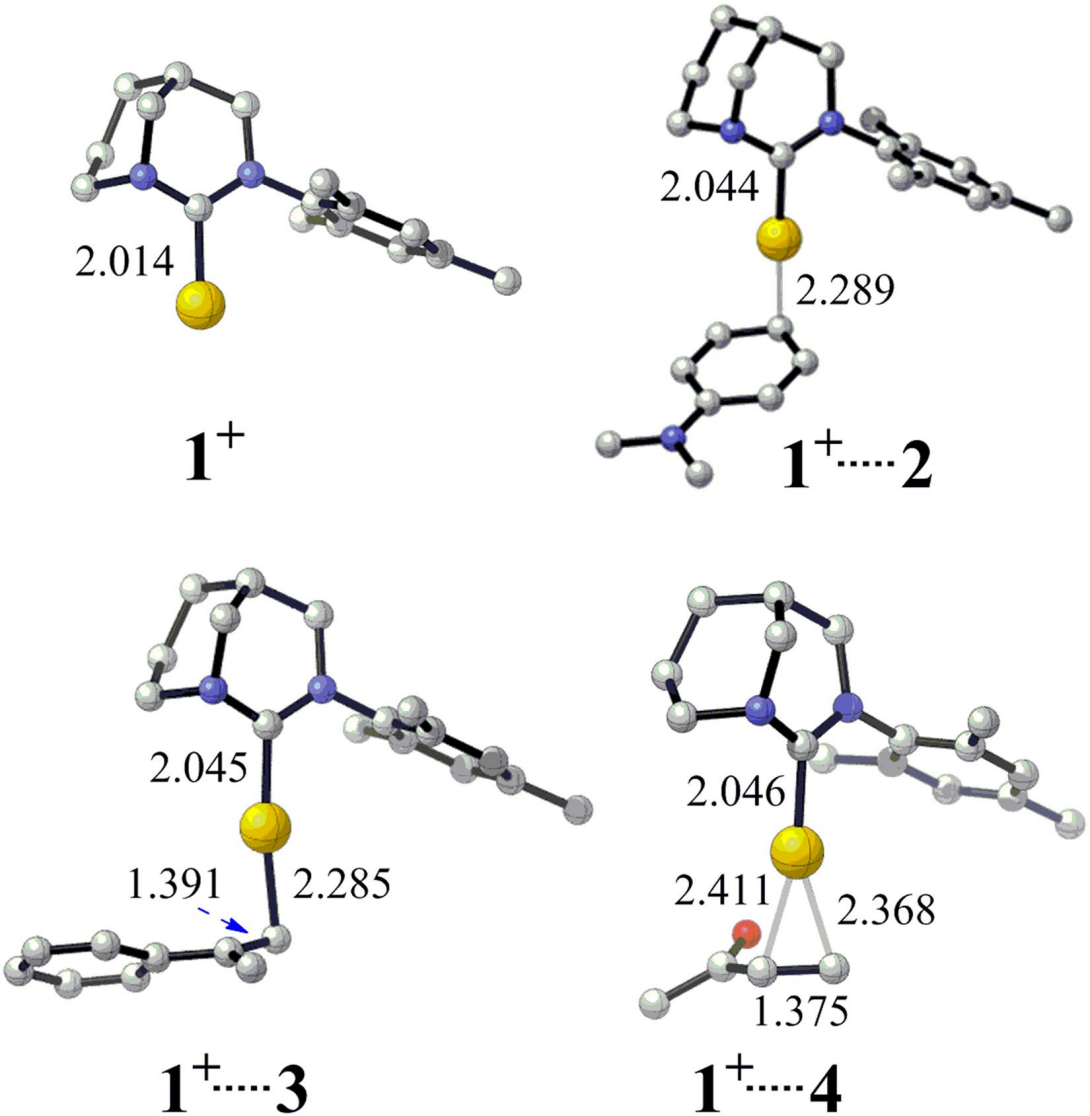
The optimized structures of **1**^**+**^, **1**^**+…**^**2**, **1**^**+…**^**3** and **1**^**+…**^**4** (Bond length in Å). All hydrogen atoms have been omitted for clarity.

According to the experimental results, the conversion of the hydroarylation between N,N-diethylaniline and α-methylstyrene is zero when only **1** is used as catalyst. However, the conversion is 97% when chlorine scavenger reagent KBArF was added in the presence of **1**^33^. It suggests that the active center in the reaction should be anti-Bredt carbene-Au^+^ cation **1**^**+**^ (Fig. 2). Similarly, other kinds of carbene-Au^+^ cations have been proposed to be the active catalytic species in a series of reactions^52–55^.

### The reaction between 2 and 3

**Figure 3:**
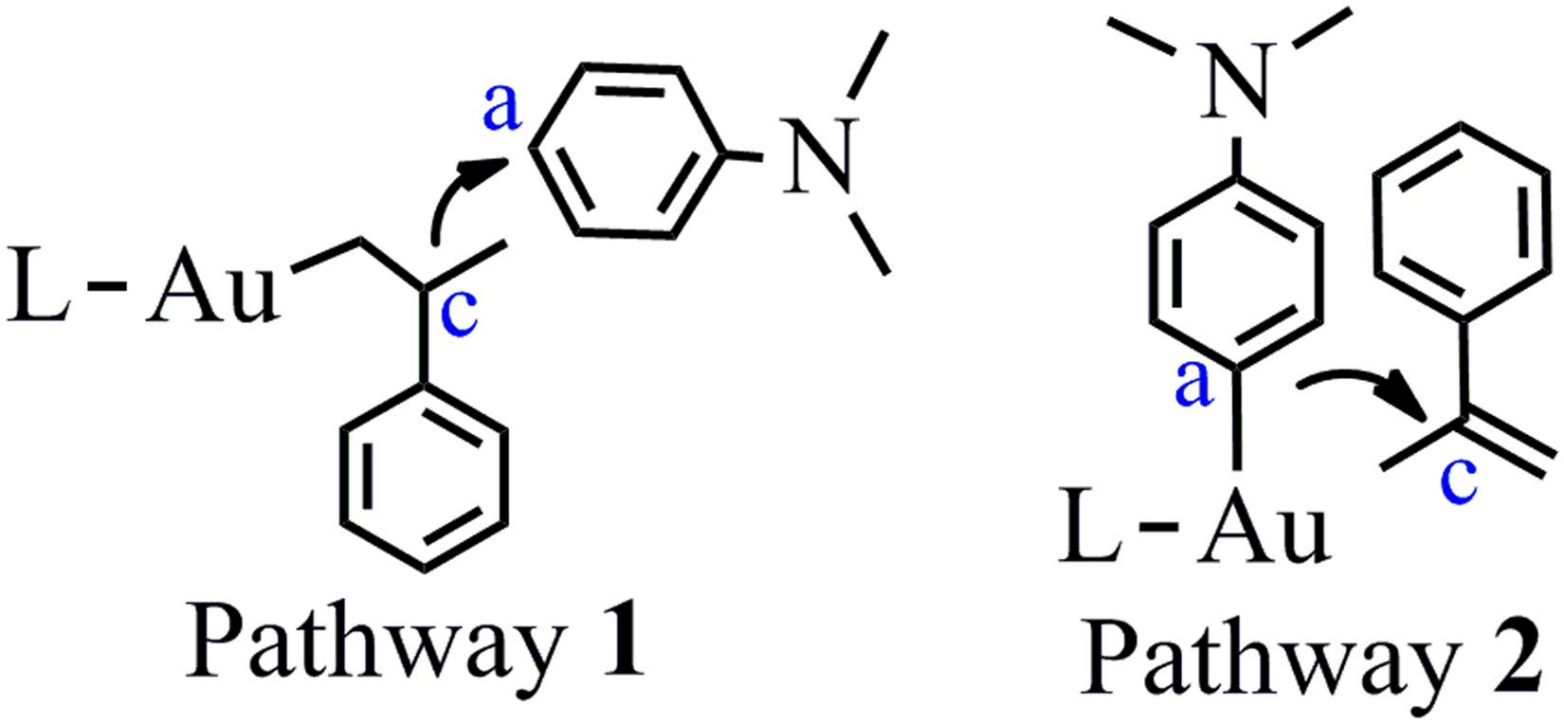
Different substrate activation models.

**5a** is the main product in the reaction between N,N-Dimethylaniline **2** and alkene **3**^33^. There has two possible pathways to produce **5a** (Fig. 3). Pathway 1: the gold cation activates the alkene **3**. Then, the electrophilic alkene attacks the aromatic ring. Pathway 2: the gold cation activates the N,N-Dimethylaniline **2**.

As we known, FC reaction is a kind of electrophilic aromatic substitution reactions, in which the hydrogen atom of aromatic ring is replaced by an electrophile^1,56–59^. The complex **1**^**+…**^**3** can serve as the electrophilic reagent (Fig. 2). It has been known that the gold cation has strong ability to bind alkene^60,61^. The binding between alkene **3** and catalyst **1**^**+**^ is highly exothermic (33.6 kcal/mol). The C=C bond length of alkene **3** was increased from 1.347 to 1.391 Å, which indicates that the C=C bond was activated after binding with **1**^**+**^.

**Figure 4:**
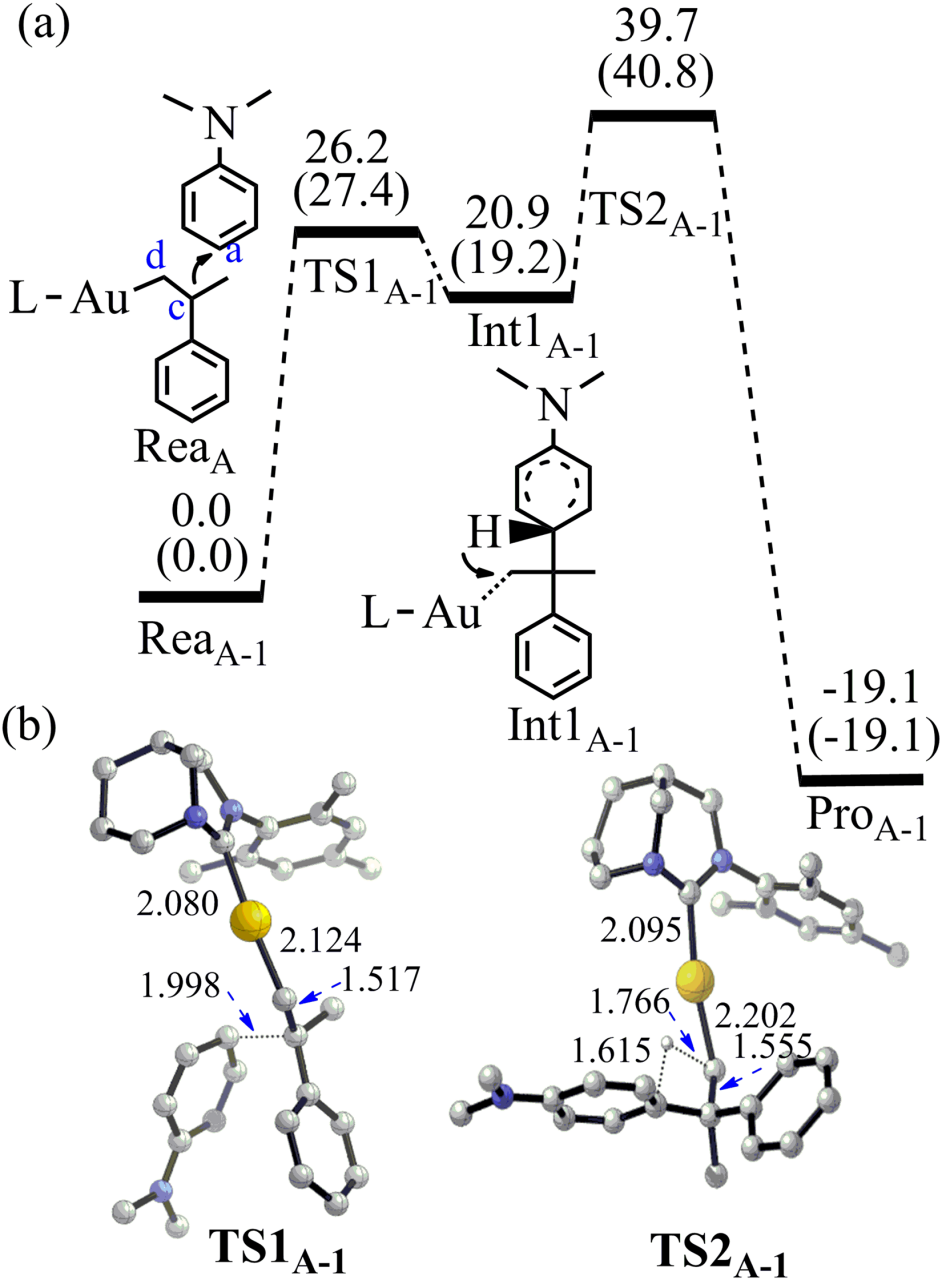
(**a**) The pathway 1 to produce **5a** by the Friedel-Crafts reaction between **2** and **3** without the assistance of aromatic amine (Energy in kcal/mol). Energies out of parenthesis were obtained at M06-2X (6–311 + G*/LANL2DZ). Energies in parenthesis were obtained at M06–2X (6–311 + G*/LANL2DZ) by PCM calculation. (**b**) The structures of the optimized transition states (Bond length in Å). All hydrogen atoms (except the transferred H) have been omitted for clarity.

For pathway 1, both C_c_ and C_d_ of alkene **3** attacking the C_a_ of N,N-dimethylaniline **2** were taken into consideration, which can produce branched and linear product (**5a** and **5b**) respectively. In the case of C_c_ attacking, the activation energy for the C_a_–C_c_ bond formation (TS1_A-1_) is 27.4 kcal/mol and the C_a_–C_c_ bond length in TS1_A-1_ is 1.998 Å (Energies obtained at M06-2X (6–311 + G*/LANL2DZ) by PCM calculation were discussed if not mentioned) (Fig. 4). At the same time, the C=C double bond of alkene **3** becomes almost single bond (bond length increases from 1.391 to 1.517 Å) in TS1_A-1_. An unstable intermediate (Int1_A-1_) is produced via TS1_A-1_ and the C_a_–C_c_ bond length of Int1_A-1_ is further reduced to 1.647 Å. The formation of C_a_–C_c_ bond makes the C_a_-H bond active. Meanwhile, the C_c_–C_d_ bond becomes single bond in Int1_A-1_ (length: 1.562 Å). Though TS2_A-1_ can lead to the final product **5a**, a direct proton transfer from C_a_ to C_d_ is not energy favorable due to the high overall activation energy (40.8 kcal/mol).

**Figure 5:**
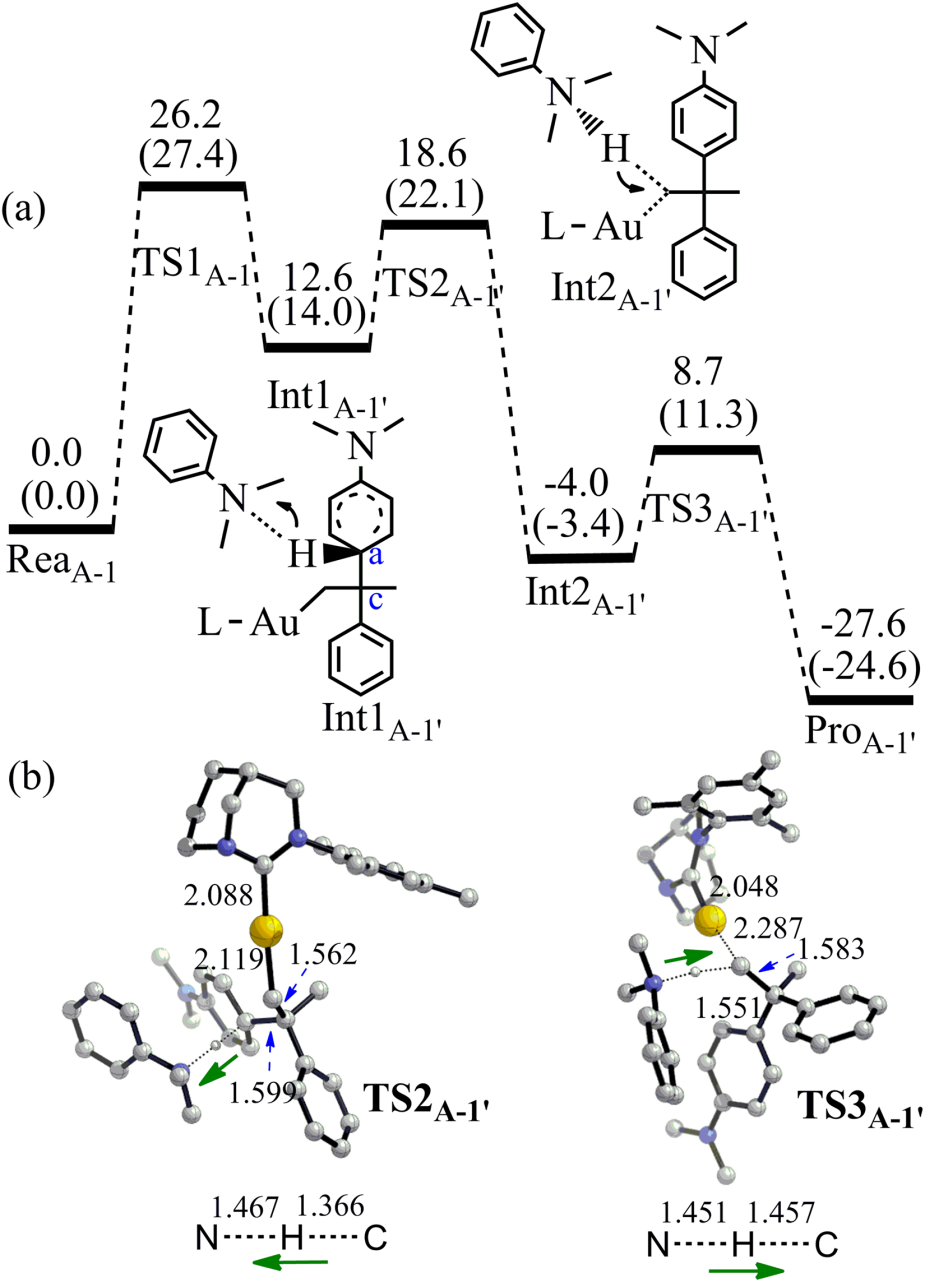
(**a**) The pathway 1 to produce **5a** by the Friedel-Crafts reaction between **2** and **3** with the assistance of aromatic amine (Energy in kcal/mol). Energies out of parenthesis were obtained at M06-2X (6–311 + G*/LANL2DZ). Energies in parenthesis were obtained at M06-2X (6–311 + G*/LANL2DZ) by PCM calculation. (**b**) The structures of the optimized transition states (Bond length in Å). All hydrogen atoms (except the transferred H) have been omitted for clarity.

It is worth to notice that there is plenty of aromatic amines in the microenvironment^33^. The aromatic amine is a good proton acceptor. It can abstract the proton of C_a_-H in Int1_A-1_ with a barrier of 22.1 kcal/mol (TS2_A-1′_) (Fig. 5). Then, the intermediate Int2_A-1′_ can re-abstract the proton of ammonium via TS3_A-1′_ and the corresponding energy barrier is 14.7 kcal/mol. The re-abstracting proton process is vital for the catalytic cycle. If the catalyst cannot re-abstract the proton from the ammonium, the reaction will be terminated. As an example, when 25 mol% proton-trapping reagent, 2,6-di-t-butyl-4-methylpyridine, was added in the FC alkylation of aniline catalyzed by acid, no any alkylation reaction can be observed^26^. TS3_A-1′_ can lead to the final product **5a** and the overall energy change from Rea_A-1_ to Pro_A-1′_ is −24.6 kcal/mol.

As indicated above, N,N-dialkylaniline is not only reactant but also a promoter. It should be the reason why excess N,N-dialkylaniline is necessary for the reaction^33^. The rate determining step is the C_a_–C_c_ bond formation process via the transition state TS1_A-1_ (barrier: 27.4 kcal/mol). This barrier height is reasonable considering that the reaction requires a temperature of 135 °C^33^.

**Figure 6:**
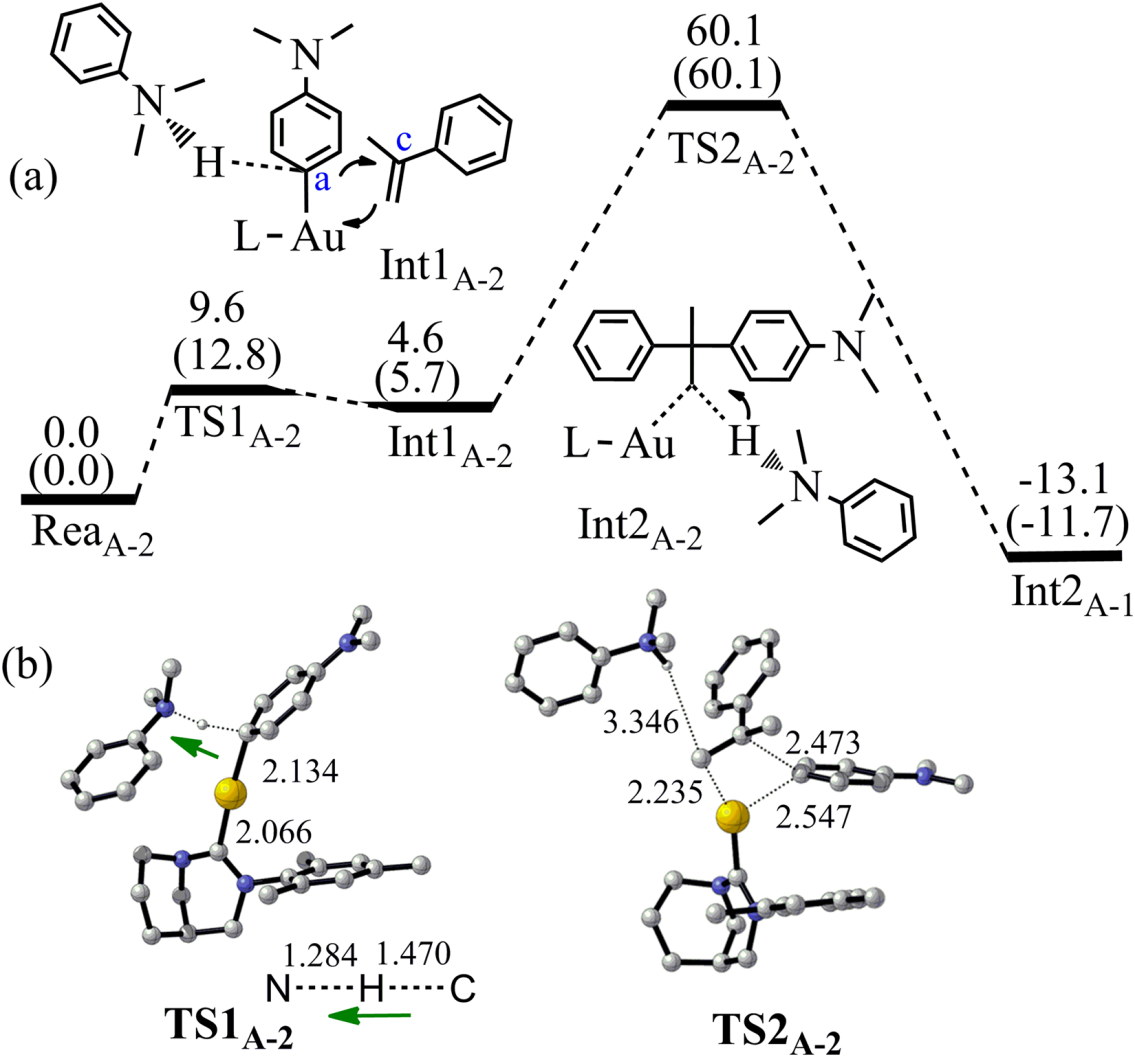
(**a**) The pathway 2 to produce **5a** by the Friedel-Crafts reaction between **2** and **3** (Energy in kcal/mol). Energies out of parenthesis were obtained at M06-2X (6–311 + G*/LANL2DZ). Energies in parenthesis were obtained at M06-2X (6–311 + G*/LANL2DZ) by PCM calculation. (**b**) The structures of the optimized transition states (Bond length in Å). All hydrogen atoms (except the transferred H) have been omitted for clarity.

Producing **5a** by the pathway that the gold cation activates the N,N-Dimethylaniline **2** and the C_a_ carbon of **2** attacks the C_c_ of alkene **3** is not possible because of the extremely high activation energy (Figs 3 and 6). The binding energy between **2** and **1**^**+**^ is −35.3 kcal/mol. This binding can increase the positive charge on the C_a_–H proton from 0.112 to 0.214. It makes the C_a_–H proton quite active. The external N,N-dialkylaniline **2** can abstract the C_a_–H proton with quite low activation barrier (12.8 kcal/mol, TS1_A-2_). However, the following C_a_–C_c_ bond formation is extremely energy-unfavorable and the corresponding activation barrier is 60.1 kcal/mol. It indicates that producing **5a** by gold cation activated N,N-Dimethylaniline **2** is not possible. Hence, this pathway is not taken into consideration for the producing of **5b**, **5c**, **6a** and **6b**.

**Figure 7:**
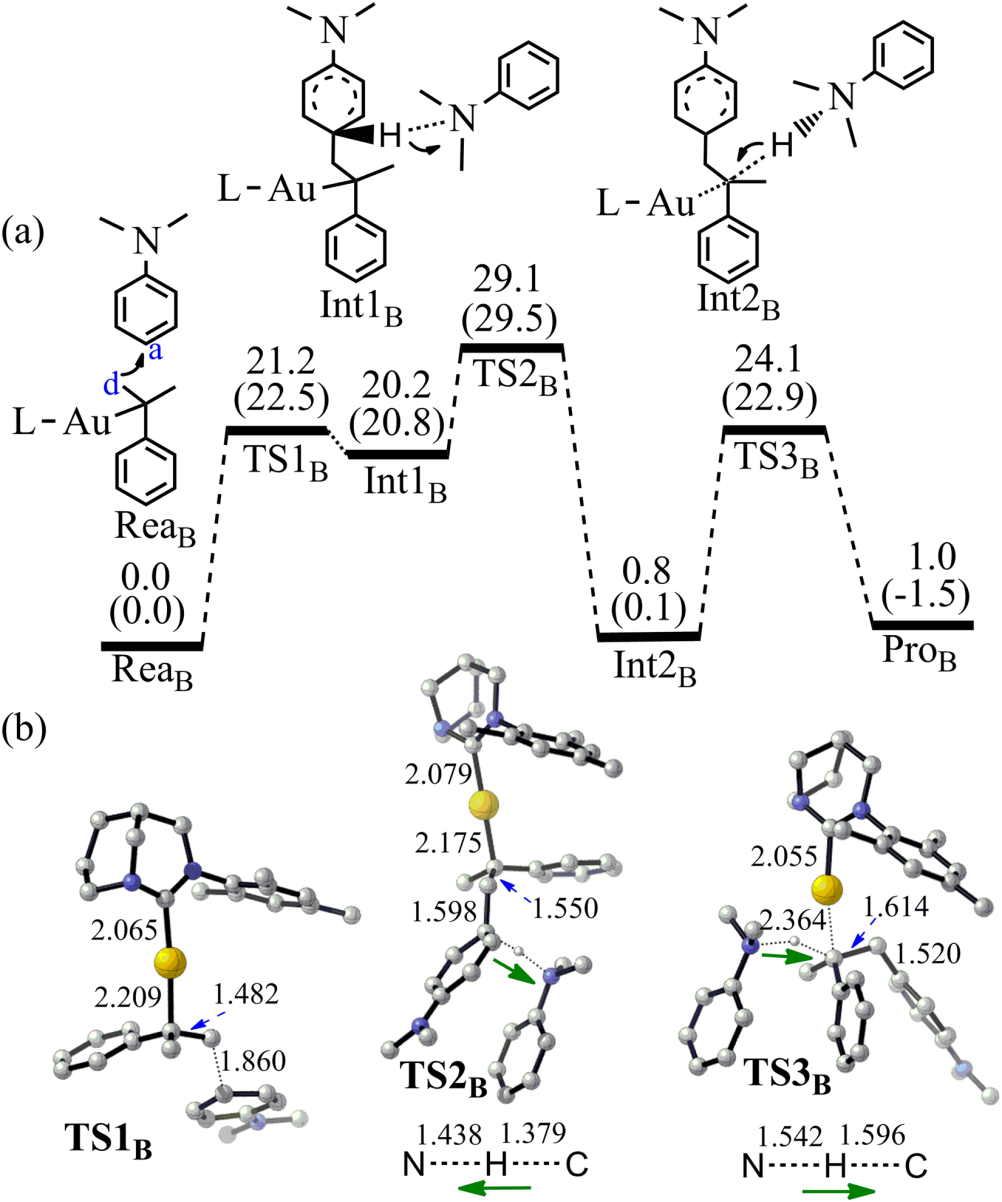
(**a**) The pathway to produce **5b** by the Friedel-Crafts reaction between **2** and **3** (Energy in kcal/mol). Energies out of parenthesis were obtained at M06-2X (6–311 + G*/LANL2DZ). Energies in parenthesis were obtained at M06-2X (6–311 + G*/LANL2DZ) by PCM calculation. (**b**) The structures of the optimized transition states (Bond length in Å). All hydrogen atoms (except the transferred H) have been omitted for clarity.

If the alkene terminal carbon C_d_ can attack the C_a_ of N,N-dialkylaniline **2** (Fig. 7), anti-Markovnikov product **5b** should be obtained. The activation energy for the C_a_-C_d_ bond formation is only 22.5 kcal/mol (TS1_B_). Though the barrier height of TS1_B_ is lower than that of TS1_A-1_, the proton abstracting process via TS2_B_ is not easy for this anti-Markovnikov reaction. The overall activation energy for the process to produce **5b** is 29.5 kcal/mol. Furthermore, the energy change from Rea_B_ to Pro_B_ is only −1.5 kcal/mol. Hence, comparing with the producing of **5a**, the generation of the linear product **5b** is difficult because the higher activation energy and quite small free energy change. Indeed, no **5b** was observed for the FC reaction between **2** and **3** in our previous experiment^33^.

**Figure 8:**
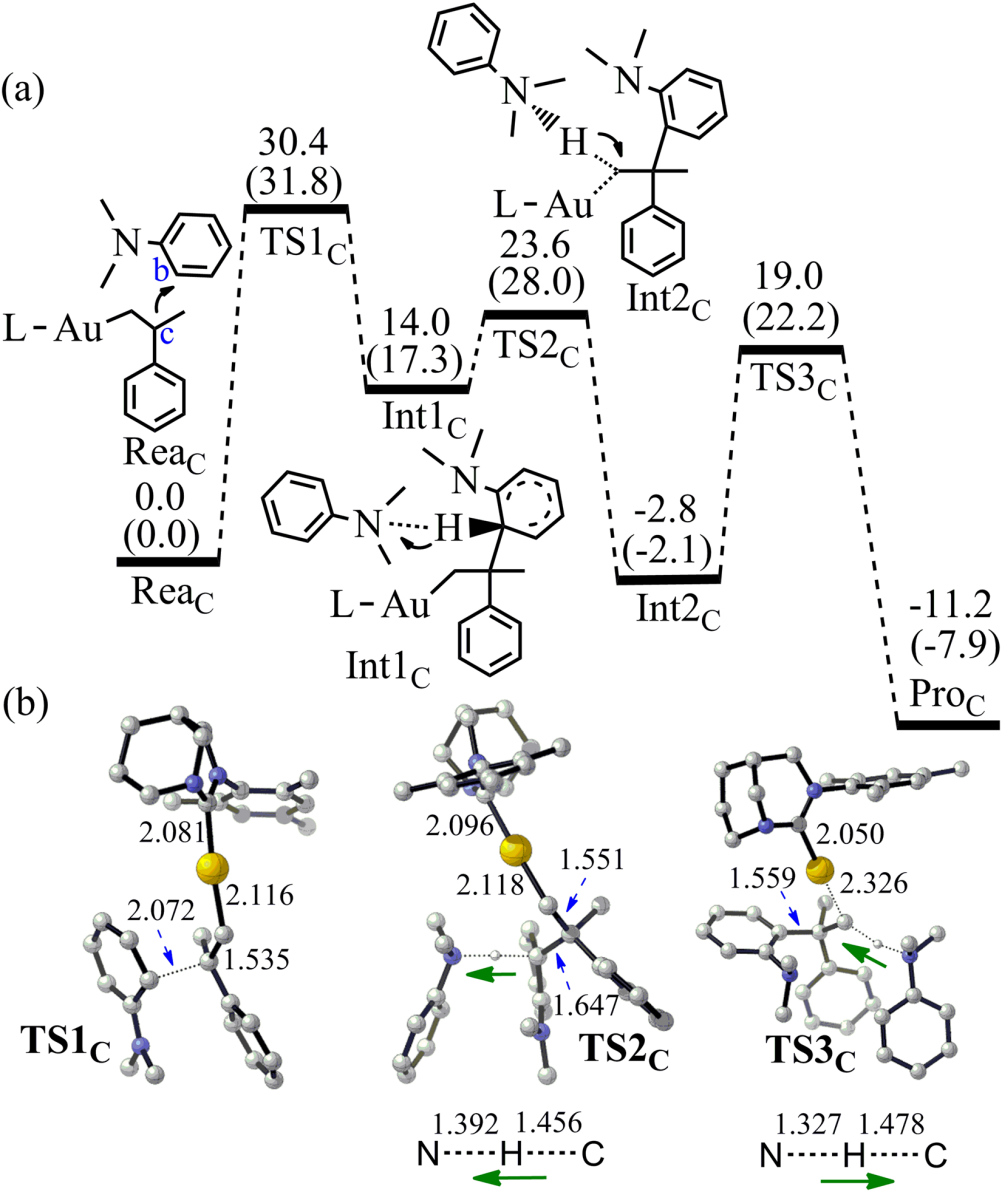
(**a**) The pathway to produce **5c** by the Friedel-Crafts reaction between **2** and **3** (Energy in kcal/mol). Energies out of parenthesis were obtained at M06-2X (6–311 + G*/LANL2DZ). Energies in parenthesis were obtained at M06-2X (6–311 + G*/LANL2DZ) by PCM calculation. (**b**) The structures of the optimized transition states (Bond length in Å). All hydrogen atoms (except the transferred H) have been omitted for clarity.

Most of the known FC reactions gave both *para*- and *ortho*-products^1,62,63^. Interestingly, for the reaction between N,N-Dimethylaniline **2** and alkene **3**, quite high *para*-selectivity was obtained and **5a** was isolated with 93% yield^33^. Due to the steric hindrance of the –N(CH_3_)_2_ group, the C_c_ of alkene **3** attacking the C_b_ carbon of **2** is difficult (Fig. 8). The energy barrier for the C_b_-C_c_ bond formation is 31.8 kcal/mol (TS1_C_). Furthermore, the access of extra aromatic amine is not easy due to the steric hindrance, which makes the abstracting proton from C_b_ not easy comparing with the process to produce **5a**. The free energy change from Rea_c_ to Pro_c_ is −7.9 kcal/mol. Considering that the barrier of the rate determining step to produce **5c** is 4.4 kcal/mol higher than that to produce **5a**, observing a minute quantity of **5c** as byproduct is reasonable^33^.

### The reaction between 2 and 4

The reaction of olefine ketone **4** is quite different from that of aromatic alkene **3**. For aromatic alkene **3**, the C_a_ of N,N-Dimethylaniline **2** and C_c_ of **3** forms C–C bond and the producing of **5a** follows the Markovnikov rule. However, the reaction of olefine ketone **4** follows the anti-Markovnikov rule. The C–C bond was formed between the C_a_ of **2** and the terminal carbon C_d_ of **4** to produce **6a** (Fig. 1)^33^. To fully understand this difference, both C_c_ and C_d_ of **4** attacking the C_a_ of **2** were taken into consideration, which can produce branched and linear product (**6b** and **6a**) respectively. The binding between **4** and **1**^**+**^ can form the electrophilic group and this process releases 25.5 kcal/mol energy. The C=C bond length of olefine ketone **4** was increased from 1.341 to 1.375 Å during the formation of **1**^**+…**^**4** (Fig. 2).

**Figure 9:**
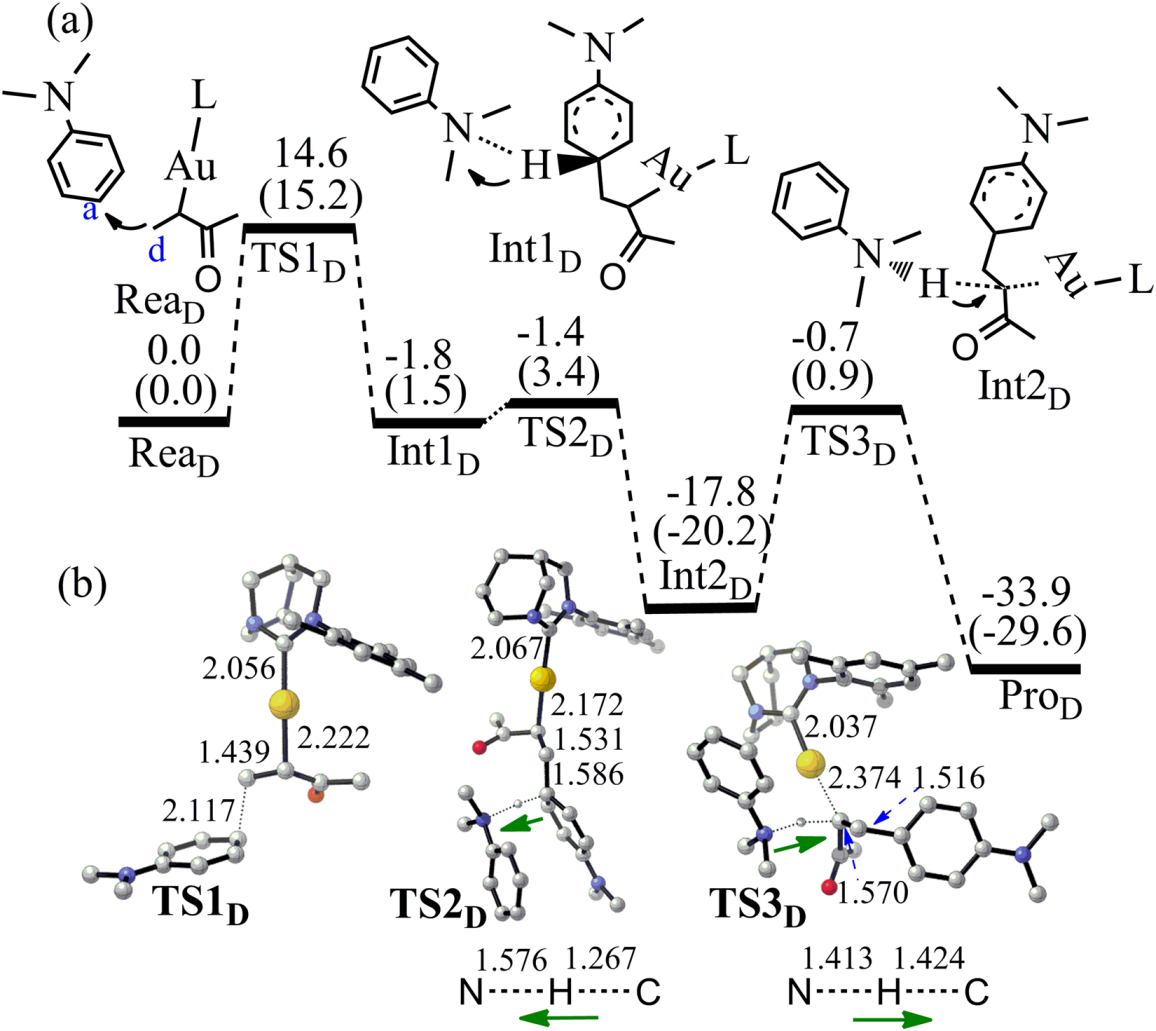
(**a**) The pathway to produce **6a** by the Friedel-Crafts reaction between **2** and **4** (Energy in kcal/mol). Energies out of parenthesis were obtained at M06-2X (6–311 + G*/LANL2DZ). Energies in parenthesis were obtained at M06-2X (6–311 + G*/LANL2DZ) by PCM calculation. (**b**) The structures of the optimized transition states (Bond length in Å). All hydrogen atoms (except the transferred H) have been omitted for clarity.

In the case of C_d_ attacking, the activation energy for the C_a_-C_d_ bond formation (TS1_D_) is only 15.2 kcal/mol and the C_a_-C_d_ bond length is 2.117 Å in TS1_D_ (Fig. 9). During the C_a_-C_d_ bond formation, the C=C double bond of **4** was increased from 1.375 to 1.439 Å. For the reaction of aromatic alkene **3**, this bond was increased by 0.126 Å (from Rea_A-1_ to TS1_A-1_). As we know, the structure distortion can induce energy change and enhance the reaction barrier^64^. Correspondingly, the energy barrier of TS1_D_ is 12.2 kcal/mol lower than that of the same process to produce **5a** (TS1_A_). The smaller structure change is responsible for the low barrier of TS1_D_. Similarly, proton abstracting by aromatic amine and re-abstracting the proton of ammonium happen for the producing of **6a**. The proton re-abstracting process from the ammonium is the rate determining step and the corresponding activation energy is 21.1 kcal/mol (TS3_D_).

**Figure 10:**
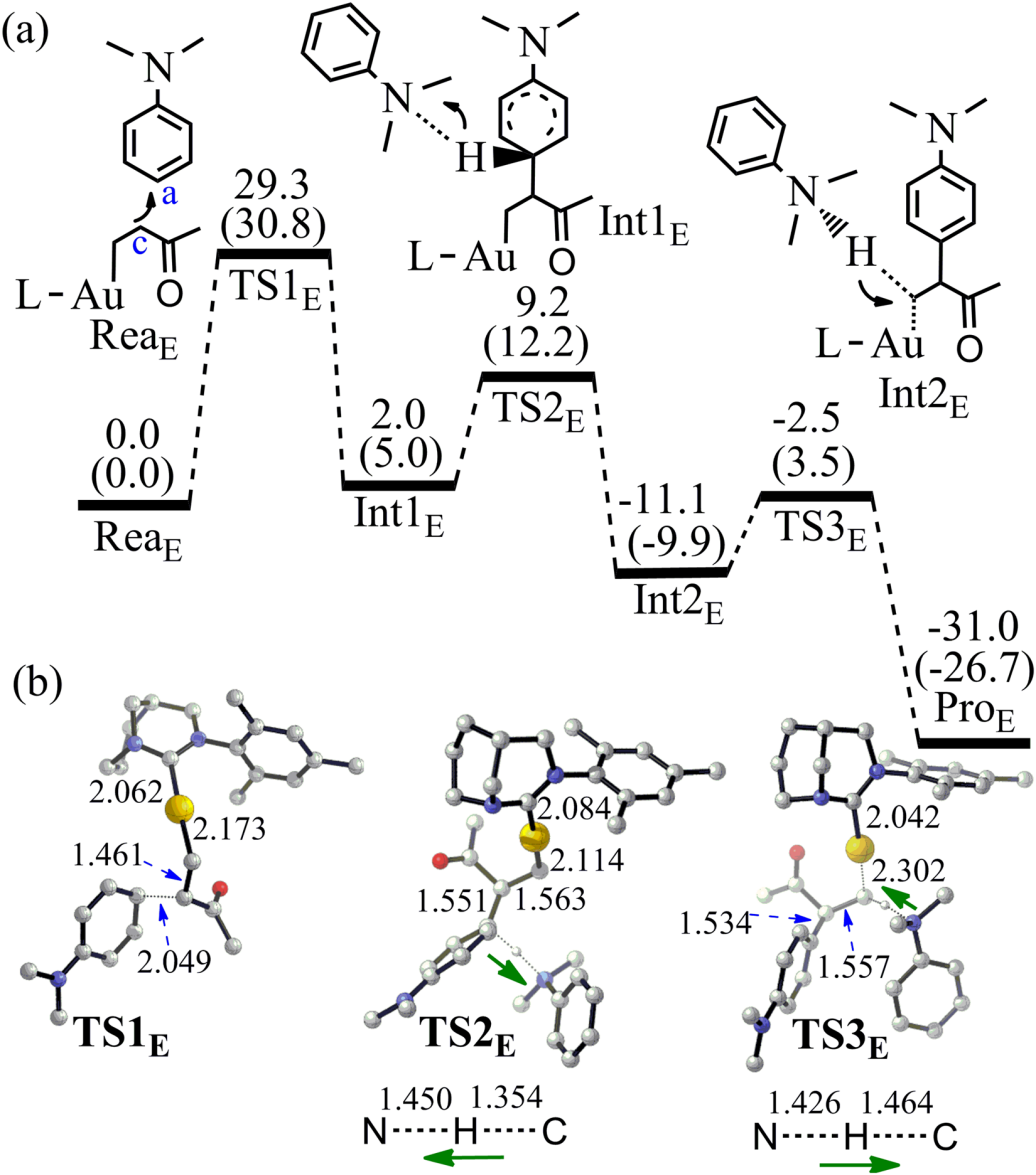
(**a**) The pathway to produce **6b** by the Friedel-Crafts reaction between **2** and **4** (Energy in kcal/mol). Energies out of parenthesis were obtained at M06-2X (6–311 + G*/LANL2DZ). Energies in parenthesis were obtained at M06-2X (6–311 + G*/LANL2DZ) by PCM calculation. (**b**) The structures of the optimized transition states (Bond length in Å). All hydrogen atoms (except the transferred H) have been omitted for clarity.

If the C_c_ of olefine ketone **4** can attack the C_a_ of N,N-dialkylaniline **2** (Fig. 10), Markovnikov product **6b** should be observed in the experiment. In the case of C_c_ attacking, the activation energy for the C_a_–C_c_ bond formation is quite high (TS1_E_: 30.8 kcal/mol). At the same time, the C=C double bond of **3** becomes almost single bond in TS1_E_ (increasing from 1.375 to 1.461 Å). The proton abstracting by the aromatic amine and re-abstracting from the ammonium is easy to achieve in this pathway. However, the producing **6b** is not competitive to **6a**. The activation energy of the rate determining step to produce **6b** is 9.7 kcal/mol higher than that of **6a**. That is why the anti-Markovnikov linear product **6a** was observed in the experiment^33^.

## Conclusion

The Friedel-Crafts alkylation of N,N-dimethylaniline with alkenes catalyzed by cyclic diaminocarbene-Gold(I) complex were theoretically investigated. The calculation method adopted here is accurate enough to reproduce the crystal structure of the catalyst. The gold cation can activate the C=C double bond to produce the electrophilic [R-C=C^…^Au-L]^+^. Then, the [R-C=C^…^Au-L]^+^ attacks the aromatic ring, following the electrophilic aromatic substitution mechanism. Being different from previous result that alkaline arenes will trap the proton and the reaction will be terminated as result^26^, herein, it was found that the alkaline N,N-dimethylaniline can assist the reaction. Based on the obtained reaction mechanism, we can well understand why the reaction was high *para*-selevtivity, and why branched and linear products were obtained for different substrates: (1) Producing the *para*-product is more energy favorable comparing with the *ortho*-product. (2) The reaction of α-methylstyrene follows the Markovnikov rule. The activation energy to generate the branched product of α-methylstyrene is lower than that producing the linear product. Besides, the reaction leading to branched product is highly exothermic. (3) The reaction of butanone follows the anti-Markovnikov rule. The activation energy to generate the branched product of butanone is higher than that producing the linear product.

These theoretical results are quite useful for designing more effective catalysts for this rare FC reaction using alkaline arenes as substrates. Based on the understanding of the reaction mechanism, the development of none-noble metal catalyst for this FC reaction is on going in our group.

### Computational Methods

All the structures were fully optimized with B3LYP based on Density Functional Theory (DFT) (See SI for structure details). This method is a good choice for the calculation of organometallic systems^65–75^. The following combination of basis sets were used for geometric configuration optimization and frequency calculation: 6–31G basis set for all atoms except Au and LANL2DZ basis set for Au (abbreviated as 6–31G/LANL2DZ)^41,75^. LANL2DZ basis set includes the relativistic effect of the heavy element^65,76^. The calculation method adopted here can well reproduce the crystal structure of the carbene-gold used for the FC reaction of basic arenes with alkenes (Table 1). Because B3LYP method often suffers from incorrect energies, especially for systems containing non covalent bonds. A higher level method M06-2X was used to generate more accurate energies. The energy calculations were performed using 6–311 + G* basis set for all atoms except Au and LANL2DZ basis set for Au (abbreviated as 6–311 + G*/LANL2DZ). The influence of solvent was performed in condensed phase with the Polarizable Continuum Model (PCM) using 6–311 + G*/LANL2DZ basis set. This method creates the solute cavity via a set of overlapping spheres. Aniline was used as solvent to simulate the environment of N,N-dimethylaniline.

The computed stationary points have been characterized as minima or transition states by diagonalizing the Hessian matrix and analyzing the vibrational normal modes. In this way, the stationary points can be classified as minima if no imaginary frequencies are shown or as transition states if only one imaginary frequency is obtained. The particular nature of the transition states has been determined by analyzing the motion described by the eigenvector associated with the imaginary frequency. All calculations were performed with the Gaussian 03 suite of programs^77^.

## Electronic supplementary material

Supplementary Information
