# Supplementary material for: Friedel-Crafts Reaction of N,N-Dimethylaniline with Alkenes Catalyzed by Cyclic Diaminocarbene-Gold(I) Complex
Source: Sci Rep. 2018 Jul 30;8:11449. doi: 10.1038/s41598-018-29854-0 (PMC6065310; doi:10.1038/s41598-018-29854-0)
Supplement: Supplementary file 1 — Supplementary Information [file 41598_2018_29854_MOESM1_ESM.doc]

**Friedel-Crafts Reaction of N,N-Dimethylaniline with Alkenes Catalyzed by Cyclic Diaminocarbene-Gold(I) Complex**

Hangzhi Wu, Tianxiang Zhao, and Xingbang Hu

**Cartesians coordinates of the optimized structures.**

1 (B3LYP)

Au 0 -1.483871 1.462631 0.268997

C 0 0.591873 -1.075592 2.276932

C 0 2.600021 -0.680466 0.800980

C 0 2.451248 -0.636322 -1.601425

C 0 3.227402 -0.510757 -0.441055

C 0 0.485611 -1.089111 -0.279190

C 0 0.267044 -1.010223 -2.817444

C 0 1.231476 -0.964783 0.910736

C 0 1.080200 -0.920410 -1.545425

Cl 0 -1.063965 3.770248 0.774948

N 0 -3.171234 -0.892563 -0.389954

N 0 -0.928879 -1.426529 -0.221756

C 0 -1.225464 -2.893967 -0.060028

C 0 -4.143555 -0.661942 0.710831

C 0 -3.757568 -1.646988 1.842545

C 0 -3.354752 -2.222687 -1.014191

C 0 -2.734863 -3.219727 -0.029724

C 0 -1.877960 -0.465652 -0.155731

C 0 4.696289 -0.161392 -0.525300

C 0 -3.490433 -3.093689 1.331436

H 0 0.133423 -2.056763 2.452896

H 0 -0.190262 -0.317657 2.404984

H 0 1.337952 -0.918533 3.060669

H 0 3.183792 -0.572600 1.710617

H 0 2.917877 -0.499405 -2.572754

H 0 0.906967 -0.870438 -3.693008

H 0 -0.512070 -0.239136 -2.845642

H 0 -0.232330 -1.981949 -2.923447

H 0 -0.721329 -3.226395 0.853149

H 0 -0.758423 -3.414871 -0.903046

H 0 -5.150421 -0.841919 0.322403

H 0 -4.073624 0.376236 1.043777

H 0 -2.854568 -1.252945 2.326996

H 0 -4.540863 -1.674271 2.609665

H 0 -2.854187 -2.233795 -1.986938

H 0 -4.423737 -2.381760 -1.172043

H 0 -2.847275 -4.255402 -0.374542

H 0 5.260986 -0.596375 0.306015

H 0 4.842865 0.926208 -0.485550

H 0 5.139969 -0.516449 -1.461243

H 0 -4.457047 -3.599686 1.193253

H 0 -2.956818 -3.658188 2.106430

1 (M06-2X)

Au 0 -1.468567 1.698563 0.599533

C 0 0.425629 -0.744385 2.563583

C 0 2.482910 -0.371552 1.151154

C 0 2.395874 -0.367767 -1.252579

C 0 3.134506 -0.214905 -0.075500

C 0 0.418381 -0.826340 0.024139

C 0 0.227921 -0.778076 -2.494186

C 0 1.120438 -0.670395 1.227249

C 0 1.033127 -0.669144 -1.224167

Cl 0 -0.935989 3.972687 1.161011

N 0 -3.217072 -0.620132 -0.061249

N 0 -0.986420 -1.167938 0.053574

C 0 -1.269562 -2.626328 0.183426

C 0 -4.131073 -0.382263 1.077072

C 0 -3.661774 -1.342209 2.189667

C 0 -3.425186 -1.943619 -0.670609

C 0 -2.769882 -2.938080 0.287109

C 0 -1.920624 -0.208679 0.142380

C 0 4.596176 0.152863 -0.124847

C 0 -3.444931 -2.790558 1.678497

H 0 -0.294632 -1.567017 2.619385

H 0 -0.127000 0.184464 2.753250

H 0 1.152267 -0.873322 3.368347

H 0 3.043411 -0.240677 2.072113

H 0 2.887100 -0.236282 -2.212021

H 0 0.869156 -0.651749 -3.368362

H 0 -0.548600 -0.005707 -2.527008

H 0 -0.271912 -1.749619 -2.579773

H 0 -0.716919 -2.983337 1.059250

H 0 -0.851410 -3.126282 -0.697281

H 0 -5.152435 -0.587997 0.747391

H 0 -4.060776 0.661835 1.390518

H 0 -2.718872 -0.941818 2.586456

H 0 -4.376297 -1.356064 3.018448

H 0 -2.959503 -1.958679 -1.660522

H 0 -4.498810 -2.103348 -0.785507

H 0 -2.910004 -3.972702 -0.043640

H 0 5.152451 -0.320663 0.688248

H 0 4.723267 1.236115 -0.025452

H 0 5.049332 -0.149368 -1.071991

H 0 -4.425720 -3.278534 1.598251

H 0 -2.876846 -3.356003 2.425919

1^+…^4

Au 0 0.185852 0.154358 -1.004333

C 0 -1.651169 -0.514420 2.156952

C 0 -2.781982 -2.512665 1.128174

C 0 -3.835846 -2.454742 -1.037354

C 0 -3.476486 -3.179932 0.111115

C 0 -2.837341 -0.457718 -0.126450

C 0 -3.941208 -0.350235 -2.430557

C 0 -2.448517 -1.151077 1.038223

C 0 -3.532990 -1.095688 -1.178406

N 0 -1.343140 2.741959 -1.111496

N 0 -2.559433 0.969100 -0.255203

C 0 -3.571457 1.891708 0.384903

C 0 -0.302994 3.564941 -0.431458

C 0 -0.765381 3.713303 1.040390

C 0 -2.635422 3.467789 -1.221069

C 0 -3.253159 3.389664 0.180283

C 0 -1.405457 1.418808 -0.767014

C 0 -3.854431 -4.636414 0.252869

C 0 -2.272400 4.083313 1.178452

H 0 -1.762558 0.572037 2.197479

H 0 -0.580734 -0.729889 2.042007

H 0 -1.959030 -0.918854 3.126053

H 0 -2.489227 -3.060165 2.019333

H 0 -4.369247 -2.957443 -1.838669

H 0 -4.451263 -1.021300 -3.126053

H 0 -3.077286 0.076233 -2.954727

H 0 -4.629639 0.476181 -2.212646

H 0 -3.610886 1.619492 1.444580

H 0 -4.544128 1.654617 -0.056259

H 0 -0.234070 4.522873 -0.952682

H 0 0.665390 3.060089 -0.498795

H 0 -0.568329 2.760086 1.549774

H 0 -0.161102 4.472244 1.550247

H 0 -3.252762 2.981934 -1.981613

H 0 -2.425339 4.489441 -1.541565

H 0 -4.206696 3.928238 0.227112

H 0 -3.210571 -5.150635 0.972458

H 0 -3.786407 -5.163986 -0.704117

H 0 -4.888779 -4.739777 0.606415

H 0 -2.382400 5.163986 1.014572

H 0 -2.594116 3.896759 2.209732

C 0 1.402832 -1.817657 -1.489685

C 0 2.338318 -0.930695 -1.012070

C 0 2.756012 -0.941162 0.440842

C 0 4.160309 -0.498367 0.744278

O 0 1.959290 -1.322006 1.316422

H 0 1.237015 -1.945969 -2.555145

H 0 0.930161 -2.531326 -0.819504

H 0 2.940262 -0.346130 -1.704102

H 0 4.344513 -0.559967 1.817398

H 0 4.888779 -1.126740 0.215469

H 0 4.322800 0.532745 0.403748

1^+…^3

Au 0 0.134674 -0.751877 0.013596

C 0 -3.842285 -0.595108 0.344269

C 0 -3.896362 -2.932114 -0.598770

C 0 -2.663452 -3.571289 -2.566269

C 0 -3.542923 -3.919327 -1.529129

C 0 -2.531082 -1.308853 -1.742218

C 0 -1.201385 -1.943268 -3.829910

C 0 -3.411484 -1.618210 -0.684723

C 0 -2.146423 -2.275894 -2.695892

N 0 -0.311493 1.605560 -1.779953

N 0 -2.018539 0.047653 -1.888260

C 0 -2.965988 1.040070 -2.520615

C 0 -0.073990 2.644409 -0.737991

C 0 -1.473206 3.148605 -0.303116

C 0 -0.878098 2.191238 -3.021957

C 0 -2.354416 2.447647 -2.701630

C 0 -0.842194 0.414093 -1.353867

C 0 -4.111023 -5.316650 -1.432083

C 0 -2.422241 3.444595 -1.501663

C 0 0.881729 -2.195724 1.619858

C 0 2.164627 -1.744064 1.327911

C 0 2.807877 -0.640564 2.084366

C 0 3.830856 0.137924 1.500107

C 0 2.436920 -0.368851 3.420349

C 0 4.444214 1.165604 2.219147

C 0 3.059128 0.651993 4.139694

C 0 4.061096 1.426132 3.540314

C 0 2.991653 -2.470215 0.288437

H 0 -4.695633 0.000977 -0.006622

H 0 -3.035919 0.098602 0.601608

H 0 -4.161575 -1.091019 1.265152

H 0 -4.567413 -3.186584 0.216446

H 0 -2.374100 -4.324326 -3.293243

H 0 -1.057205 -2.813324 -4.475464

H 0 -0.214828 -1.633438 -3.464432

H 0 -1.584534 -1.129364 -4.458176

H 0 -3.861725 1.063889 -1.892807

H 0 -3.251450 0.632477 -3.495193

H 0 0.540375 3.433151 -1.179825

H 0 0.471542 2.203339 0.100845

H 0 -1.918396 2.378174 0.341171

H 0 -1.374969 4.051666 0.310226

H 0 -0.742996 1.483063 -3.844437

H 0 -0.323746 3.102859 -3.251800

H 0 -2.872803 2.914627 -3.547318

H 0 -4.385895 -5.567657 -0.403152

H 0 -3.396530 -6.066360 -1.786560

H 0 -5.016388 -5.415436 -2.045471

H 0 -2.171188 4.433319 -1.909683

H 0 -3.452698 3.518494 -1.134552

H 0 0.547882 -3.153687 1.229568

H 0 0.341583 -1.823746 2.485977

H 0 4.140823 -0.045486 0.477814

H 0 1.689224 -0.981216 3.911865

H 0 5.223755 1.757600 1.752014

H 0 2.773865 0.834763 5.169922

H 0 4.546265 2.217178 4.101883

H 0 3.370514 -1.797867 -0.487473

H 0 3.865906 -2.921646 0.774139

H 0 2.416122 -3.260803 -0.197647

Rea_A-1_

Au 0 0.396851 -1.632797 0.158859

C 0 1.010712 -4.156540 -2.893326

C 0 -1.050674 -3.185822 -3.971664

C 0 -3.120529 -2.877060 -2.780441

C 0 -2.389450 -2.770096 -3.973465

C 0 -1.205292 -3.808029 -1.648621

C 0 -3.376892 -3.487335 -0.341873

C 0 -0.435303 -3.713898 -2.826721

C 0 -2.553589 -3.393066 -1.607834

N 0 0.202484 -4.186768 1.712234

N 0 -0.620300 -4.376328 -0.441208

C 0 -0.543732 -5.884277 -0.402618

C 0 1.622100 -4.194916 2.164352

C 0 2.361542 -5.199814 1.246353

C 0 -0.471664 -5.479553 1.996185

C 0 0.040222 -6.438660 0.915649

C 0 -0.054153 -3.599869 0.498032

C 0 -3.036423 -2.241837 -5.233307

C 0 1.592743 -6.540726 1.055161

C 0 0.929611 0.461792 -0.561462

C 0 0.787460 0.878235 0.759277

C 0 1.943634 0.941574 1.690323

C 0 1.764130 0.792740 3.082275

C 0 3.246567 1.186295 1.203247

C 0 2.852936 0.865143 3.953796

C 0 4.331131 1.269531 2.077591

C 0 4.139175 1.103943 3.455231

C 0 -0.546188 1.396423 1.241165

H 0 1.098785 -5.236572 -3.073044

H 0 1.556625 -3.927261 -1.972839

H 0 1.524872 -3.656540 -3.718872

H 0 -0.466339 -3.098923 -4.883118

H 0 -4.157074 -2.553802 -2.761063

H 0 -4.403793 -3.162704 -0.527939

H 0 -2.968872 -2.857834 0.458160

H 0 -3.422012 -4.513504 0.043839

H 0 0.046677 -6.192200 -1.270600

H 0 -1.562408 -6.259979 -0.541580

H 0 1.641052 -4.491455 3.216415

H 0 2.040863 -3.188660 2.076553

H 0 2.508423 -4.713928 0.272171

H 0 3.360474 -5.411545 1.644791

H 0 -1.554688 -5.334457 1.950012

H 0 -0.203979 -5.788254 3.008224

H 0 -0.369080 -7.447220 1.046890

H 0 -2.301270 -1.778183 -5.898285

H 0 -3.808090 -1.499222 -5.007339

H 0 -3.519516 -3.051910 -5.795670

H 0 1.780014 -7.177917 1.930481

H 0 2.010834 -7.075363 0.194031

H 0 0.136307 0.671814 -1.273788

H 0 1.914139 0.285645 -0.986800

H 0 0.776886 0.603867 3.487717

H 0 3.405441 1.352859 0.143845

H 0 2.697311 0.741333 5.020126

H 0 5.321915 1.475937 1.687485

H 0 4.982620 1.171005 4.134033

H 0 -0.859802 0.934998 2.182419

H 0 -0.458740 2.476242 1.420868

H 0 -1.329269 1.233841 0.497971

C 0 1.307541 4.639023 0.087097

C 0 0.523834 4.498900 -1.065430

C 0 -0.845947 4.768295 -1.045883

C 0 -1.484650 5.199707 0.146240

C 0 -0.681717 5.336334 1.309677

C 0 0.687485 5.061813 1.269867

N 0 -2.845612 5.481750 0.173264

C 0 -3.465805 5.991180 1.396423

C 0 -3.635971 5.400009 -1.054291

H 0 2.374847 4.448120 0.060211

H 0 0.984497 4.192520 -2.000549

H 0 -1.413803 4.665176 -1.961716

H 0 -1.120880 5.679321 2.237793

H 0 1.275894 5.194077 2.173157

H 0 -4.530685 6.147293 1.219589

H 0 -3.363586 5.280487 2.227814

H 0 -3.029007 6.948975 1.713135

H 0 -3.273819 6.093765 -1.826538

H 0 -3.622437 4.385895 -1.477127

H 0 -4.672470 5.655090 -0.830566

TS1_A-1_

Au 0 -0.769119 -1.320251 1.835739

C 0 2.032272 -3.098282 -0.470000

C 0 0.355118 -3.151260 -2.356949

C 0 -1.910797 -3.905045 -2.049957

C 0 -0.912003 -3.406586 -2.900009

C 0 -0.376389 -3.910461 -0.187485

C 0 -2.775696 -4.686417 0.192703

C 0 0.649673 -3.398193 -1.007141

C 0 -1.667633 -4.165405 -0.694717

N 0 -0.266403 -3.814102 3.472244

N 0 -0.102631 -4.227585 1.205704

C 0 0.568665 -5.559067 1.432068

C 0 0.766739 -3.227478 4.365768

C 0 2.132996 -3.765396 3.868484

C 0 -0.376968 -5.281815 3.653915

C 0 0.831955 -5.871124 2.921005

C 0 -0.302400 -3.316986 2.182922

C 0 -1.192093 -3.174484 -4.367844

C 0 2.125626 -5.296204 3.579834

C 0 -1.432114 0.680191 1.567932

C 0 -1.060272 1.476273 0.331635

C 0 -1.503311 0.757858 -0.947037

C 0 -1.321808 2.965286 0.358566

C 0 -1.298950 3.697083 1.566498

C 0 -1.643936 3.674835 -0.820236

C 0 -1.603088 5.060806 1.598251

C 0 -1.954300 5.037018 -0.789642

C 0 -1.936478 5.738525 0.420715

H 0 2.618729 -4.011185 -0.302048

H 0 1.985200 -2.557755 0.481491

H 0 2.595093 -2.486816 -1.181412

H 0 1.138489 -2.756622 -2.998260

H 0 -2.902100 -4.098709 -2.449554

H 0 -3.677643 -4.883331 -0.392609

H 0 -3.035080 -3.963486 0.975571

H 0 -2.495956 -5.620804 0.694443

H 0 1.495193 -5.554947 0.849487

H 0 -0.084671 -6.329391 1.008438

H 0 0.545715 -3.530273 5.393295

H 0 0.720490 -2.137314 4.302429

H 0 2.388351 -3.218903 2.950485

H 0 2.919678 -3.541229 4.598877

H 0 -1.327225 -5.623138 3.233032

H 0 -0.376068 -5.495255 4.724792

H 0 0.871689 -6.962952 3.017303

H 0 -0.469208 -2.482162 -4.810562

H 0 -2.196075 -2.766083 -4.525696

H 0 -1.133286 -4.113968 -4.933136

H 0 2.248642 -5.831161 4.531982

H 0 3.000748 -5.552400 2.970062

H 0 -1.237869 1.233658 2.490417

H 0 -2.528305 0.552170 1.508881

H 0 -1.117432 1.199478 -1.867233

H 0 -1.209595 -0.293182 -0.911133

H 0 -2.599380 0.793198 -0.997421

H 0 -1.062637 3.197723 2.497116

H 0 -1.676788 3.163635 -1.773895

H 0 -1.594589 5.588913 2.546402

H 0 -2.220154 5.546097 -1.710510

H 0 -2.187042 6.793900 0.446884

C 0 0.926285 1.311462 0.197906

C 0 1.357880 2.066711 -0.953979

C 0 1.978363 3.280945 -0.836533

C 0 2.307785 3.809921 0.461411

C 0 2.056046 2.981140 1.609741

C 0 1.425888 1.773056 1.469696

N 0 2.858231 5.040771 0.592789

C 0 3.223434 5.568085 1.922623

C 0 3.094437 5.896759 -0.587250

H 0 0.839890 0.235291 0.080963

H 0 1.145889 1.678375 -1.944748

H 0 2.240417 3.836136 -1.726654

H 0 2.368057 3.311142 2.591171

H 0 1.240433 1.155884 2.342148

H 0 3.990036 4.946686 2.397461

H 0 3.622681 6.574142 1.807388

H 0 2.346695 5.616302 2.576920

H 0 3.863419 5.470383 -1.240875

H 0 2.171631 6.031036 -1.159592

H 0 3.433868 6.875214 -0.252000

Int1_A-1_

Au 0 1.244427 -2.441145 -0.441608

C 0 2.096493 0.881377 1.681203

C 0 1.308054 2.262805 -0.279999

C 0 2.015789 1.820487 -2.538488

C 0 1.264437 2.594597 -1.641669

C 0 2.844788 0.456757 -0.726427

C 0 3.598375 -0.067368 -3.105058

C 0 2.092261 1.203321 0.202984

C 0 2.812451 0.751289 -2.105552

N 0 4.216352 -2.858188 -0.017617

N 0 3.703559 -0.622582 -0.268510

C 0 5.039758 -0.187791 0.278587

C 0 4.078062 -3.702764 1.197931

C 0 4.429768 -2.791369 2.401375

C 0 5.628961 -2.481606 -0.264373

C 0 5.927371 -1.365035 0.740281

C 0 3.244188 -1.893543 -0.196666

C 0 0.452058 3.773496 -2.129775

C 0 5.714628 -1.939272 2.177294

C 0 -0.722279 -3.131088 -0.802842

C 0 -1.776142 -2.140158 -1.365986

C 0 -1.267489 -1.611424 -2.727044

C 0 -3.183358 -2.773522 -1.472742

C 0 -3.725173 -3.512993 -0.402030

C 0 -3.970023 -2.647691 -2.634062

C 0 -4.993417 -4.095579 -0.484486

C 0 -5.239008 -3.233849 -2.722504

C 0 -5.759745 -3.958413 -1.647448

C 0 -1.871825 -0.819986 -0.353240

C 0 -2.900284 0.144873 -0.798088

C 0 -3.944821 0.541949 -0.025351

C 0 -4.087936 0.052391 1.328039

C 0 -3.045025 -0.796122 1.856757

C 0 -2.010320 -1.195232 1.068929

N 0 -5.152195 0.391009 2.075295

C 0 -5.314037 -0.123619 3.455417

C 0 -6.213083 1.277418 1.540882

H 0 3.022309 1.207140 2.173240

H 0 1.990159 -0.194323 1.856202

H 0 1.271956 1.392535 2.186903

H 0 0.726835 2.847016 0.428533

H 0 1.987790 2.055596 -3.598657

H 0 3.484921 0.341470 -4.112591

H 0 3.259715 -1.110117 -3.124527

H 0 4.670063 -0.080208 -2.871928

H 0 4.841810 0.513306 1.095569

H 0 5.555122 0.364991 -0.514398

H 0 4.756198 -4.555769 1.104535

H 0 3.052165 -4.073442 1.265178

H 0 3.571994 -2.126792 2.571645

H 0 4.550448 -3.390688 3.311735

H 0 5.735297 -2.150132 -1.301534

H 0 6.250118 -3.367952 -0.119289

H 0 6.968287 -1.025706 0.673559

H 0 -0.359521 4.017496 -1.436527

H 0 0.014672 3.580957 -3.115310

H 0 1.078262 4.670380 -2.225312

H 0 6.590704 -2.569301 2.386264

H 0 5.741119 -1.125719 2.912764

H 0 -1.092342 -3.627743 0.102232

H 0 -0.621437 -3.929571 -1.554303

H 0 -1.921361 -0.866344 -3.190737

H 0 -0.273970 -1.172188 -2.602984

H 0 -1.174817 -2.449464 -3.425493

H 0 -3.146634 -3.657736 0.503011

H 0 -3.595753 -2.105467 -3.493254

H 0 -5.375383 -4.670380 0.353769

H 0 -5.812819 -3.129171 -3.637983

H 0 -6.739527 -4.419303 -1.717577

H 0 -0.862224 -0.400996 -0.502913

H 0 -2.823414 0.532946 -1.807860

H 0 -4.679564 1.225529 -0.427795

H 0 -3.087608 -1.117287 2.888554

H 0 -1.230727 -1.825447 1.483325

H 0 -4.529850 0.265165 4.112591

H 0 -6.279022 0.198860 3.840848

H 0 -5.286255 -1.216556 3.465592

H 0 -5.803081 2.253876 1.267056

H 0 -6.691058 0.826474 0.666241

H 0 -6.968287 1.426317 2.309724

TS2_A-1_

Au 0 -0.589845 0.037445 -0.386798

C 0 -3.869742 -0.927546 -2.381429

C 0 -4.671014 1.396200 -1.820741

C 0 -4.585290 2.299888 0.410004

C 0 -4.924778 2.459025 -0.942920

C 0 -3.783290 0.064639 -0.017162

C 0 -3.656915 0.988832 2.361092

C 0 -4.107993 0.186685 -1.384935

C 0 -4.016290 1.116024 0.896674

N 0 -1.416268 -2.388369 1.300247

N 0 -3.207201 -1.173763 0.489010

C 0 -4.174307 -2.303573 0.737954

C 0 -0.569418 -3.376439 0.579349

C 0 -1.515980 -4.140423 -0.381718

C 0 -2.384923 -3.050662 2.209320

C 0 -3.496321 -3.576083 1.294491

C 0 -1.871646 -1.323501 0.560488

C 0 -5.567656 3.736118 -1.433220

C 0 -2.854288 -4.580534 0.284592

C 0 0.348951 1.669357 -1.529701

C 0 1.647967 2.324801 -0.980752

C 0 2.574288 1.100079 -0.677539

C 0 3.130204 0.345719 -1.756256

C 0 4.039813 -0.676011 -1.557193

C 0 4.503444 -1.001607 -0.247696

C 0 3.980814 -0.238570 0.835798

C 0 3.075984 0.787333 0.620668

N 0 5.420602 -2.002041 -0.043879

C 0 5.914209 -2.293607 1.308756

C 0 5.963267 -2.758529 -1.180541

C 0 2.235076 3.246964 -2.093015

C 0 1.406641 3.185385 0.275759

C 0 0.140597 3.356883 0.860282

C 0 2.492628 3.879150 0.850647

C 0 -0.034565 4.185898 1.978160

C 0 2.321499 4.703938 1.964344

C 0 1.051916 4.862707 2.534975

H 0 1.124647 0.393577 -0.586417

H 0 -4.683323 -1.665273 -2.369891

H 0 -2.934136 -1.461790 -2.188485

H 0 -3.820539 -0.526089 -3.397371

H 0 -4.916866 1.506384 -2.872883

H 0 -4.769917 3.114931 1.103705

H 0 -3.971382 1.879497 2.911142

H 0 -2.576217 0.871096 2.507358

H 0 -4.143313 0.124555 2.829718

H 0 -4.685547 -2.497524 -0.209928

H 0 -4.920723 -1.941979 1.452715

H 0 -0.102215 -4.031430 1.319704

H 0 0.215855 -2.849619 0.030449

H 0 -1.726188 -3.480373 -1.234167

H 0 -1.010425 -5.024331 -0.787919

H 0 -2.750400 -2.317612 2.934173

H 0 -1.861933 -3.842271 2.749347

H 0 -4.259681 -4.122758 1.860622

H 0 -5.404845 3.882017 -2.505270

H 0 -5.173824 4.611566 -0.906784

H 0 -6.652770 3.718024 -1.266263

H 0 -2.674654 -5.506590 0.848137

H 0 -3.577841 -4.835977 -0.498766

H 0 0.543686 1.216728 -2.507091

H 0 -0.499863 2.357078 -1.621486

H 0 2.827476 0.567290 -2.774370

H 0 4.415203 -1.214765 -2.417007

H 0 4.303794 -0.440320 1.848403

H 0 2.723656 1.355522 1.472621

H 0 5.097436 -2.593651 1.976829

H 0 6.628915 -3.114462 1.258109

H 0 6.421289 -1.425681 1.748085

H 0 5.167516 -3.268219 -1.737378

H 0 6.511635 -2.108679 -1.873939

H 0 6.652770 -3.515518 -0.808103

H 0 3.184661 3.693367 -1.787288

H 0 2.401727 2.696535 -3.024093

H 0 1.532906 4.060782 -2.301077

H 0 -0.727656 2.854901 0.451433

H 0 3.486582 3.766713 0.428825

H 0 -1.025419 4.303397 2.405642

H 0 3.176087 5.224912 2.383863

H 0 0.915527 5.506590 3.397371

Pro_A-1_

Au 0 0.877180 -1.185969 -0.163206

C 0 4.053700 0.242519 1.846365

C 0 2.841486 2.449237 1.973756

C 0 1.793026 3.468877 0.061063

C 0 2.114992 3.515675 1.426233

C 0 2.919024 1.348321 -0.169140

C 0 1.813901 2.390898 -2.223434

C 0 3.261209 1.357581 1.198649

C 0 2.184123 2.400537 -0.756338

N 0 2.930762 -1.628170 -2.300717

N 0 3.353419 0.250406 -1.021509

C 0 4.793096 0.308844 -1.475425

C 0 3.195782 -3.055166 -1.970456

C 0 4.525849 -3.075687 -1.175823

C 0 3.923785 -1.092568 -3.265364

C 0 5.185257 -0.837839 -2.434202

C 0 2.561599 -0.810893 -1.260135

C 0 1.709240 4.697740 2.276548

C 0 5.641169 -2.198016 -1.817732

C 0 -4.472058 -2.086941 -2.474899

C 0 -3.430557 -1.100138 -1.876324

C 0 -2.587232 -1.758581 -0.760314

C 0 -1.847803 -0.975614 0.124742

C 0 -0.985372 -1.539462 1.124562

C 0 -0.986358 -2.971762 1.332697

C 0 -1.719251 -3.755371 0.399633

C 0 -2.484226 -3.160471 -0.596328

N 0 -0.326089 -3.539330 2.389938

C 0 0.461163 -2.704385 3.301048

C 0 -0.308458 -4.999404 2.560793

C 0 -4.202103 0.111885 -1.301897

C 0 -4.143629 1.392767 -1.874524

C 0 -5.040011 -0.074958 -0.183783

C 0 -4.897964 2.451968 -1.349599

C 0 -5.792948 0.977820 0.340643

C 0 -5.725053 2.250056 -0.242353

C 0 -2.458419 -0.693799 -3.019249

H 0 5.135620 0.385381 1.721484

H 0 3.797854 -0.738167 1.433201

H 0 3.862576 0.214216 2.923022

H 0 3.091404 2.464247 3.030788

H 0 1.225851 4.283429 -0.379599

H 0 1.315738 3.324047 -2.498438

H 0 1.133468 1.566113 -2.468257

H 0 2.694907 2.285592 -2.868586

H 0 5.410079 0.311996 -0.571787

H 0 4.930713 1.271703 -1.977529

H 0 3.255480 -3.616098 -2.906879

H 0 2.367472 -3.450756 -1.376548

H 0 4.310703 -2.716368 -0.160079

H 0 4.891039 -4.104371 -1.074901

H 0 3.526598 -0.178649 -3.715910

H 0 4.069343 -1.834220 -4.052870

H 0 6.008493 -0.466771 -3.056175

H 0 1.613852 4.420504 3.330845

H 0 0.754278 5.119578 1.947471

H 0 2.456939 5.499659 2.216108

H 0 6.104335 -2.766007 -2.636445

H 0 6.433502 -2.027032 -1.079302

H 0 -3.985912 -2.920559 -2.994511

H 0 -5.137004 -2.493110 -1.706107

H 0 -5.091005 -1.557949 -3.205637

H 0 -1.936901 0.105299 0.089557

H 0 -0.705493 -0.892469 1.951883

H 0 -1.726243 -4.833824 0.484053

H 0 -3.039562 -3.815428 -1.257383

H 0 1.249963 -2.161912 2.757819

H 0 -0.165893 -1.969073 3.819690

H 0 0.930914 -3.337590 4.052870

H 0 -1.325422 -5.400093 2.632800

H 0 0.206769 -5.499659 1.730205

H 0 0.214479 -5.242308 3.485239

H 0 -3.517166 1.580029 -2.738407

H 0 -5.100129 -1.054260 0.281864

H 0 -4.838396 3.431836 -1.812863

H 0 -6.433502 0.806476 1.199984

H 0 -6.311219 3.069030 0.161192

H 0 -1.727760 0.049750 -2.682657

H 0 -1.908257 -1.575426 -3.365368

H 0 -3.005272 -0.283556 -3.874912

Int1_A-1’_

Au 0 1.053772 -1.941162 -1.166489

C 0 0.035202 -4.925583 1.310013

C 0 2.227966 -4.581894 2.510925

C 0 4.319672 -4.355300 1.341263

C 0 3.616394 -4.393845 2.554260

C 0 2.280182 -4.716995 0.103958

C 0 4.465942 -4.437317 -1.180130

C 0 1.537582 -4.748657 1.301376

C 0 3.675583 -4.513748 0.106857

N 0 0.776077 -4.208038 -3.197372

N 0 1.613388 -4.934143 -1.171677

C 0 1.366455 -6.381210 -1.513245

C 0 -0.643509 -3.961929 -3.552322

C 0 -1.475891 -5.040817 -2.813232

C 0 1.268860 -5.484879 -3.768890

C 0 0.676880 -6.582657 -2.879791

C 0 1.129807 -3.899139 -1.893570

C 0 4.341827 -4.254500 3.873703

C 0 -0.879227 -6.473373 -2.945419

C 0 1.259750 0.047302 -0.476578

C 0 0.328613 0.661453 0.619812

C 0 -1.018921 1.262955 -0.081482

C 0 -1.741700 0.267487 -0.909988

C 0 -3.065582 -0.007538 -0.768555

C 0 -3.869370 0.692810 0.208771

C 0 -3.224625 1.686400 1.035156

C 0 -1.901428 1.954224 0.892288

N 0 -5.180878 0.431900 0.342270

C 0 -5.844604 -0.606140 -0.478012

C 0 -6.019867 1.206772 1.286469

C 0 1.049942 1.854431 1.295197

C 0 1.551147 2.910600 0.505630

C 0 1.266708 1.922211 2.683441

C 0 2.235809 3.986572 1.076600

C 0 1.956558 2.998428 3.259277

C 0 2.441345 4.036865 2.461349

C 0 -0.096130 -0.411850 1.643631

H 0 -0.265549 -5.954239 1.070374

H 0 -0.447037 -4.261368 0.584518

H 0 -0.369141 -4.697037 2.300262

H 0 1.664642 -4.597321 3.439758

H 0 5.393921 -4.195175 1.353210

H 0 5.535873 -4.357254 -0.970428

H 0 4.174881 -3.562607 -1.774719

H 0 4.316971 -5.322342 -1.810913

H 0 0.777115 -6.808090 -0.695557

H 0 2.338120 -6.886688 -1.518127

H 0 -0.745834 -4.027710 -4.639755

H 0 -0.921570 -2.954651 -3.231461

H 0 -1.514130 -4.756241 -1.753067

H 0 -2.509811 -5.047089 -3.180389

H 0 2.362640 -5.482361 -3.755035

H 0 0.936508 -5.547363 -4.807510

H 0 0.950577 -7.584106 -3.233795

H 0 3.671051 -3.906418 4.665512

H 0 5.175568 -3.548462 3.799973

H 0 4.759323 -5.216980 4.198364

H 0 -1.175949 -6.886520 -3.919907

H 0 -1.331360 -7.123947 -2.186569

H 0 1.295685 0.720078 -1.347092

H 0 2.276489 0.059769 -0.056198

H 0 -0.615845 2.033279 -0.764923

H 0 -1.158386 -0.265137 -1.653229

H 0 -3.528473 -0.744629 -1.410782

H 0 -3.805450 2.240891 1.757660

H 0 -1.441208 2.708298 1.521255

H 0 -6.876648 -0.708160 -0.148514

H 0 -5.842422 -0.329376 -1.537277

H 0 -5.347931 -1.572556 -0.356094

H 0 -5.706619 1.032440 2.320343

H 0 -5.954834 2.275269 1.059189

H 0 -7.054993 0.888184 1.180161

H 0 1.429886 2.880814 -0.572891

H 0 0.913681 1.129837 3.331467

H 0 2.620941 4.778061 0.441100

H 0 2.115051 3.017624 4.332900

H 0 2.977112 4.868546 2.906600

H 0 -0.649384 -1.206924 1.137756

H 0 0.791306 -0.872345 2.089218

H 0 -0.721527 -0.007370 2.448181

C 0 -5.193130 4.683716 -0.252243

C 0 -4.082214 5.324432 0.312149

C 0 -2.835495 5.289078 -0.313812

C 0 -2.657349 4.602249 -1.543411

C 0 -3.788788 3.958832 -2.107300

C 0 -5.028671 4.004486 -1.467651

N 0 -1.413452 4.570679 -2.178162

C 0 -0.348267 5.470520 -1.716934

C 0 -1.308441 4.014893 -3.531036

H 0 -6.170212 4.757584 0.214554

H 0 -4.188721 5.878555 1.240005

H 0 -2.006866 5.815063 0.142227

H 0 -3.709412 3.448639 -3.058609

H 0 -5.882523 3.529816 -1.943390

H 0 -0.056900 5.243820 -0.685760

H 0 0.531052 5.331680 -2.347778

H 0 -0.645645 6.528183 -1.768478

H 0 -1.622971 2.964783 -3.550186

H 0 -1.917206 4.566910 -4.263184

H 0 -0.266830 4.055038 -3.852509

TS2_A-1’_

Au 0 1.599991 -0.710388 -1.273178

C 0 3.483780 -1.113235 2.264404

C 0 4.260849 1.276000 2.025223

C 0 5.222260 2.124283 -0.012131

C 0 4.821167 2.345779 1.313660

C 0 4.533844 -0.183929 0.125977

C 0 5.509369 0.679900 -2.066132

C 0 4.109772 0.002548 1.456818

C 0 5.088943 0.871796 -0.626144

N 0 3.473358 -3.025436 -1.931213

N 0 4.436295 -1.499481 -0.489426

C 0 5.560898 -2.440292 -0.140717

C 0 2.542908 -4.128600 -1.583786

C 0 3.002121 -4.681061 -0.210144

C 0 4.844100 -3.528442 -2.190903

C 0 5.436447 -3.825729 -0.810562

C 0 3.335938 -1.866898 -1.182236

C 0 5.006607 3.699142 1.962341

C 0 4.541214 -4.902313 -0.119797

C 0 -0.022232 0.616180 -1.586243

C 0 -0.925491 1.158295 -0.432602

C 0 -2.028244 0.083923 -0.001358

C 0 -1.545258 -1.247894 0.313431

C 0 -2.149124 -2.081406 1.226486

C 0 -3.278946 -1.646622 1.985153

C 0 -3.734543 -0.305145 1.773651

C 0 -3.114029 0.517197 0.862671

N 0 -3.896286 -2.470978 2.878098

C 0 -3.443817 -3.861740 3.048782

C 0 -5.023483 -1.988861 3.694595

C 0 -1.620575 2.491623 -0.852600

C 0 -1.806046 2.832840 -2.207962

C 0 -2.065964 3.431274 0.102646

C 0 -2.430023 4.027252 -2.591003

C 0 -2.688232 4.627853 -0.273590

C 0 -2.882370 4.930542 -1.624466

C 0 -0.035187 1.420044 0.818375

H 0 4.225128 -1.852371 2.596436

H 0 2.721008 -1.645508 1.686035

H 0 3.008011 -0.712036 3.163864

H 0 3.929200 1.433640 3.047638

H 0 5.645691 2.944565 -0.584595

H 0 5.976334 1.588791 -2.454865

H 0 4.649139 0.445297 -2.704971

H 0 6.231049 -0.138321 -2.180756

H 0 5.587036 -2.518646 0.950745

H 0 6.495056 -1.965439 -0.459732

H 0 2.591812 -4.883911 -2.374069

H 0 1.524765 -3.734009 -1.532928

H 0 2.688995 -3.959610 0.556534

H 0 2.489777 -5.624924 0.013870

H 0 5.408493 -2.761871 -2.729843

H 0 4.769806 -4.413940 -2.826126

H 0 6.449722 -4.239441 -0.883514

H 0 4.319778 3.838074 2.803085

H 0 4.838287 4.512238 1.248383

H 0 6.027283 3.814468 2.350876

H 0 4.785202 -5.860077 -0.601074

H 0 4.829376 -5.007400 0.933502

H 0 -0.645767 0.209167 -2.393112

H 0 0.496628 1.485458 -2.017273

H 0 -0.662476 -1.592575 -0.215439

H 0 -1.748322 -3.075027 1.375870

H 0 -4.564224 0.080917 2.351349

H 0 -3.475754 1.533661 0.757660

H 0 -4.111603 -4.369919 3.743149

H 0 -3.467682 -4.398392 2.093597

H 0 -2.426697 -3.906952 3.455322

H 0 -4.725052 -1.145676 4.327576

H 0 -5.865585 -1.676376 3.066300

H 0 -5.363403 -2.795776 4.342300

H 0 -1.415390 2.181519 -2.978851

H 0 -1.913986 3.247162 1.159653

H 0 -2.541290 4.258606 -3.646088

H 0 -3.009644 5.326874 0.492371

H 0 -3.358536 5.860077 -1.918182

H 0 0.469971 0.494843 1.107193

H 0 0.733994 2.161560 0.578354

H 0 -0.606903 1.777588 1.680923

H 0 -2.737839 -0.188431 -1.136734

C 0 -4.517227 -4.673187 -1.482803

C 0 -5.511185 -3.713394 -1.269470

C 0 -5.258347 -2.358368 -1.513885

C 0 -3.996338 -1.951843 -1.972519

C 0 -2.996780 -2.917130 -2.188019

C 0 -3.260300 -4.267288 -1.946808

N 0 -3.673828 -0.541946 -2.209137

C 0 -4.791580 0.419708 -1.975861

C 0 -3.080353 -0.307907 -3.570084

H 0 -4.723100 -5.723175 -1.305405

H 0 -6.495071 -4.015305 -0.926025

H 0 -6.052933 -1.642014 -1.352844

H 0 -2.012619 -2.622009 -2.532089

H 0 -2.482025 -5.001846 -2.125214

H 0 -5.150497 0.326813 -0.950974

H 0 -4.402878 1.428406 -2.122330

H 0 -5.617218 0.242065 -2.674133

H 0 -2.158691 -0.873108 -3.688339

H 0 -3.795639 -0.612595 -4.342300

H 0 -2.858383 0.752487 -3.671768

Int2_A-1’_

Au 0 1.887848 -2.544922 0.454498

C 0 3.577515 -4.263657 3.532272

C 0 5.865295 -3.816040 2.565353

C 0 6.417694 -4.305756 0.274384

C 0 6.806961 -3.809937 1.526962

C 0 4.215714 -4.815353 1.118607

C 0 4.742493 -5.314026 -1.326141

C 0 4.566071 -4.313324 2.388489

C 0 5.131104 -4.812637 0.046890

N 0 0.738022 -5.342819 0.087128

N 0 2.897781 -5.394012 0.903214

C 0 2.765961 -6.827713 1.348770

C 0 -0.507600 -5.067719 0.848206

C 0 -0.315018 -5.699432 2.249939

C 0 0.895432 -6.788620 -0.198105

C 0 1.356964 -7.416550 1.120560

C 0 1.855194 -4.632889 0.501312

C 0 8.211975 -3.299515 1.755905

C 0 0.259323 -7.145874 2.198395

C 0 2.003098 -0.440460 0.344803

C 0 0.837128 0.410614 0.949768

C 0 -0.522552 0.148514 0.252838

C 0 -0.617508 -0.423523 -1.032150

C 0 -1.839386 -0.651810 -1.666992

C 0 -3.069046 -0.334442 -1.036407

C 0 -2.988312 0.256700 0.255447

C 0 -1.741638 0.479172 0.870026

N 0 -4.290649 -0.600052 -1.643906

C 0 -4.329147 -1.283249 -2.939896

C 0 -5.534439 -0.418640 -0.895767

C 0 1.190079 1.916977 0.757935

C 0 1.384796 2.427948 -0.545349

C 0 1.368927 2.809800 1.831268

C 0 1.748000 3.760071 -0.765579

C 0 1.730057 4.149445 1.614900

C 0 1.921600 4.633224 0.317871

C 0 0.728516 0.049927 2.453568

H 0 3.284332 -5.263153 3.878662

H 0 2.664063 -3.733551 3.239578

H 0 4.011475 -3.739716 4.388504

H 0 6.143265 -3.418427 3.537308

H 0 7.128494 -4.295685 -0.547058

H 0 5.598541 -5.276306 -2.005402

H 0 3.941498 -4.704285 -1.761078

H 0 4.384232 -6.350861 -1.302811

H 0 3.053940 -6.862976 2.404282

H 0 3.500504 -7.415344 0.787308

H 0 -1.349045 -5.508209 0.304031

H 0 -0.654419 -3.986786 0.915588

H 0 0.370514 -5.050385 2.810394

H 0 -1.263840 -5.708328 2.800476

H 0 1.630997 -6.916794 -0.997864

H 0 -0.065216 -7.176468 -0.544952

H 0 1.472488 -8.504166 1.035126

H 0 8.241745 -2.554947 2.557755

H 0 8.623611 -2.840347 0.851227

H 0 8.887970 -4.116287 2.042984

H 0 -0.563889 -7.841095 1.979340

H 0 0.636215 -7.420563 3.191422

H 0 2.169922 -0.165939 -0.706421

H 0 2.920029 -0.140610 0.872879

H 0 0.286423 -0.734772 -1.540436

H 0 -1.833282 -1.121979 -2.642136

H 0 -3.890442 0.426331 0.833588

H 0 -1.742935 0.888900 1.875427

H 0 -5.366333 -1.377075 -3.263397

H 0 -3.789185 -0.714569 -3.707077

H 0 -3.891235 -2.290543 -2.892044

H 0 -5.558441 -1.024673 0.021545

H 0 -5.686859 0.632492 -0.614380

H 0 -6.376205 -0.718155 -1.520767

H 0 1.283920 1.760666 -1.395813

H 0 1.249710 2.465927 2.851227

H 0 1.917160 4.111893 -1.779434

H 0 1.875168 4.806763 2.467010

H 0 2.218704 5.663788 0.152542

H 0 0.424438 -0.995667 2.548798

H 0 1.706300 0.156876 2.934540

H 0 0.013519 0.670242 3.005463

C 0 -6.633362 5.088776 -0.846115

C 0 -6.338486 4.108246 0.108353

C 0 -5.062775 3.536316 0.156097

C 0 -4.093948 3.952911 -0.761795

C 0 -4.369507 4.934677 -1.714798

C 0 -5.650223 5.499512 -1.752792

N 0 -2.728714 3.351822 -0.649887

C 0 -1.856445 4.132019 0.330750

C 0 -2.014267 3.158142 -1.975494

H 0 -7.622314 5.531357 -0.881516

H 0 -7.095245 3.789000 0.815857

H 0 -4.830811 2.781906 0.900772

H 0 -3.619919 5.265381 -2.423279

H 0 -5.873886 6.260727 -2.491455

H 0 -2.835526 2.386948 -0.261444

H 0 -2.396118 4.216309 1.273605

H 0 -0.918182 3.595978 0.469345

H 0 -1.676620 5.122818 -0.086136

H 0 -2.675095 2.614670 -2.649094

H 0 -1.749069 4.129822 -2.389389

H 0 -1.110947 2.582993 -1.778458

TS3_A-1’_

Au 0 1.665985 0.267746 0.354996

C 0 4.438736 -0.425613 -2.357254

C 0 5.049225 -2.538467 -1.122772

C 0 5.416718 -2.593430 1.256256

C 0 5.374725 -3.270355 0.028076

C 0 4.833130 -0.518692 0.173370

C 0 5.211624 -0.528702 2.698547

C 0 4.780319 -1.162445 -1.080368

C 0 5.153275 -1.220642 1.354095

N 0 3.292816 2.744370 0.831787

N 0 4.591812 0.915482 0.265350

C 0 5.776581 1.785843 -0.080948

C 0 2.507172 3.621140 -0.082535

C 0 3.330276 3.743805 -1.388474

C 0 4.552841 3.399918 1.266098

C 0 5.497009 3.297729 0.063660

C 0 3.360580 1.410309 0.488785

C 0 5.696000 -4.745026 -0.056412

C 0 4.836823 4.042709 -1.138916

C 0 -0.105896 -1.125961 -0.032822

C 0 -0.957932 -0.703522 -1.298737

C 0 -2.022751 0.379410 -0.983871

C 0 -1.667542 1.569168 -0.322662

C 0 -2.575439 2.604568 -0.091751

C 0 -3.918823 2.506409 -0.537400

C 0 -4.276000 1.321243 -1.226715

C 0 -3.349686 0.300797 -1.444061

N 0 -4.835739 3.524094 -0.315933

C 0 -4.425873 4.745102 0.375046

C 0 -6.203430 3.402435 -0.822540

C 0 -1.612534 -2.018082 -1.806610

C 0 -1.297440 -2.591995 -3.049789

C 0 -2.530014 -2.707000 -0.984131

C 0 -1.869827 -3.806015 -3.456055

C 0 -3.102432 -3.915741 -1.386368

C 0 -2.772568 -4.474976 -2.627548

C 0 -0.021210 -0.105865 -2.386688

H 0 5.287796 0.160324 -2.734070

H 0 3.596100 0.260071 -2.221924

H 0 4.168488 -1.134232 -3.144867

H 0 5.001816 -3.048691 -2.080414

H 0 5.663055 -3.144500 2.159164

H 0 5.480072 -1.239563 3.484390

H 0 4.248932 -0.078186 2.969147

H 0 5.958969 0.274719 2.715485

H 0 6.073090 1.523407 -1.100952

H 0 6.593521 1.496429 0.587769

H 0 2.360367 4.584366 0.413986

H 0 1.529160 3.170639 -0.270355

H 0 3.226929 2.797943 -1.937119

H 0 2.907791 4.526276 -2.029449

H 0 4.940521 2.878860 2.146332

H 0 4.322342 4.429886 1.545166

H 0 6.458893 3.783859 0.265457

H 0 5.438309 -5.266052 0.870941

H 0 6.768173 -4.904100 -0.232513

H 0 5.156876 -5.226440 -0.878265

H 0 4.952438 5.116806 -0.937469

H 0 5.401062 3.842133 -2.057510

H 0 -0.733521 -1.804291 0.565079

H 0 0.678100 -1.824478 -0.373337

H 0 -0.647385 1.702271 0.027084

H 0 -2.233643 3.497711 0.417389

H 0 -5.280396 1.195374 -1.610077

H 0 -3.677933 -0.575851 -1.988464

H 0 -4.060837 4.534576 1.390305

H 0 -3.632538 5.279100 -0.167755

H 0 -5.282990 5.413834 0.460358

H 0 -6.717728 2.530670 -0.395676

H 0 -6.227905 3.309738 -1.917130

H 0 -6.768173 4.293610 -0.546677

H 0 -0.604294 -2.102478 -3.722046

H 0 -2.817963 -2.283859 -0.026917

H 0 -1.606644 -4.222458 -4.423355

H 0 -3.808502 -4.418640 -0.732834

H 0 -3.216263 -5.413834 -2.942123

H 0 -0.578812 0.093979 -3.307205

H 0 0.393921 0.847244 -2.046158

H 0 0.812363 -0.779388 -2.617813

H 0 -0.172577 -0.487700 1.275513

C 0 -4.324295 -2.327988 3.206100

C 0 -4.223282 -1.135208 2.479996

C 0 -2.987076 -0.505188 2.314728

C 0 -1.829941 -1.064331 2.884308

C 0 -1.929382 -2.261642 3.610336

C 0 -3.173187 -2.887009 3.765625

N 0 -0.536240 -0.391418 2.676926

C 0 -0.555450 1.060043 3.055420

C 0 0.619751 -1.068298 3.335632

H 0 -5.285858 -2.813156 3.332321

H 0 -5.104935 -0.691330 2.030777

H 0 -2.931686 0.402267 1.727737

H 0 -1.061707 -2.718048 4.066971

H 0 -3.234039 -3.810211 4.331863

H 0 0.389877 1.509171 2.738785

H 0 -1.372665 1.567719 2.548264

H 0 -0.671402 1.168289 4.139709

H 0 0.494812 -1.099182 4.423340

H 0 0.728104 -2.082642 2.947540

H 0 1.523300 -0.506424 3.093430

Pro_A-1’_

Au 0 0.600342 -2.194580 0.493439

C 0 2.825882 -4.299973 3.073196

C 0 4.600327 -2.522781 2.846481

C 0 5.321060 -1.671158 0.712814

C 0 5.428131 -1.662231 2.111938

C 0 3.616943 -3.374771 0.817764

C 0 4.339691 -2.492203 -1.466476

C 0 3.690582 -3.391922 2.225784

C 0 4.426926 -2.516953 0.043686

N 0 0.816528 -4.704834 -1.132126

N 0 2.710571 -4.279907 0.124512

C 0 3.196609 -5.702545 -0.013580

C 0 -0.475876 -5.291138 -0.682510

C 0 -0.120010 -6.356079 0.384827

C 0 1.636124 -5.690704 -1.881165

C 0 2.231888 -6.609695 -0.809067

C 0 1.470779 -3.897079 -0.232071

C 0 6.426025 -0.763702 2.806000

C 0 1.052673 -7.287735 -0.042038

C 0 -1.333588 2.338181 -2.824142

C 0 -0.194733 1.600632 -2.063385

C 0 -0.726913 0.849258 -0.821640

C 0 0.126022 0.502700 0.222397

C 0 -0.304611 -0.281265 1.348251

C 0 -1.709595 -0.601089 1.488754

C 0 -2.559143 -0.270889 0.395905

C 0 -2.075775 0.427490 -0.702362

N 0 -2.196243 -1.182175 2.628616

C 0 -1.282639 -1.568420 3.705582

C 0 -3.615891 -1.555847 2.726547

C 0 0.846497 2.662079 -1.637512

C 0 2.136688 2.727570 -2.188187

C 0 0.481659 3.642441 -0.692139

C 0 3.032272 3.738174 -1.809677

C 0 1.371231 4.650146 -0.313446

C 0 2.654678 4.702881 -0.872940

C 0 0.404251 0.539200 -3.028076

H 0 3.235504 -5.317245 3.135132

H 0 1.805908 -4.375519 2.682678

H 0 2.765976 -3.921722 4.097656

H 0 4.661057 -2.520660 3.931015

H 0 5.947769 -1.005066 0.127228

H 0 5.075836 -1.799118 -1.881714

H 0 3.348907 -2.173111 -1.812012

H 0 4.533325 -3.479050 -1.905258

H 0 3.364990 -6.079590 0.999619

H 0 4.166092 -5.662231 -0.520233

H 0 -0.976974 -5.720215 -1.554276

H 0 -1.107956 -4.501770 -0.266876

H 0 0.148285 -5.823700 1.307526

H 0 -0.999237 -6.967896 0.618271

H 0 2.403336 -5.157745 -2.449921

H 0 0.983704 -6.214218 -2.582474

H 0 2.837357 -7.407944 -1.254500

H 0 6.110168 -0.525208 3.826233

H 0 6.563843 0.177124 2.263626

H 0 7.409546 -1.247452 2.873138

H 0 0.656128 -8.063751 -0.711380

H 0 1.434845 -7.813004 0.841431

H 0 -2.054138 1.633957 -3.253998

H 0 -1.872498 3.031006 -2.170166

H 0 -0.902298 2.918518 -3.645630

H 0 1.154572 0.847946 0.216934

H 0 0.307129 -0.222855 2.245270

H 0 -3.611389 -0.520950 0.422592

H 0 -2.788589 0.668732 -1.483154

H 0 -0.525436 -2.283081 3.346100

H 0 -0.759644 -0.699707 4.123764

H 0 -1.850845 -2.038666 4.507477

H 0 -4.265549 -0.699188 2.524994

H 0 -3.876144 -2.353500 2.018326

H 0 -3.819244 -1.908310 3.737579

H 0 2.457382 1.997635 -2.921783

H 0 -0.507721 3.612259 -0.245316

H 0 4.022430 3.769104 -2.253616

H 0 1.063751 5.394684 0.414139

H 0 3.346603 5.486649 -0.582900

H 0 1.248856 0.012131 -2.571045

H 0 -0.360825 -0.204071 -3.277161

H 0 0.742310 0.999298 -3.962997

C 0 -6.838333 -0.409912 0.973572

C 0 -6.644318 0.956711 0.734650

C 0 -6.178955 1.414200 -0.499390

C 0 -5.885315 0.505524 -1.547700

C 0 -6.089493 -0.875793 -1.297562

C 0 -6.557388 -1.315964 -0.056839

N 0 -5.410706 0.955414 -2.784836

C 0 -5.424927 2.391205 -3.082611

C 0 -5.327362 0.017776 -3.908051

H 0 -7.227646 -0.755814 1.925247

H 0 -6.872543 1.681061 1.510818

H 0 -6.059052 2.479279 -0.649323

H 0 -5.905487 -1.605530 -2.075485

H 0 -6.723740 -2.378800 0.094467

H 0 -4.800507 2.947189 -2.374039

H 0 -5.015503 2.551376 -4.081085

H 0 -6.438904 2.817841 -3.052002

H 0 -4.649078 -0.812851 -3.679596

H 0 -6.306580 -0.406876 -4.177400

H 0 -4.930054 0.540543 -4.779434

Rea_A-2_

Au 0 -0.806213 0.219055 -1.426651

C 0 -3.919708 -1.808304 -2.888916

C 0 -5.173889 -1.210526 -0.785980

C 0 -4.224686 -1.464462 1.412262

C 0 -5.310364 -1.093338 0.604111

C 0 -2.934280 -2.064835 -0.534729

C 0 -1.883179 -2.340912 1.777191

C 0 -4.000824 -1.698288 -1.381546

C 0 -3.029770 -1.953979 0.868805

N 0 0.536682 -2.452438 -1.642334

N 0 -1.707291 -2.606506 -1.103073

C 0 -1.767120 -4.060593 -1.507202

C 0 1.150177 -2.206268 -2.977188

C 0 0.259155 -2.945053 -4.006821

C 0 0.645859 -3.877975 -1.242400

C 0 -0.429779 -4.603378 -2.057571

C 0 -0.655029 -1.818497 -1.386887

C 0 -6.601135 -0.602493 1.218964

C 0 -0.113861 -4.393295 -3.572600

C 0 1.331116 3.522018 -0.307343

C 0 1.223938 3.324554 -1.719833

C 0 0.054443 2.848740 -2.283554

C 0 -1.076050 2.491165 -1.480500

C 0 -0.976410 2.741623 -0.073441

C 0 0.190613 3.214386 0.498535

N 0 2.481155 4.004471 0.252579

C 0 3.636337 4.351303 -0.590363

C 0 2.571350 4.226822 1.704453

H 0 -4.172821 -2.818253 -3.238861

H 0 -2.922272 -1.564484 -3.267303

H 0 -4.631042 -1.123993 -3.359787

H 0 -5.999405 -0.916138 -1.427460

H 0 -4.309662 -1.374481 2.491257

H 0 -2.172180 -2.231979 2.825668

H 0 -0.998856 -1.714294 1.608444

H 0 -1.574677 -3.383209 1.628357

H 0 -2.574463 -4.151398 -2.239929

H 0 -2.057846 -4.628143 -0.617661

H 0 2.175110 -2.586000 -2.955841

H 0 1.178009 -1.130829 -3.172012

H 0 -0.654739 -2.350388 -4.139740

H 0 0.756943 -2.978577 -4.982803

H 0 0.480515 -3.959396 -0.164368

H 0 1.658279 -4.218719 -1.466600

H 0 -0.419632 -5.682831 -1.865585

H 0 -7.146225 0.056458 0.536102

H 0 -6.420197 -0.054764 2.149323

H 0 -7.265030 -1.442993 1.460785

H 0 0.731308 -5.056015 -3.804900

H 0 -0.954285 -4.746140 -4.182114

H 0 2.054413 3.571823 -2.366760

H 0 -0.016891 2.758972 -3.363037

H 0 -2.061874 2.455017 -1.941833

H 0 -1.844589 2.569092 0.554749

H 0 0.221695 3.375854 1.567261

H 0 3.990494 3.482132 -1.156891

H 0 4.450577 4.694214 0.046524

H 0 3.392609 5.155167 -1.295135

H 0 1.851608 4.982590 2.041168

H 0 3.572632 4.579178 1.948257

H 0 2.392761 3.298691 2.259644

TS1_A-2_

Au 0 -0.191589 -0.501816 -0.607071

C 0 -2.714310 2.400635 0.382904

C 0 -4.073990 0.719238 1.683685

C 0 -4.698044 -1.421600 0.776230

C 0 -4.727112 -0.510498 1.842789

C 0 -3.416168 0.126129 -0.557770

C 0 -4.026718 -2.144318 -1.550003

C 0 -3.414581 1.063782 0.494415

C 0 -4.051147 -1.127000 -0.430862

N 0 -1.098236 0.243896 -3.392883

N 0 -2.784256 0.455704 -1.827591

C 0 -3.622116 1.304977 -2.750961

C 0 0.010071 1.196487 -3.668121

C 0 -0.576553 2.618622 -3.486038

C 0 -2.159286 0.325806 -4.426559

C 0 -2.941330 1.602890 -4.104797

C 0 -1.495438 0.143906 -2.073090

C 0 -5.460876 -0.842194 3.122482

C 0 -1.954468 2.809784 -4.185181

C 0 1.199738 -2.738571 3.461670

C 0 1.252670 -3.413605 2.209015

C 0 1.168350 -2.706497 1.016327

C 0 1.038620 -1.282806 0.952530

C 0 0.975113 -0.639511 2.227753

C 0 1.057297 -1.321671 3.433975

N 0 1.285583 -3.426956 4.651123

C 0 1.408859 -4.889206 4.655121

C 0 1.206009 -2.712524 5.930267

H 0 -3.273209 3.111725 -0.239929

H 0 -1.713211 2.296783 -0.049210

H 0 -2.608719 2.857864 1.370682

H 0 -4.073044 1.430237 2.504898

H 0 -5.188370 -2.384689 0.884811

H 0 -4.618210 -3.023727 -1.282318

H 0 -3.005234 -2.481110 -1.764542

H 0 -4.438324 -1.741516 -2.483749

H 0 -3.863541 2.223541 -2.207458

H 0 -4.559616 0.766220 -2.924179

H 0 0.369263 1.019333 -4.686249

H 0 0.827576 1.014969 -2.966583

H 0 -0.681580 2.796021 -2.407440

H 0 0.127594 3.370667 -3.861298

H 0 -2.783157 -0.570892 -4.368652

H 0 -1.680054 0.351395 -5.407547

H 0 -3.740814 1.781891 -4.833847

H 0 -5.095505 -0.241684 3.961136

H 0 -5.348190 -1.899033 3.386230

H 0 -6.536636 -0.645706 3.022781

H 0 -1.781708 2.994690 -5.254776

H 0 -2.436478 3.713318 -3.792465

H 0 1.343979 -4.491867 2.176941

H 0 1.193314 -3.275131 0.089310

H 0 0.849747 0.440582 2.267059

H 0 0.998291 -0.764435 4.360336

H 0 0.541458 -5.370575 4.185242

H 0 1.473724 -5.238846 5.685272

H 0 2.313507 -5.217667 4.128021

H 0 2.010712 -1.972946 6.028488

H 0 1.305939 -3.428696 6.745651

H 0 0.245026 -2.194885 6.047089

C 0 3.332581 0.889511 -0.809097

C 0 3.825104 1.207108 -2.080704

C 0 3.792648 2.536072 -2.524124

C 0 3.271500 3.544083 -1.707260

C 0 2.775391 3.219467 -0.438370

C 0 2.802597 1.896835 0.011368

N 0 3.342834 -0.486588 -0.286423

C 0 3.533295 -1.557312 -1.311523

C 0 4.299942 -0.669281 0.860687

H 0 4.242172 0.445313 -2.727158

H 0 4.187363 2.778763 -3.504913

H 0 3.257645 4.572342 -2.051575

H 0 2.368774 3.994095 0.202698

H 0 2.413177 1.649887 0.992203

H 0 2.788742 -1.435867 -2.099625

H 0 3.379730 -2.518784 -0.819382

H 0 4.541275 -1.528015 -1.734543

H 0 5.331284 -0.582611 0.506668

H 0 4.129562 -1.656738 1.293777

H 0 4.107162 0.090744 1.616516

H 0 2.184204 -0.750488 0.200119

Int1_A-2_

Au 0 -0.355652 0.354477 0.485107

C 0 -0.191223 4.312546 -0.268997

C 0 1.964523 4.391800 1.041672

C 0 2.210922 3.328812 3.187500

C 0 2.769409 4.087692 2.148132

C 0 0.098648 3.225388 2.027435

C 0 0.325378 2.049179 4.279556

C 0 0.627640 3.975616 0.958023

C 0 0.881927 2.887222 3.150543

N 0 -2.966812 1.230576 1.776093

N 0 -1.297913 2.812637 2.005432

C 0 -2.275726 3.873337 2.443970

C 0 -3.848251 1.022781 0.599442

C 0 -4.146118 2.425003 0.010986

C 0 -3.677383 1.933594 2.872314

C 0 -3.745850 3.400467 2.437851

C 0 -1.666931 1.619751 1.493073

C 0 4.197708 4.576782 2.230255

C 0 -4.536392 3.472458 1.093979

C 0 3.307297 -2.674667 -0.912903

C 0 2.165268 -3.159805 -0.220963

C 0 1.087173 -2.317444 0.064529

C 0 1.052948 -0.946487 -0.308990

C 0 2.202637 -0.488831 -1.006592

C 0 3.291580 -1.310318 -1.306503

N 0 4.388824 -3.496964 -1.190491

C 0 4.406647 -4.878098 -0.704330

C 0 5.581146 -2.949448 -1.840622

H 0 -0.960724 5.068344 -0.063828

H 0 -0.698868 3.425507 -0.664764

H 0 0.452072 4.717422 -1.055634

H 0 2.385162 4.965515 0.220734

H 0 2.823425 3.070633 4.046402

H 0 1.057831 1.954285 5.085388

H 0 0.071426 1.038193 3.938110

H 0 -0.584595 2.486008 4.708800

H 0 -2.125977 4.737473 1.789185

H 0 -1.994095 4.175278 3.458282

H 0 -4.754807 0.509033 0.933228

H 0 -3.329681 0.391632 -0.127487

H 0 -3.243713 2.759903 -0.518021

H 0 -4.946838 2.363510 -0.736115

H 0 -3.118088 1.799637 3.802780

H 0 -4.661545 1.475113 2.991592

H 0 -4.284225 4.015152 3.169403

H 0 4.587341 4.844650 1.243408

H 0 4.857193 3.815674 2.660660

H 0 4.273666 5.468536 2.866409

H 0 -5.595551 3.331009 1.351379

H 0 -4.457535 4.479828 0.667068

H 0 2.133865 -4.185989 0.124695

H 0 0.258575 -2.735336 0.632797

H 0 2.264679 0.556122 -1.302643

H 0 4.143555 -0.882889 -1.821014

H 0 4.355316 -4.929474 0.392273

H 0 5.332672 -5.357529 -1.022903

H 0 3.569397 -5.460129 -1.112076

H 0 5.346313 -2.533951 -2.829498

H 0 6.311279 -3.747406 -1.978775

H 0 6.050705 -2.157257 -1.240707

C 0 -1.072693 -0.419174 -3.697723

C 0 -1.817703 -0.719040 -4.841690

C 0 -2.216476 0.324219 -5.683411

C 0 -1.868591 1.646683 -5.380478

C 0 -1.121353 1.928284 -4.231873

C 0 -0.716965 0.893219 -3.379852

N 0 -0.618973 -1.519562 -2.798538

C 0 -1.764557 -2.321900 -2.215149

C 0 0.404449 -2.423645 -3.466217

H 0 -2.086685 -1.740723 -5.087753

H 0 -2.793808 0.102280 -6.573746

H 0 -2.177414 2.451279 -6.038437

H 0 -0.848465 2.950700 -3.995651

H 0 -0.145386 1.105804 -2.483032

H 0 -0.124359 -1.096405 -1.961868

H 0 -2.448456 -1.633026 -1.720900

H 0 -1.347916 -3.017395 -1.486374

H 0 -2.274460 -2.861649 -3.012619

H 0 -0.071487 -2.951080 -4.293076

H 0 0.777390 -3.120911 -2.715775

H 0 1.222015 -1.802643 -3.828705

TS2_A-2_

Au 0 -0.999390 0.816650 -0.122910

C 0 -1.625397 4.320389 1.436768

C 0 -2.470932 3.297638 3.584930

C 0 -4.117554 1.541382 3.619080

C 0 -3.271347 2.412292 4.320572

C 0 -3.369720 2.451004 1.513474

C 0 -5.098648 0.579819 1.495758

C 0 -2.505890 3.341766 2.183105

C 0 -4.185455 1.542847 2.218933

N 0 -3.078125 1.668518 -2.065460

N 0 -3.479813 2.493011 0.064407

C 0 -4.429672 3.541794 -0.452927

C 0 -2.151000 2.237534 -3.079132

C 0 -2.199844 3.778015 -2.937195

C 0 -4.487808 2.021729 -2.358521

C 0 -4.634995 3.499878 -1.983673

C 0 -2.672409 1.752151 -0.734833

C 0 -3.244247 2.416000 5.832977

C 0 -3.649658 4.329742 -2.859665

C 0 1.188644 0.464935 0.170166

C 0 0.819489 -0.815918 0.733353

C 0 1.028717 -2.047287 -0.078796

C 0 1.299927 -3.299988 0.522308

C 0 1.021057 -1.999863 -1.498749

C 0 1.518768 -4.445557 -0.248367

C 0 1.229980 -3.155487 -2.268570

C 0 1.484222 -4.385361 -1.649887

C 0 0.960236 -0.917923 2.241013

H 0 -2.207504 5.126648 0.970886

H 0 -1.055771 3.820572 0.646027

H 0 -0.914749 4.792587 2.121323

H 0 -1.805023 3.975677 4.111526

H 0 -4.740875 0.843887 4.171234

H 0 -5.691360 0.002457 2.210144

H 0 -4.525100 -0.131622 0.891861

H 0 -5.797150 1.099060 0.827682

H 0 -4.052368 4.517166 -0.125656

H 0 -5.388641 3.375046 0.048721

H 0 -2.481293 1.902924 -4.068039

H 0 -1.145050 1.855957 -2.891296

H 0 -1.652435 4.042831 -2.022720

H 0 -1.672318 4.255676 -3.772217

H 0 -5.146484 1.376068 -1.770706

H 0 -4.673950 1.832123 -3.418457

H 0 -5.648743 3.867462 -2.186661

H 0 -4.022980 3.074524 6.240768

H 0 -2.283127 2.773666 6.216049

H 0 -3.421295 1.415009 6.240219

H 0 -4.073792 4.354156 -3.873917

H 0 -3.625946 5.369858 -2.511032

H 0 1.515076 1.238190 0.860794

H 0 1.653793 0.517059 -0.810043

H 0 1.298218 -3.388504 1.601578

H 0 0.738907 -1.075729 -1.993423

H 0 1.695800 -5.394562 0.248322

H 0 1.163666 -3.095551 -3.351196

H 0 1.628235 -5.281738 -2.243576

H 0 0.437622 -1.772003 2.669083

H 0 2.027573 -0.985367 2.506454

H 0 0.567245 -0.014648 2.715012

C 0 -3.648483 -3.330582 1.477554

C 0 -3.105255 -3.260361 0.169296

C 0 -2.081619 -2.355286 -0.133316

C 0 -1.560776 -1.477905 0.829880

C 0 -2.107758 -1.535721 2.120956

C 0 -3.126450 -2.440079 2.448837

N 0 -4.656754 -4.239517 1.795135

C 0 -5.175232 -5.145966 0.774139

C 0 -5.197083 -4.290314 3.150726

H 0 -3.476028 -3.912415 -0.612700

H 0 -1.691498 -2.348343 -1.148819

H 0 -1.761124 -0.860962 2.899384

H 0 -3.514740 -2.444626 3.460602

H 0 -5.622009 -4.600876 -0.069931

H 0 -5.948883 -5.777222 1.213821

H 0 -4.390289 -5.802795 0.372284

H 0 -4.423813 -4.538025 3.892120

H 0 -5.969589 -5.059052 3.201233

H 0 -5.652328 -3.333893 3.447006

C 0 4.828537 -0.593033 -2.181259

C 0 6.194244 -0.299408 -2.190979

C 0 6.673294 0.647461 -3.102005

C 0 5.793793 1.285446 -3.985764

C 0 4.429688 0.976822 -3.962143

C 0 3.937271 0.030228 -3.055756

N 0 4.295029 -1.606842 -1.217621

C 0 4.848083 -3.000458 -1.480331

C 0 4.493774 -1.193619 0.234222

H 0 6.883743 -0.787537 -1.510712

H 0 7.730820 0.884613 -3.119034

H 0 6.171478 2.019104 -4.688705

H 0 3.746063 1.468475 -4.644562

H 0 2.880249 -0.211563 -3.036652

H 0 3.264908 -1.667725 -1.359024

H 0 4.670685 -3.248169 -2.525940

H 0 4.321472 -3.701996 -0.834244

H 0 5.916550 -3.001877 -1.267334

H 0 5.560104 -1.090012 0.429794

H 0 4.058075 -1.966904 0.866700

H 0 3.973373 -0.248352 0.390671

Int2_A-2_

Au 0 -0.700012 -0.576385 0.263123

C 0 -0.363617 -4.221252 1.767319

C 0 1.458603 -3.406723 3.311218

C 0 3.317184 -2.091125 2.531052

C 0 2.654587 -2.728622 3.589386

C 0 1.625519 -2.822632 0.977127

C 0 3.546677 -1.378479 0.117645

C 0 0.929291 -3.476028 2.015274

C 0 2.822159 -2.119263 1.218872

N 0 0.040756 -2.156600 -2.269165

N 0 1.142075 -2.929703 -0.393295

C 0 1.620331 -4.170380 -1.109406

C 0 -1.322098 -2.548874 -2.704742

C 0 -1.496964 -4.052612 -2.374557

C 0 1.087234 -2.836563 -3.071686

C 0 1.123108 -4.281342 -2.565842

C 0 0.229782 -2.072510 -0.894608

C 0 3.226135 -2.708984 4.988998

C 0 -0.279739 -4.916580 -2.809860

C 0 -1.411911 0.969040 1.529541

C 0 -0.877502 2.435272 1.336258

C 0 -1.384476 2.995178 -0.014755

C 0 -2.436172 3.926956 -0.106110

C 0 -0.783051 2.577225 -1.221100

C 0 -2.874283 4.417526 -1.348038

C 0 -1.211472 3.065674 -2.459442

C 0 -2.265320 3.989685 -2.531158

C 0 -1.426041 3.275970 2.532745

C 0 3.492844 2.947311 1.733612

C 0 2.673859 3.926025 1.116898

C 0 1.304306 3.722534 0.970398

C 0 0.664352 2.546326 1.407608

C 0 1.478134 1.580765 2.020325

C 0 2.854584 1.767105 2.182510

N 0 4.861511 3.142838 1.891205

C 0 5.482315 4.386475 1.437302

C 0 5.670547 2.137054 2.571915

H 0 -0.215317 -5.120880 1.155838

H 0 -1.092682 -3.588272 1.249023

H 0 -0.804382 -4.544205 2.714615

H 0 0.925812 -3.898010 4.120361

H 0 4.243835 -1.559692 2.727234

H 0 4.536621 -1.060654 0.455276

H 0 2.996567 -0.476471 -0.176834

H 0 3.683090 -1.991638 -0.780396

H 0 1.303360 -5.026306 -0.504852

H 0 2.714859 -4.146927 -1.096619

H 0 -1.415695 -2.345627 -3.776321

H 0 -2.053726 -1.947250 -2.159607

H 0 -1.641602 -4.139633 -1.289520

H 0 -2.406296 -4.442932 -2.847717

H 0 2.039215 -2.318665 -2.923325

H 0 0.813600 -2.759766 -4.126617

H 0 1.857941 -4.883453 -3.113983

H 0 3.877777 -3.576019 5.161240

H 0 2.435333 -2.741531 5.745193

H 0 3.826065 -1.810608 5.164612

H 0 -0.351700 -5.107162 -3.890015

H 0 -0.337875 -5.896744 -2.321106

H 0 -1.132263 0.629730 2.538803

H 0 -2.510239 1.048050 1.573761

H 0 -2.901200 4.310913 0.794495

H 0 0.049744 1.884033 -1.179504

H 0 -3.672760 5.152893 -1.381531

H 0 -0.710327 2.741821 -3.366837

H 0 -2.586197 4.385100 -3.489639

H 0 -1.147949 4.332016 2.446213

H 0 -2.517715 3.205719 2.616974

H 0 -0.996124 2.893295 3.463089

H 0 3.103531 4.852661 0.758377

H 0 0.718109 4.505768 0.499298

H 0 1.047226 0.658905 2.394165

H 0 3.424973 0.988342 2.672287

H 0 5.339081 4.540909 0.359085

H 0 6.555191 4.342941 1.629761

H 0 5.078598 5.264297 1.961929

H 0 5.337997 1.971405 3.607100

H 0 6.708923 2.470000 2.601028

H 0 5.641953 1.169250 2.049271

C 0 -4.805649 -1.171219 -0.367737

C 0 -5.964233 -1.475464 -1.086517

C 0 -6.573303 -2.719574 -0.889526

C 0 -6.029678 -3.636795 0.018234

C 0 -4.872223 -3.311523 0.734146

C 0 -4.251434 -2.070663 0.546143

N 0 -4.156662 0.168472 -0.520844

C 0 -3.958206 0.597977 -1.964859

C 0 -4.915619 1.237152 0.255524

H 0 -6.398666 -0.770889 -1.786560

H 0 -7.472702 -2.966766 -1.441971

H 0 -6.509476 -4.597275 0.169113

H 0 -4.451813 -4.016541 1.442383

H 0 -3.350540 -1.812866 1.094635

H 0 -3.196091 0.098785 -0.110962

H 0 -3.456909 -0.207214 -2.498932

H 0 -3.339508 1.495026 -1.973175

H 0 -4.929200 0.804794 -2.412994

H 0 -5.918700 1.315200 -0.164841

H 0 -4.377136 2.179962 0.156204

H 0 -4.969528 0.932587 1.300034

TS1_B_

Au 0 1.501755 -1.086166 -0.582642

C 0 2.164505 1.131042 2.671143

C 0 1.488480 3.203140 1.399139

C 0 2.323196 3.700241 -0.803696

C 0 1.520416 4.058838 0.289627

C 0 3.050934 1.703781 0.339400

C 0 3.938095 2.173950 -2.006577

C 0 2.245697 2.022980 1.451691

C 0 3.096359 2.531769 -0.802002

N 0 4.438080 -1.628082 -0.283615

N 0 3.896866 0.520187 0.368835

C 0 5.194550 0.683350 1.123291

C 0 4.259064 -2.897400 0.473251

C 0 4.535172 -2.557098 1.959305

C 0 5.853821 -1.185776 -0.284058

C 0 6.082703 -0.580475 1.103928

C 0 3.466843 -0.667831 -0.103409

C 0 0.729095 5.347519 0.277237

C 0 5.814255 -1.692429 2.167389

C 0 -1.275665 -0.440872 -1.038727

C 0 -0.525528 -1.709076 -1.201355

C 0 -0.876068 -2.814194 -0.235626

C 0 -0.975098 -4.162384 -0.649063

C 0 -1.118103 -2.540054 1.133881

C 0 -1.303513 -5.178650 0.255493

C 0 -1.447250 -3.554352 2.034790

C 0 -1.544052 -4.883133 1.601425

C 0 -0.408371 -2.136078 -2.672363

H 0 3.060303 1.207108 3.301483

H 0 2.043274 0.078629 2.393753

H 0 1.312469 1.414780 3.295471

H 0 0.862427 3.458527 2.249420

H 0 2.353119 4.345016 -1.677414

H 0 3.895691 2.969345 -2.755280

H 0 3.589310 1.248474 -2.480560

H 0 4.992050 2.024292 -1.742630

H 0 4.935104 0.980438 2.143951

H 0 5.734634 1.516479 0.661377

H 0 4.959167 -3.635895 0.072876

H 0 3.239670 -3.265457 0.331543

H 0 3.657959 -2.018906 2.342850

H 0 4.622589 -3.476852 2.549591

H 0 6.001236 -0.458221 -1.087509

H 0 6.483597 -2.054993 -0.483337

H 0 7.118835 -0.245117 1.233261

H 0 -0.091537 5.321335 1.000870

H 0 0.305710 5.549667 -0.712524

H 0 1.366165 6.203415 0.536377

H 0 6.690735 -2.355057 2.144775

H 0 5.791138 -1.250870 3.171143

H 0 -0.935867 0.351135 -1.706543

H 0 -1.404419 -0.070892 -0.023712

H 0 -0.796738 -4.426376 -1.684265

H 0 -1.020538 -1.523849 1.502411

H 0 -1.372437 -6.203415 -0.095963

H 0 -1.619537 -3.309875 3.078659

H 0 -1.795776 -5.672958 2.301254

H 0 0.358749 -2.900757 -2.824524

H 0 -1.358032 -2.550262 -3.053802

H 0 -0.142990 -1.279922 -3.301498

C 0 -3.047729 -0.571365 -1.589798

C 0 -3.755646 -1.436234 -0.662598

C 0 -4.642578 -0.946533 0.264587

C 0 -4.980591 0.446060 0.299164

C 0 -4.393845 1.303131 -0.695343

C 0 -3.515945 0.803040 -1.620500

N 0 -5.852173 0.937347 1.221558

C 0 -6.242096 2.359360 1.211151

C 0 -6.469727 0.048248 2.222885

H 0 -2.834229 -1.012558 -2.565002

H 0 -3.558228 -2.501129 -0.692307

H 0 -5.113678 -1.633560 0.954086

H 0 -4.670029 2.347961 -0.737198

H 0 -3.110580 1.468155 -2.377792

H 0 -6.917572 2.547577 2.044098

H 0 -5.367600 3.008682 1.326614

H 0 -6.760162 2.624207 0.282532

H 0 -7.118835 -0.696838 1.748703

H 0 -5.705460 -0.471375 2.810028

H 0 -7.074066 0.646133 2.903244

TS2_B_

Au 0 1.709229 2.702057 -0.362198

C 0 1.534424 2.255875 -4.174698

C 0 2.203659 -0.171112 -3.999130

C 0 4.075317 -0.905411 -2.674622

C 0 3.005310 -1.219100 -3.525070

C 0 3.548828 1.445618 -2.821304

C 0 5.518417 0.714035 -1.377090

C 0 2.453781 1.168427 -3.665359

C 0 4.366852 0.416672 -2.311554

N 0 3.943069 4.628906 -1.054337

N 0 3.885056 2.821655 -2.488907

C 0 4.713684 3.536682 -3.527252

C 0 3.062454 5.827316 -1.051376

C 0 2.761551 6.149200 -2.536972

C 0 5.278610 4.933334 -1.622100

C 0 5.068481 4.988449 -3.138351

C 0 3.340790 3.439911 -1.417633

C 0 2.740036 -2.648773 -3.941833

C 0 4.028488 6.113739 -3.442413

C 0 0.454330 0.650635 1.403305

C 0 -0.012619 1.977814 0.752000

C 0 -1.079391 1.828903 -0.312378

C 0 -2.093277 2.800705 -0.496307

C 0 -1.073059 0.746490 -1.231308

C 0 -3.036621 2.699188 -1.526184

C 0 -2.019104 0.641266 -2.255508

C 0 -3.015533 1.615845 -2.410965

C 0 -0.347153 3.059937 1.801071

C 0 -0.369705 -0.159058 2.507050

C 0 0.092361 -1.532669 2.628265

C 0 0.169647 -2.218262 3.817078

C 0 -0.151016 -1.572052 5.053894

C 0 -0.545334 -0.199402 4.993881

C 0 -0.621185 0.468277 3.792465

N 0 -0.071579 -2.237442 6.240982

C 0 -0.425583 -1.564804 7.502365

C 0 0.357574 -3.645096 6.286438

H 0 2.060547 3.000137 -4.784744

H 0 1.056473 2.787933 -3.343903

H 0 0.742233 1.827667 -4.794540

H 0 1.361023 -0.396744 -4.646683

H 0 4.700653 -1.704620 -2.286774

H 0 6.043076 -0.206619 -1.107498

H 0 5.170700 1.187698 -0.451172

H 0 6.253311 1.391235 -1.830048

H 0 4.155045 3.493530 -4.467575

H 0 5.635529 2.961060 -3.664047

H 0 3.589340 6.640472 -0.543610

H 0 2.146591 5.600388 -0.499786

H 0 2.032166 5.409424 -2.892593

H 0 2.283615 7.132156 -2.625427

H 0 5.980011 4.147293 -1.327194

H 0 5.622100 5.880646 -1.201157

H 0 5.994446 5.245331 -3.667343

H 0 1.680298 -2.812927 -4.163116

H 0 3.039795 -3.357056 -3.162308

H 0 3.304321 -2.906265 -4.848100

H 0 4.566116 7.065765 -3.328278

H 0 3.719223 6.061523 -4.493652

H 0 1.412735 0.849411 1.898636

H 0 0.681076 -0.076447 0.617050

H 0 -2.142532 3.658157 0.163574

H 0 -0.283936 0.003403 -1.178238

H 0 -3.791687 3.472565 -1.634415

H 0 -1.968460 -0.196503 -2.946000

H 0 -3.745621 1.538100 -3.209702

H 0 -0.376953 4.062027 1.361374

H 0 -1.328476 2.900894 2.283035

H 0 0.419678 3.082474 2.583206

H 0 0.403305 -2.039764 1.717636

H 0 0.504349 -3.247238 3.814941

H 0 -0.777985 0.334564 5.905289

H 0 -0.892548 1.516205 3.807480

H 0 -0.318146 -2.271606 8.323959

H 0 0.231873 -0.710297 7.698288

H 0 -1.464493 -1.215347 7.482025

H 0 -0.322495 -4.286362 5.713654

H 0 1.372665 -3.765167 5.891464

H 0 0.354370 -3.984009 7.321411

H 0 -1.661011 -0.378906 2.075958

C 0 -4.555206 -2.060867 5.581985

C 0 -4.492905 -0.705063 5.241150

C 0 -3.993300 -0.308533 3.998322

C 0 -3.546036 -1.269440 3.074158

C 0 -3.615311 -2.630310 3.414108

C 0 -4.116348 -3.017838 4.661774

N 0 -2.997696 -0.825272 1.790314

C 0 -2.769913 -1.909851 0.790558

C 0 -3.773300 0.309036 1.180893

H 0 -4.956512 -2.367004 6.541946

H 0 -4.842514 0.049179 5.938293

H 0 -3.957169 0.746338 3.759338

H 0 -3.297089 -3.396744 2.720688

H 0 -4.175858 -4.073822 4.903763

H 0 -2.375992 -1.449402 -0.115311

H 0 -2.036057 -2.619034 1.176758

H 0 -3.702133 -2.436661 0.560013

H 0 -4.842865 0.075562 1.189774

H 0 -3.432907 0.459778 0.160233

H 0 -3.594055 1.229858 1.733826

Int2_B_

Au 0 1.672852 -3.114700 0.214478

C 0 5.445953 -2.350052 1.227417

C 0 5.866409 -1.431229 -1.083817

C 0 5.171844 -2.582062 -3.080353

C 0 5.792175 -1.475449 -2.482986

C 0 4.757782 -3.566345 -0.915985

C 0 3.964462 -4.800491 -3.001465

C 0 5.360367 -2.462311 -0.278763

C 0 4.648605 -3.636765 -2.319839

N 0 2.566300 -5.949402 0.796112

N 0 4.263275 -4.683777 -0.126251

C 0 5.321045 -5.661545 0.319305

C 0 2.082794 -5.944809 2.202316

C 0 3.335190 -5.735229 3.090714

C 0 3.402832 -7.139191 0.508911

C 0 4.761963 -6.839264 1.147598

C 0 2.981781 -4.727982 0.300781

C 0 6.384109 -0.368942 -3.327057

C 0 4.540604 -6.632553 2.679703

C 0 1.080048 -0.156372 0.384247

C 0 0.276443 -1.469589 0.076797

C 0 -0.803375 -1.775574 1.093384

C 0 -2.053986 -2.332230 0.723251

C 0 -0.591888 -1.590317 2.486496

C 0 -3.007156 -2.715286 1.677521

C 0 -1.545502 -1.961761 3.437531

C 0 -2.765579 -2.533188 3.042892

C 0 -0.245743 -1.439407 -1.376465

C 0 0.334167 1.173508 0.326096

C 0 -0.100754 1.830963 1.488586

C 0 -0.688431 3.107330 1.455429

C 0 -0.850555 3.801910 0.227844

C 0 -0.448349 3.125595 -0.951523

C 0 0.132080 1.857635 -0.890167

N 0 -1.383591 5.102020 0.190826

C 0 -1.368027 5.837906 -1.082000

C 0 -1.369110 5.902313 1.425980

H 0 6.188354 -3.036163 1.656174

H 0 4.481583 -2.567917 1.698578

H 0 5.741409 -1.337800 1.517532

H 0 6.326355 -0.572617 -0.602600

H 0 5.091141 -2.627792 -4.162704

H 0 4.044907 -4.711243 -4.088196

H 0 2.898849 -4.841721 -2.745255

H 0 4.403534 -5.763611 -2.713943

H 0 6.069717 -5.092194 0.878815

H 0 5.807465 -6.051102 -0.581680

H 0 1.583420 -6.898361 2.397919

H 0 1.361649 -5.134109 2.333023

H 0 3.619507 -4.677383 3.011246

H 0 3.092056 -5.919510 4.144165

H 0 3.472504 -7.274658 -0.574524

H 0 2.910751 -8.015350 0.936584

H 0 5.461258 -7.675842 1.026855

H 0 6.381058 0.587448 -2.794235

H 0 5.831039 -0.238892 -4.263000

H 0 7.426468 -0.589981 -3.593491

H 0 4.389954 -7.635117 3.104767

H 0 5.455902 -6.239883 3.139648

H 0 1.904251 -0.112747 -0.338165

H 0 1.556396 -0.237152 1.367035

H 0 -2.269104 -2.516235 -0.322800

H 0 0.358810 -1.196777 2.830612

H 0 -3.935791 -3.173584 1.346939

H 0 -1.328247 -1.823914 4.493317

H 0 -3.494690 -2.846466 3.783386

H 0 -0.638992 -2.410126 -1.695175

H 0 -1.046234 -0.693787 -1.531830

H 0 0.570633 -1.189392 -2.063614

H 0 0.058624 1.361374 2.454590

H 0 -0.922180 3.593597 2.395737

H 0 -0.532364 3.610077 -1.915924

H 0 0.474106 1.402695 -1.814453

H 0 -1.859650 6.801453 -0.939285

H 0 -0.348694 6.019333 -1.454498

H 0 -1.922668 5.292023 -1.851746

H 0 -2.033157 5.473800 2.185715

H 0 -0.361374 5.985214 1.857758

H 0 -1.732132 6.906219 1.201584

C 0 -6.945633 4.441193 1.050568

C 0 -7.152817 3.089569 1.352493

C 0 -6.076004 2.197006 1.365158

C 0 -4.797287 2.679993 1.076294

C 0 -4.569397 4.021545 0.764709

C 0 -5.658356 4.902786 0.756424

N 0 -3.635193 1.738052 1.057693

C 0 -3.541412 0.859375 2.296997

C 0 -3.613251 0.898056 -0.213516

H 0 -7.784058 5.128540 1.043076

H 0 -8.149338 2.727249 1.578003

H 0 -6.246521 1.152206 1.598862

H 0 -3.570755 4.381760 0.521179

H 0 -5.496002 5.947998 0.518372

H 0 -2.762436 2.304382 1.053253

H 0 -2.581223 0.344696 2.278931

H 0 -3.625519 1.498322 3.175766

H 0 -4.342178 0.123383 2.280304

H 0 -4.543060 0.332062 -0.266479

H 0 -2.760513 0.222229 -0.164825

H 0 -3.528091 1.572327 -1.064835

TS3_B_

Au 0 1.318008 -0.319122 -1.764374

C 0 3.077988 -3.880096 -1.741089

C 0 3.579987 -3.536560 0.706558

C 0 4.556946 -1.507828 1.560715

C 0 4.060425 -2.797958 1.798172

C 0 4.106415 -1.729187 -0.798233

C 0 5.135727 0.443283 0.061584

C 0 3.597870 -3.030853 -0.601318

C 0 4.592560 -0.952896 0.273468

N 0 3.431747 0.236191 -3.824249

N 0 4.175262 -1.173309 -2.143814

C 0 5.367722 -1.617126 -2.957184

C 0 2.605896 -0.140182 -5.008728

C 0 3.071365 -1.551407 -5.444672

C 0 4.859863 0.420868 -4.190521

C 0 5.420731 -0.993286 -4.368988

C 0 3.163925 -0.452484 -2.658279

C 0 4.063507 -3.389648 3.189133

C 0 4.618698 -1.694200 -5.509857

H 0 3.893814 -4.330185 -2.322403

H 0 2.456894 -3.302521 -2.433060

H 0 2.469742 -4.703278 -1.356979

H 0 3.182266 -4.533371 0.873367

H 0 4.930450 -0.919876 2.393921

H 0 5.489319 0.865005 1.005966

H 0 4.372787 1.120361 -0.341583

H 0 5.979965 0.455200 -0.639130

H 0 5.331055 -2.709900 -2.998154

H 0 6.263000 -1.335052 -2.394211

H 0 2.764709 0.612518 -5.785858

H 0 1.548950 -0.139496 -4.728012

H 0 2.662933 -2.274521 -4.725937

H 0 2.643295 -1.804153 -6.421661

H 0 5.363876 0.972351 -3.391388

H 0 4.902435 1.017319 -5.104004

H 0 6.474548 -0.972000 -4.670975

H 0 4.107742 -2.611450 3.956909

H 0 4.933075 -4.043839 3.335388

H 0 3.169144 -3.995117 3.369125

H 0 4.972733 -1.254013 -6.452408

H 0 4.881226 -2.757996 -5.551849

C 0 -0.612289 -2.156387 -0.986984

C 0 -0.819031 -0.568268 -0.785553

C 0 -0.961578 -0.308609 0.714493

C 0 -2.219467 -0.147202 1.321564

C 0 0.175049 -0.316635 1.547409

C 0 -2.336685 0.006470 2.708435

C 0 0.062439 -0.159393 2.932846

C 0 -1.198746 0.004974 3.521103

C 0 -2.029343 -0.143997 -1.643509

C 0 -1.783035 -3.018677 -0.542358

C 0 -1.906769 -3.468170 0.784546

C 0 -2.762375 -3.447266 -1.455948

C 0 -2.959595 -4.285828 1.189209

C 0 -3.825775 -4.262833 -1.072342

C 0 -3.957047 -4.701614 0.269928

N 0 -5.012589 -5.511673 0.665726

C 0 -5.115692 -5.958420 2.054321

C 0 -6.020325 -5.932541 -0.306870

H 0 -0.432083 -2.351730 -2.052597

H 0 0.283371 -2.467834 -0.438034

H 0 -3.117767 -0.144318 0.716827

H 0 1.158691 -0.455917 1.104477

H 0 -3.320663 0.124863 3.150772

H 0 0.954865 -0.170761 3.551224

H 0 -1.290680 0.123962 4.595749

H 0 -2.324249 0.893600 -1.475021

H 0 -1.798203 -0.271927 -2.707291

H 0 -2.898315 -0.773514 -1.424545

H 0 -1.163757 -3.174561 1.520126

H 0 -2.688416 -3.147491 -2.498672

H 0 -3.003204 -4.605194 2.222458

H 0 -4.547500 -4.566100 -1.819763

H 0 -6.011719 -6.569611 2.168518

H 0 -4.250488 -6.566284 2.354385

H 0 -5.193298 -5.111221 2.749710

H 0 -5.580643 -6.524338 -1.122025

H 0 -6.765594 -6.551620 0.193970

H 0 -6.539413 -5.072861 -0.753067

H 0 -0.114197 0.843857 -1.020798

C 0 -3.557388 4.259674 0.754593

C 0 -3.415253 4.207092 -0.633667

C 0 -2.295227 3.599136 -1.213013

C 0 -1.301392 3.021439 -0.402985

C 0 -1.452393 3.069031 0.993454

C 0 -2.569748 3.685349 1.561584

N 0 -0.109253 2.385956 -0.994232

C 0 1.141922 2.716812 -0.229691

C 0 0.085556 2.726364 -2.439178

H 0 -4.421814 4.738617 1.200745

H 0 -4.169647 4.646606 -1.277512

H 0 -2.216675 3.592224 -2.291397

H 0 -0.726196 2.607437 1.648544

H 0 -2.666306 3.707855 2.641632

H 0 1.271667 3.802414 -0.161316

H 0 1.094437 2.286026 0.767838

H 0 1.990600 2.275482 -0.754440

H 0 1.025528 2.285797 -2.773514

H 0 -0.723679 2.303940 -3.035019

H 0 0.121567 3.812073 -2.582779

Pro_B_

Au 0 -2.214035 1.864487 -0.127411

C 0 -4.735474 3.591904 -2.643295

C 0 -2.760895 4.577927 -3.866013

C 0 -0.938919 5.667068 -2.727539

C 0 -1.532730 5.251205 -3.929260

C 0 -2.784302 4.767059 -1.465729

C 0 -0.864243 5.892731 -0.211243

C 0 -3.412033 4.326416 -2.649063

C 0 -1.543457 5.438293 -1.484589

N 0 -3.633774 3.511353 1.873200

N 0 -3.439621 4.561096 -0.180008

C 0 -4.506958 5.573746 0.175064

C 0 -4.632706 2.485367 2.291138

C 0 -5.968109 2.886246 1.617294

C 0 -3.939789 4.851669 2.436890

C 0 -5.101334 5.382095 1.588898

C 0 -3.208755 3.477798 0.574600

C 0 -0.873428 5.541779 -5.258011

C 0 -6.302185 4.399277 1.751465

H 0 -5.580307 4.264038 -2.442490

H 0 -4.760803 2.793167 -1.893936

H 0 -4.919556 3.136444 -3.620117

H 0 -3.228058 4.238235 -4.785583

H 0 0.016647 6.182281 -2.756363

H 0 0.053833 6.439224 -0.441284

H 0 -0.593704 5.045197 0.430527

H 0 -1.502594 6.559509 0.381927

H 0 -5.274750 5.506241 -0.601105

H 0 -4.038574 6.559525 0.098495

H 0 -4.687164 2.495026 3.382751

H 0 -4.307190 1.495163 1.961807

H 0 -5.898270 2.609314 0.557785

H 0 -6.788345 2.292938 2.036118

H 0 -3.046204 5.478531 2.370514

H 0 -4.199844 4.724716 3.489182

H 0 -5.429855 6.369461 1.934525

H 0 -1.205276 4.843185 -6.032059

H 0 0.217239 5.478683 -5.186951

H 0 -1.117935 6.554703 -5.604309

H 0 -6.716965 4.582626 2.752487

H 0 -7.095642 4.657730 1.040131

C 0 -0.263794 -1.479950 -1.472504

C 0 -0.106277 -0.002289 -0.957611

C 0 0.520752 0.074005 0.432953

C 0 1.855423 0.465790 0.624207

C 0 -0.235367 -0.302185 1.561035

C 0 2.416214 0.487717 1.908020

C 0 0.322510 -0.278824 2.842697

C 0 1.654068 0.117691 3.020203

C 0 0.539000 0.910645 -2.011871

C 0 1.046494 -2.177460 -1.768311

C 0 1.769028 -2.842651 -0.761063

C 0 2.970000 -3.494858 -1.027847

C 0 3.515564 -3.513153 -2.338135

C 0 2.783478 -2.849548 -3.356552

C 0 1.583282 -2.203537 -3.067780

N 0 4.710541 -4.160690 -2.613708

C 0 5.235382 -4.186951 -3.979065

C 0 5.433945 -4.854492 -1.548035

H 0 -0.880783 -1.449753 -2.380295

H 0 -0.825882 -2.041809 -0.717041

H 0 -1.205536 0.322647 -0.853714

H 0 2.467148 0.747543 -0.224518

H 0 -1.268448 -0.620682 1.433731

H 0 3.450226 0.791687 2.034592

H 0 -0.278137 -0.569016 3.698914

H 0 2.090881 0.136368 4.013153

H 0 0.634354 1.940323 -1.652695

H 0 1.534485 0.541260 -2.274460

H 0 -0.060898 0.918427 -2.927994

H 0 1.381927 -2.858322 0.253708

H 0 3.479721 -3.999451 -0.217239

H 0 3.144012 -2.848755 -4.376953

H 0 1.043381 -1.722412 -3.879745

H 0 6.178574 -4.734283 -3.989380

H 0 4.545425 -4.686737 -4.673294

H 0 5.427887 -3.174484 -4.359695

H 0 5.716446 -4.169144 -0.737350

H 0 4.839645 -5.670258 -1.113235

H 0 6.348404 -5.285141 -1.957458

C 0 -5.159500 -0.466171 -1.092072

C 0 -4.117157 -1.266830 -0.603897

C 0 -4.192368 -1.866959 0.655655

C 0 -5.336578 -1.688232 1.478195

C 0 -6.388794 -0.878723 0.971222

C 0 -6.293915 -0.286926 -0.290039

N 0 -5.430389 -2.293732 2.723755

C 0 -6.653076 -2.159607 3.516373

C 0 -4.360992 -3.174438 3.194641

H 0 -5.105545 -0.031448 -2.084274

H 0 -3.249283 -1.462387 -1.226730

H 0 -3.381134 -2.504944 0.982864

H 0 -7.294052 -0.739777 1.548477

H 0 -7.130432 0.300705 -0.657333

H 0 -6.534851 -2.706390 4.452286

H 0 -6.861862 -1.109695 3.764130

H 0 -7.529968 -2.566300 2.993393

H 0 -4.220673 -4.042603 2.535339

H 0 -4.612564 -3.543518 4.189438

H 0 -3.402054 -2.642731 3.264984

Rea_C_

Au 0 0.396851 -1.632797 0.158859

C 0 1.010712 -4.156540 -2.893326

C 0 -1.050674 -3.185822 -3.971664

C 0 -3.120529 -2.877060 -2.780441

C 0 -2.389450 -2.770096 -3.973465

C 0 -1.205292 -3.808029 -1.648621

C 0 -3.376892 -3.487335 -0.341873

C 0 -0.435303 -3.713898 -2.826721

C 0 -2.553589 -3.393066 -1.607834

N 0 0.202484 -4.186768 1.712234

N 0 -0.620300 -4.376328 -0.441208

C 0 -0.543732 -5.884277 -0.402618

C 0 1.622100 -4.194916 2.164352

C 0 2.361542 -5.199814 1.246353

C 0 -0.471664 -5.479553 1.996185

C 0 0.040222 -6.438660 0.915649

C 0 -0.054153 -3.599869 0.498032

C 0 -3.036423 -2.241837 -5.233307

C 0 1.592743 -6.540726 1.055161

C 0 0.929611 0.461792 -0.561462

C 0 0.787460 0.878235 0.759277

C 0 1.943634 0.941574 1.690323

C 0 1.764130 0.792740 3.082275

C 0 3.246567 1.186295 1.203247

C 0 2.852936 0.865143 3.953796

C 0 4.331131 1.269531 2.077591

C 0 4.139175 1.103943 3.455231

C 0 -0.546188 1.396423 1.241165

H 0 1.098785 -5.236572 -3.073044

H 0 1.556625 -3.927261 -1.972839

H 0 1.524872 -3.656540 -3.718872

H 0 -0.466339 -3.098923 -4.883118

H 0 -4.157074 -2.553802 -2.761063

H 0 -4.403793 -3.162704 -0.527939

H 0 -2.968872 -2.857834 0.458160

H 0 -3.422012 -4.513504 0.043839

H 0 0.046677 -6.192200 -1.270600

H 0 -1.562408 -6.259979 -0.541580

H 0 1.641052 -4.491455 3.216415

H 0 2.040863 -3.188660 2.076553

H 0 2.508423 -4.713928 0.272171

H 0 3.360474 -5.411545 1.644791

H 0 -1.554688 -5.334457 1.950012

H 0 -0.203979 -5.788254 3.008224

H 0 -0.369080 -7.447220 1.046890

H 0 -2.301270 -1.778183 -5.898285

H 0 -3.808090 -1.499222 -5.007339

H 0 -3.519516 -3.051910 -5.795670

H 0 1.780014 -7.177917 1.930481

H 0 2.010834 -7.075363 0.194031

H 0 0.136307 0.671814 -1.273788

H 0 1.914139 0.285645 -0.986800

H 0 0.776886 0.603867 3.487717

H 0 3.405441 1.352859 0.143845

H 0 2.697311 0.741333 5.020126

H 0 5.321915 1.475937 1.687485

H 0 4.982620 1.171005 4.134033

H 0 -0.859802 0.934998 2.182419

H 0 -0.458740 2.476242 1.420868

H 0 -1.329269 1.233841 0.497971

C 0 1.307541 4.639023 0.087097

C 0 0.523834 4.498900 -1.065430

C 0 -0.845947 4.768295 -1.045883

C 0 -1.484650 5.199707 0.146240

C 0 -0.681717 5.336334 1.309677

C 0 0.687485 5.061813 1.269867

N 0 -2.845612 5.481750 0.173264

C 0 -3.465805 5.991180 1.396423

C 0 -3.635971 5.400009 -1.054291

H 0 2.374847 4.448120 0.060211

H 0 0.984497 4.192520 -2.000549

H 0 -1.413803 4.665176 -1.961716

H 0 -1.120880 5.679321 2.237793

H 0 1.275894 5.194077 2.173157

H 0 -4.530685 6.147293 1.219589

H 0 -3.363586 5.280487 2.227814

H 0 -3.029007 6.948975 1.713135

H 0 -3.273819 6.093765 -1.826538

H 0 -3.622437 4.385895 -1.477127

H 0 -4.672470 5.655090 -0.830566

TS1_C_

Au 0 1.034805 -1.204544 -0.988556

C 0 2.647476 -0.372894 2.685455

C 0 2.328598 2.071442 2.153900

C 0 2.970520 3.032364 0.042786

C 0 2.422073 3.196609 1.323563

C 0 3.334656 0.682510 0.457260

C 0 4.000305 1.654724 -1.806686

C 0 2.780000 0.806610 1.747055

C 0 3.432785 1.790085 -0.411270

N 0 3.769333 -2.472931 -1.341721

N 0 3.851608 -0.599442 0.003800

C 0 5.212463 -0.965393 0.540527

C 0 3.345428 -3.848770 -0.978592

C 0 3.829147 -4.084015 0.474930

C 0 5.244766 -2.364319 -1.444580

C 0 5.744629 -2.311630 0.002060

C 0 3.084778 -1.444839 -0.720703

C 0 1.965622 4.556946 1.800873

C 0 5.298904 -3.627197 0.716522

C 0 -0.999451 -0.884232 -1.475037

C 0 -2.063232 -0.588500 -0.407990

C 0 -3.335251 0.034988 -0.917572

C 0 -3.708908 -0.113358 -2.275208

C 0 -4.171692 0.822037 -0.094193

C 0 -4.852264 0.500778 -2.784836

C 0 -5.317245 1.441681 -0.604706

C 0 -5.665344 1.282028 -1.950531

C 0 -1.501556 -0.002441 0.873840

H 0 3.583221 -0.579529 3.222244

H 0 2.361755 -1.283905 2.150574

H 0 1.885880 -0.172394 3.444977

H 0 1.894638 2.176727 3.144531

H 0 3.041367 3.889908 -0.620132

H 0 4.055923 2.631332 -2.294662

H 0 3.377625 1.002838 -2.431259

H 0 5.011246 1.229034 -1.801392

H 0 5.137009 -0.968109 1.632233

H 0 5.901581 -0.162506 0.257706

H 0 3.795212 -4.548767 -1.688522

H 0 2.257370 -3.920593 -1.054092

H 0 3.156693 -3.523911 1.139297

H 0 3.732849 -5.142792 0.744431

H 0 5.497971 -1.460693 -2.006592

H 0 5.615417 -3.230774 -1.996262

H 0 6.839325 -2.266525 0.052246

H 0 1.219528 4.473938 2.597488

H 0 1.528992 5.142807 0.985260

H 0 2.807587 5.137009 2.201569

H 0 5.972504 -4.416092 0.353409

H 0 5.478088 -3.545319 1.795624

H 0 -1.321442 -1.696976 -2.140656

H 0 -0.981827 0.014572 -2.114746

H 0 -3.087280 -0.699387 -2.940552

H 0 -3.923157 0.978638 0.947678

H 0 -5.106140 0.380127 -3.832672

H 0 -5.929535 2.057816 0.045670

H 0 -6.549820 1.768234 -2.348450

H 0 -0.718613 -0.645096 1.276794

H 0 -1.020096 0.950226 0.614090

H 0 -2.242172 0.186844 1.651917

C 0 -1.123642 -3.338226 1.769714

C 0 -2.032318 -2.950027 2.793000

C 0 -3.289368 -2.456512 2.503662

C 0 -3.723618 -2.329559 1.147781

C 0 -2.715897 -2.487747 0.101074

C 0 -1.480026 -3.148453 0.458694

N 0 -5.024933 -2.051605 0.869736

C 0 -5.676193 -2.316452 -0.427994

C 0 -5.970657 -1.668610 1.937225

H 0 -0.173737 -3.788818 2.030700

H 0 -1.752106 -3.088669 3.832672

H 0 -3.975388 -2.264175 3.316879

H 0 -3.067337 -2.674011 -0.905900

H 0 -0.811127 -3.434784 -0.341797

H 0 -5.003738 -2.828903 -1.111374

H 0 -6.545303 -2.961212 -0.256042

H 0 -6.009827 -1.384125 -0.889603

H 0 -6.305527 -2.542374 2.509964

H 0 -5.518463 -0.944427 2.616806

H 0 -6.839340 -1.199800 1.473450

Int1_C_

Au 0 0.993927 -2.461945 -1.021744

C 0 2.318344 -3.460114 2.695328

C 0 4.448120 -2.191269 2.223557

C 0 5.665680 -2.432602 0.161240

C 0 5.544724 -1.837158 1.425262

C 0 3.657013 -3.717957 0.532043

C 0 4.893677 -3.978104 -1.683182

C 0 3.493668 -3.128830 1.802032

C 0 4.738159 -3.373871 -0.305710

N 0 1.002853 -5.484924 -1.267822

N 0 2.723312 -4.736191 0.076218

C 0 2.973465 -6.115372 0.632339

C 0 -0.423584 -5.680145 -0.903458

C 0 -0.435028 -6.191940 0.559235

C 0 1.727951 -6.776047 -1.343735

C 0 1.983902 -7.180054 0.110870

C 0 1.639343 -4.413879 -0.664963

C 0 6.582520 -0.855133 1.920837

C 0 0.602112 -7.323792 0.824066

C 0 0.423080 -0.517761 -1.633698

C 0 0.398315 0.684357 -0.633835

C 0 0.534622 2.018234 -1.393646

C 0 -0.121948 2.192047 -2.631744

C 0 1.331863 3.081558 -0.926743

C 0 0.007965 3.373154 -3.365143

C 0 1.463196 4.269516 -1.659119

C 0 0.799530 4.423492 -2.880005

C 0 1.485748 0.517685 0.442078

H 0 2.417755 -4.446671 3.166962

H 0 1.378098 -3.454361 2.133881

H 0 2.236877 -2.727768 3.503937

H 0 4.329941 -1.728455 3.199539

H 0 6.500778 -2.160477 -0.477829

H 0 5.827881 -3.648193 -2.145264

H 0 4.069641 -3.682816 -2.344100

H 0 4.907379 -5.074432 -1.653030

H 0 2.941818 -6.032883 1.723297

H 0 3.993300 -6.400558 0.352127

H 0 -0.864014 -6.397583 -1.602097

H 0 -0.951843 -4.728043 -0.997009

H 0 -0.216600 -5.333374 1.208893

H 0 -1.435272 -6.550064 0.832031

H 0 2.655426 -6.632263 -1.905579

H 0 1.102859 -7.489426 -1.885086

H 0 2.491043 -8.150162 0.180481

H 0 6.165985 -0.174683 2.670441

H 0 6.987700 -0.252197 1.101547

H 0 7.428207 -1.376968 2.388306

H 0 0.175323 -8.274979 0.475815

H 0 0.749207 -7.432907 1.905685

H 0 -0.542679 -0.602173 -2.151093

H 0 1.153961 -0.252487 -2.411530

H 0 -0.735245 1.392181 -3.029541

H 0 1.880600 2.988052 0.001923

H 0 -0.497467 3.471313 -4.320969

H 0 2.096878 5.065277 -1.280151

H 0 0.908783 5.338440 -3.452927

H 0 1.335114 -0.410980 0.993973

H 0 2.459396 0.441208 -0.052811

H 0 1.525986 1.343506 1.158630

C 0 -1.315277 -1.040451 1.912506

C 0 -1.038498 -0.019043 2.875702

C 0 -0.970123 1.313370 2.537674

C 0 -1.206070 1.726227 1.186615

C 0 -1.148727 0.678421 0.111252

C 0 -1.404434 -0.704742 0.596939

N 0 -1.434814 3.022522 0.914246

C 0 -1.217834 4.073257 1.936218

C 0 -2.081741 3.534225 -0.314789

H 0 -1.459427 -2.062378 2.241592

H 0 -0.932571 -0.296234 3.920380

H 0 -0.853210 2.046860 3.323135

H 0 -1.810471 0.897568 -0.727982

H 0 -1.627106 -1.447266 -0.155441

H 0 -1.167038 5.035889 1.427704

H 0 -2.040115 4.096695 2.660278

H 0 -0.274475 3.914810 2.460358

H 0 -1.383347 4.151611 -0.883163

H 0 -2.442017 2.727524 -0.944580

H 0 -2.939758 4.143143 -0.011368

C 0 -4.198074 -1.048600 -1.923309

C 0 -4.984146 -0.328339 -1.015121

C 0 -5.244354 1.030670 -1.204620

C 0 -4.717865 1.721848 -2.326645

C 0 -3.918793 0.983368 -3.238419

C 0 -3.671356 -0.377090 -3.033905

N 0 -4.975937 3.079651 -2.526260

C 0 -4.547226 3.723816 -3.770828

C 0 -5.979080 3.755020 -1.699051

H 0 -4.025223 -2.110687 -1.786682

H 0 -5.418915 -0.830582 -0.155991

H 0 -5.877609 1.545517 -0.493500

H 0 -3.520400 1.459702 -4.124954

H 0 -3.078629 -0.919067 -3.764800

H 0 -4.820648 4.779404 -3.738571

H 0 -3.458542 3.664536 -3.891251

H 0 -5.014191 3.274277 -4.659760

H 0 -6.974167 3.294632 -1.787400

H 0 -5.696426 3.744553 -0.638641

H 0 -6.056412 4.797180 -2.011856

TS2_C_

Au 0 0.193390 -1.591888 -0.444412

C 0 -1.676208 -4.642349 1.076813

C 0 -0.891479 -3.978119 3.380920

C 0 1.407547 -3.455704 3.866425

C 0 0.084244 -3.591446 4.310593

C 0 0.763107 -4.100464 1.632111

C 0 3.207672 -3.537933 2.095245

C 0 -0.579666 -4.242310 2.039124

C 0 1.770172 -3.706039 2.536026

N 0 1.746185 -3.798965 -1.889343

N 0 1.146027 -4.404129 0.259842

C 0 1.378479 -5.869049 -0.015289

C 0 0.872162 -3.799271 -3.094208

C 0 -0.082123 -5.011368 -2.955307

C 0 2.653946 -4.973328 -1.879471

C 0 1.781815 -6.165787 -1.475708

C 0 1.117249 -3.456177 -0.700317

C 0 -0.276505 -3.351074 5.759232

C 0 0.647003 -6.320526 -2.535522

C 0 -0.806580 0.235687 -0.061584

C 0 -1.298019 1.154587 -1.209869

C 0 0.616608 1.282684 -4.450073

C 0 0.445100 2.648041 -4.789551

C 0 0.136719 3.586533 -3.827316

C 0 -0.032516 3.225662 -2.449600

C 0 -0.054169 1.788345 -2.084259

C 0 0.321350 0.888855 -3.167100

N 0 -0.114227 4.227859 -1.531296

C 0 -0.167816 5.640961 -1.949800

C 0 0.008743 4.046204 -0.079483

C 0 -2.295563 2.247452 -0.708084

C 0 -2.671692 2.352417 0.643616

C 0 -2.959946 3.086655 -1.627258

C 0 -3.652466 3.261917 1.061905

C 0 -3.937119 3.997345 -1.216415

C 0 -4.289398 4.091904 0.135712

C 0 -2.183212 0.279404 -2.167068

H 0 -1.576492 -5.682449 0.740143

H 0 -1.680100 -4.004944 0.185776

H 0 -2.655000 -4.552628 1.555679

H 0 -1.923859 -4.075806 3.704193

H 0 2.176804 -3.150528 4.570206

H 0 3.835602 -3.239304 2.939117

H 0 3.303528 -2.772873 1.315521

H 0 3.625809 -4.466949 1.687714

H 0 0.460739 -6.394318 0.266556

H 0 2.172745 -6.206314 0.659119

H 0 1.509598 -3.869278 -3.980972

H 0 0.312454 -2.861084 -3.131714

H 0 -0.838500 -4.751938 -2.202866

H 0 -0.618958 -5.182785 -3.895966

H 0 3.463730 -4.792084 -1.166290

H 0 3.087738 -5.078918 -2.876480

H 0 2.354797 -7.100952 -1.467316

H 0 -1.304428 -2.988998 5.861740

H 0 0.390503 -2.616928 6.222366

H 0 -0.197647 -4.277191 6.344131

H 0 1.115173 -6.762024 -3.426697

H 0 -0.093613 -7.050110 -2.186050

H 0 -0.233963 0.816238 0.675003

H 0 -1.701324 -0.115570 0.474152

H 0 0.903839 0.560638 -5.206284

H 0 0.574890 2.968796 -5.818680

H 0 0.044067 4.621200 -4.125473

H 0 0.354889 -0.164520 -2.909470

H 0 -0.344162 6.254318 -1.066284

H 0 0.773239 5.958710 -2.414185

H 0 -0.992798 5.812698 -2.646866

H 0 -0.876053 4.430038 0.431854

H 0 0.109482 2.995056 0.170883

H 0 0.895645 4.590149 0.270309

H 0 -2.199800 1.722107 1.386566

H 0 -2.715561 3.031311 -2.683441

H 0 -3.920349 3.312347 2.112778

H 0 -4.431198 4.624100 -1.952591

H 0 -5.052994 4.792633 0.457077

H 0 -1.659515 -0.624969 -2.482941

H 0 -3.077148 -0.033600 -1.617432

H 0 -2.507172 0.830551 -3.054489

H 0 1.205780 1.694016 -1.359970

C 0 4.410110 5.363403 -1.805252

C 0 4.273666 4.966385 -0.472565

C 0 3.658768 3.749710 -0.150070

C 0 3.171188 2.917557 -1.170074

C 0 3.305283 3.318451 -2.509811

C 0 3.921890 4.533524 -2.821167

N 0 2.508911 1.633194 -0.874969

C 0 2.475128 1.279556 0.579834

C 0 3.130737 0.487747 -1.635605

H 0 4.894379 6.302246 -2.050034

H 0 4.654221 5.594910 0.325378

H 0 3.586151 3.465363 0.890762

H 0 2.927078 2.697510 -3.311844

H 0 4.023560 4.826416 -3.860703

H 0 1.928238 2.036575 1.140747

H 0 1.962570 0.324280 0.680786

H 0 3.493927 1.196564 0.973541

H 0 3.079391 0.676636 -2.705185

H 0 4.176468 0.374878 -1.331757

H 0 2.569641 -0.420013 -1.406570

Int2_C_

Au 0 -0.314240 -1.391403 -0.187836

C 0 0.326859 -3.740967 2.659653

C 0 2.739105 -3.070786 2.962036

C 0 4.304153 -2.992386 1.134125

C 0 4.040176 -2.819748 2.501297

C 0 2.017929 -3.674255 0.738815

C 0 3.649460 -3.621948 -1.223740

C 0 1.713684 -3.503357 2.106262

C 0 3.313736 -3.423477 0.238846

N 0 -0.529709 -3.910889 -1.869949

N 0 1.013900 -4.177109 -0.183487

C 0 0.898956 -5.683243 -0.227905

C 0 -2.017044 -3.900223 -1.805161

C 0 -2.402710 -4.934525 -0.717468

C 0 -0.019775 -5.191330 -2.418152

C 0 -0.129715 -6.190979 -1.262512

C 0 0.133667 -3.349625 -0.799652

C 0 5.137054 -2.413513 3.460281

C 0 -1.631653 -6.282181 -0.843002

C 0 -0.861389 0.564240 0.419235

C 0 -2.386322 0.756622 0.722336

C 0 -4.969955 -1.221115 -1.384247

C 0 -4.819107 -0.671982 -2.658432

C 0 -3.881317 0.346069 -2.844940

C 0 -3.087891 0.823730 -1.788818

C 0 -3.226318 0.270462 -0.487213

C 0 -4.184311 -0.750839 -0.325531

N 0 -2.138321 1.895828 -2.070496

C 0 -1.152542 1.558548 -3.115082

C 0 -2.770859 3.212600 -2.305786

C 0 -2.754898 2.232727 1.052734

C 0 -4.103241 2.640945 1.014450

C 0 -1.806305 3.168457 1.498566

C 0 -4.483719 3.934296 1.387604

C 0 -2.181793 4.466202 1.870667

C 0 -3.522308 4.858032 1.814270

C 0 -2.707077 -0.042267 2.020828

H 0 -0.070709 -4.725311 2.389145

H 0 -0.379227 -2.991470 2.281693

H 0 0.333069 -3.677032 3.751068

H 0 2.514877 -2.939987 4.016846

H 0 5.307419 -2.810196 0.758286

H 0 4.665588 -3.278122 -1.437225

H 0 2.959030 -3.082565 -1.882553

H 0 3.600357 -4.679657 -1.512314

H 0 0.654495 -6.015991 0.786316

H 0 1.888443 -6.081039 -0.476151

H 0 -2.405060 -4.164841 -2.792831

H 0 -2.370483 -2.899658 -1.546967

H 0 -2.192215 -4.477463 0.258774

H 0 -3.480453 -5.133575 -0.746552

H 0 1.013031 -5.053848 -2.751740

H 0 -0.630890 -5.462280 -3.281296

H 0 0.188049 -7.196594 -1.563751

H 0 4.750763 -1.796800 4.278519

H 0 5.929794 -1.853531 2.953644

H 0 5.605408 -3.296005 3.915588

H 0 -2.124847 -6.895767 -1.609940

H 0 -1.722626 -6.839142 0.097534

H 0 -0.631470 1.287766 -0.376816

H 0 -0.300674 0.831848 1.329056

H 0 -5.703003 -2.001511 -1.204269

H 0 -5.427475 -1.016647 -3.488388

H 0 -3.766083 0.789673 -3.829391

H 0 -4.332733 -1.191360 0.651443

H 0 -0.686661 0.597076 -2.877914

H 0 -1.582596 1.490784 -4.130402

H 0 -0.380478 2.338120 -3.139511

H 0 -3.440659 3.450500 -1.478836

H 0 -1.990555 3.980484 -2.351608

H 0 -3.343735 3.250824 -3.249496

H 0 -4.863754 1.940200 0.685562

H 0 -0.762283 2.887589 1.570221

H 0 -5.530746 4.218491 1.346741

H 0 -1.425323 5.166275 2.213654

H 0 -3.815292 5.861633 2.104706

H 0 -2.484070 -1.108032 1.899368

H 0 -2.081680 0.343277 2.832764

H 0 -3.751144 0.074249 2.328476

C 0 2.949036 5.449066 -0.798477

C 0 1.635422 4.969864 -0.768585

C 0 1.384888 3.600632 -0.610657

C 0 2.469955 2.731537 -0.480606

C 0 3.789444 3.190460 -0.510100

C 0 4.023712 4.559830 -0.670258

N 0 2.201309 1.267624 -0.317047

C 0 2.756195 0.701675 0.982117

C 0 2.671738 0.458084 -1.515121

H 0 3.136902 6.509811 -0.920654

H 0 0.800186 5.654037 -0.864563

H 0 0.363190 3.235016 -0.584518

H 0 4.627884 2.509552 -0.411041

H 0 5.042800 4.928635 -0.693710

H 0 1.174103 1.133011 -0.262268

H 0 2.390564 -0.319977 1.089661

H 0 3.844452 0.717682 0.939667

H 0 2.399689 1.322144 1.803574

H 0 2.341583 -0.571655 -1.374161

H 0 2.219100 0.880295 -2.411789

H 0 3.758087 0.518800 -1.574585

TS3_C_

Au 0 1.604416 -0.386826 -0.297882

C 0 4.180603 0.548828 2.510574

C 0 3.907166 2.991562 1.943741

C 0 4.003220 3.817245 -0.316025

C 0 3.850433 4.074326 1.054672

C 0 4.288284 1.461380 0.119949

C 0 4.377136 2.287552 -2.292984

C 0 4.130035 1.677948 1.504517

C 0 4.224197 2.522751 -0.806335

N 0 3.962036 -1.821991 -1.472717

N 0 4.565979 0.123062 -0.380539

C 0 6.007767 -0.311386 -0.270920

C 0 3.615097 -3.146118 -0.883652

C 0 4.532410 -3.320587 0.352097

C 0 5.329437 -1.822769 -2.050217

C 0 6.272217 -1.719437 -0.846603

C 0 3.576279 -0.712921 -0.754944

C 0 3.657928 5.485443 1.562759

C 0 6.020752 -2.955750 0.074051

H 0 5.209686 0.225906 2.716965

H 0 3.620560 -0.326752 2.166290

H 0 3.750946 0.867950 3.464172

H 0 3.778885 3.170105 3.007553

H 0 3.956848 4.642532 -1.020874

H 0 4.333298 3.234177 -2.838089

H 0 3.585327 1.637314 -2.684418

H 0 5.334991 1.812119 -2.538086

H 0 6.279068 -0.247787 0.787277

H 0 6.609100 0.425629 -0.813034

H 0 3.786575 -3.911179 -1.645966

H 0 2.559860 -3.162949 -0.602264

H 0 4.135513 -2.678513 1.149811

H 0 4.477081 -4.350876 0.722214

H 0 5.429489 -0.974747 -2.734070

H 0 5.458649 -2.745438 -2.619019

H 0 7.324219 -1.746933 -1.155014

H 0 3.076508 5.503235 2.490128

H 0 3.145400 6.112274 0.825912

H 0 4.624344 5.960983 1.776703

H 0 6.502518 -3.809800 -0.422089

H 0 6.543991 -2.822128 1.028610

C 0 -0.638977 0.010529 0.169785

C 0 -1.077423 -1.049973 1.222794

C 0 0.503754 -4.587189 1.381989

C 0 0.261810 -5.109634 0.110626

C 0 -0.427032 -4.324356 -0.815109

C 0 -0.876862 -3.028305 -0.505768

C 0 -0.622375 -2.474319 0.780457

C 0 0.064453 -3.298233 1.698639

N 0 -1.617004 -2.306473 -1.542313

C 0 -0.865829 -2.153778 -2.811188

C 0 -2.967422 -2.873900 -1.786072

C 0 -2.603943 -1.041840 1.539978

C 0 -3.349976 -2.224747 1.687531

C 0 -3.247864 0.171478 1.845139

C 0 -4.694061 -2.191238 2.084106

C 0 -4.586075 0.211700 2.245163

C 0 -5.321350 -0.973480 2.359909

C 0 -0.397217 -0.639313 2.570419

H 0 -0.722061 -0.269424 -0.882675

H 0 -0.126907 0.908524 0.542572

H 0 1.019089 -5.177933 2.132660

H 0 0.583954 -6.112274 -0.151184

H 0 -0.638138 -4.729400 -1.799881

H 0 0.261765 -2.932907 2.696655

H 0 0.125046 -1.738846 -2.605942

H 0 -0.735977 -3.099823 -3.363678

H 0 -1.411453 -1.463181 -3.464157

H 0 -3.544968 -2.863464 -0.860779

H 0 -3.482483 -2.257294 -2.530106

H 0 -2.930283 -3.907074 -2.171936

H 0 -2.881775 -3.185165 1.508300

H 0 -2.688873 1.099411 1.790451

H 0 -5.243271 -3.121994 2.187408

H 0 -5.053207 1.165390 2.470566

H 0 -6.360413 -0.947540 2.670837

H 0 0.694031 -0.708145 2.503433

H 0 -0.656647 0.399078 2.798798

H 0 -0.747620 -1.248627 3.407974

C 0 -6.281372 1.397079 -1.752426

C 0 -5.350815 0.351974 -1.808990

C 0 -4.007462 0.586624 -1.506119

C 0 -3.596359 1.874908 -1.145325

C 0 -4.516815 2.926910 -1.086853

C 0 -5.862442 2.680328 -1.388672

N 0 -2.160538 2.078384 -0.864822

C 0 -1.854294 3.229202 0.038513

C 0 -1.366882 2.187302 -2.141052

H 0 -7.324219 1.211136 -1.984741

H 0 -5.673019 -0.649139 -2.073730

H 0 -3.287003 -0.223785 -1.512833

H 0 -4.209793 3.929382 -0.813477

H 0 -6.577377 3.494659 -1.341583

H 0 -0.809036 3.155609 0.348800

H 0 -2.002014 4.185913 -0.471329

H 0 -2.497543 3.183105 0.917343

H 0 -0.304474 2.250198 -1.894272

H 0 -1.548660 1.300018 -2.747818

H 0 -1.674271 3.076904 -2.699554

H 0 -1.627121 1.018585 -0.269470

Pro_C_

Au 0 2.126083 -1.858093 -1.103806

C 0 5.705109 -2.908875 -2.576172

C 0 4.771057 -5.225784 -2.912033

C 0 3.529724 -6.388611 -1.207855

C 0 4.077484 -6.371887 -2.499908

C 0 4.382187 -4.173264 -0.779160

C 0 3.058960 -5.363129 1.052826

C 0 4.943054 -4.115372 -2.071625

C 0 3.670013 -5.305969 -0.330154

N 0 3.747437 -1.257736 1.345215

N 0 4.557709 -3.058456 0.141830

C 0 5.906921 -2.979568 0.814651

C 0 3.910309 0.194336 1.060532

C 0 5.335068 0.362961 0.476303

C 0 4.632813 -1.703720 2.450012

C 0 6.026443 -1.814500 1.822159

C 0 3.632538 -2.088364 0.256607

C 0 3.940063 -7.567917 -3.414063

C 0 6.425995 -0.405014 1.280700

C 0 0.474411 -1.695404 -2.665070

C 0 0.545425 -0.302155 -2.967178

C 0 -0.325851 0.576019 -2.359802

C 0 -1.338211 0.152603 -1.435654

C 0 -1.566330 -1.275055 -1.271439

C 0 -0.634216 -2.124619 -1.871109

N 0 -2.049759 1.145569 -0.798782

C 0 -2.156097 2.493042 -1.382431

C 0 -2.428940 1.107635 0.620682

C 0 -2.437408 -2.437622 0.843964

C 0 -2.800583 -1.967972 -0.594437

C 0 -4.095352 -1.127213 -0.669937

C 0 -4.480316 -0.549530 -1.895050

C 0 -4.993912 -1.045822 0.407120

C 0 -5.709930 0.098450 -2.035873

C 0 -6.230728 -0.400574 0.268753

C 0 -6.594650 0.175247 -0.952057

C 0 -3.168625 -3.282532 -1.376938

H 0 6.763214 -2.942000 -2.283234

H 0 5.284058 -1.970291 -2.203049

H 0 5.681442 -2.872971 -3.668991

H 0 5.189468 -5.189850 -3.913773

H 0 2.981995 -7.264786 -0.873581

H 0 2.602036 -6.340179 1.229797

H 0 2.280579 -4.601852 1.185349

H 0 3.806808 -5.202347 1.839294

H 0 6.653427 -2.904907 0.018341

H 0 6.063660 -3.931763 1.331329

H 0 3.770767 0.742477 1.996140

H 0 3.145309 0.514038 0.347733

H 0 5.313019 -0.001648 -0.559723

H 0 5.602890 1.424988 0.430038

H 0 4.268951 -2.660385 2.835571

H 0 4.579987 -0.962845 3.249969

H 0 6.779465 -2.108246 2.563049

H 0 3.999664 -7.277176 -4.467361

H 0 2.988556 -8.085983 -3.257004

H 0 4.740677 -8.296707 -3.230500

H 0 6.697983 0.195282 2.160034

H 0 7.334198 -0.482376 0.671173

H 0 0.921448 -2.414749 -3.350708

H 0 1.295547 0.065186 -3.660080

H 0 -0.207733 1.634811 -2.544937

H 0 -0.775665 -3.193924 -1.788803

H 0 -1.289063 3.126144 -1.141006

H 0 -2.268280 2.434128 -2.466812

H 0 -3.049347 2.971191 -0.972778

H 0 -3.500183 1.268585 0.752060

H 0 -2.145706 0.159561 1.064453

H 0 -1.890091 1.906494 1.149628

H 0 -1.507294 -3.014343 0.809128

H 0 -2.294281 -1.620682 1.555298

H 0 -3.218277 -3.093430 1.242645

H 0 -3.813187 -0.610977 -2.749725

H 0 -4.743484 -1.487915 1.364655

H 0 -5.983017 0.532715 -2.992523

H 0 -6.910339 -0.359192 1.114243

H 0 -7.555496 0.666916 -1.062088

H 0 -2.433472 -4.079788 -1.226379

H 0 -4.127060 -3.651764 -1.001556

H 0 -3.274597 -3.093765 -2.448944

C 0 -5.515518 3.959213 -0.001144

C 0 -5.553787 3.577515 1.346497

C 0 -5.161224 4.455582 2.357712

C 0 -4.712800 5.765732 2.047318

C 0 -4.677689 6.142776 0.679810

C 0 -5.074478 5.250961 -0.317612

N 0 -4.325150 6.650391 3.048447

C 0 -4.430664 6.260513 4.453125

C 0 -3.921417 8.013535 2.706757

H 0 -5.841507 3.274673 -0.776978

H 0 -5.907181 2.585953 1.615509

H 0 -5.216537 4.128326 3.388153

H 0 -4.353531 7.135910 0.396271

H 0 -5.050446 5.578094 -1.353348

H 0 -3.810028 5.381943 4.678055

H 0 -4.085098 7.083313 5.080414

H 0 -5.465881 6.025833 4.740341

H 0 -3.047668 8.021851 2.040787

H 0 -4.729446 8.571869 2.212418

H 0 -3.651703 8.546768 3.619293

Rea_D_

Au 0 0.560944 0.882874 -0.636536

C 0 4.170700 1.543747 -1.157913

C 0 4.266022 3.006409 0.887726

C 0 2.661682 4.747803 1.330673

C 0 3.800262 4.026733 1.726974

C 0 2.507675 3.450867 -0.696304

C 0 0.776428 5.280640 -0.264832

C 0 3.640503 2.695557 -0.330750

C 0 2.001724 4.482666 0.125015

N 0 0.144700 2.298096 -3.256882

N 0 1.846054 3.202896 -1.972717

C 0 2.445953 3.920471 -3.159805

C 0 0.188889 1.064500 -4.093857

C 0 1.612457 1.002029 -4.700684

C 0 0.228531 3.527512 -4.087463

C 0 1.704697 3.641556 -4.485504

C 0 0.884537 2.277405 -2.101166

C 0 4.518753 4.363953 3.013428

C 0 2.090698 2.360748 -5.292145

H 0 3.925705 1.632690 -2.219635

H 0 3.757141 0.589371 -0.805511

H 0 5.259232 1.477020 -1.069550

H 0 5.140289 2.433243 1.182480

H 0 2.281876 5.537582 1.972092

H 0 0.528458 6.006104 0.514008

H 0 -0.099594 4.637527 -0.412415

H 0 0.927917 5.842239 -1.195618

H 0 3.497360 3.618729 -3.203857

H 0 2.416687 4.991302 -2.936462

H 0 -0.592072 1.143738 -4.854584

H 0 -0.019104 0.191910 -3.468094

H 0 2.298538 0.680649 -3.905533

H 0 1.655777 0.234467 -5.481888

H 0 -0.113617 4.380844 -3.495316

H 0 -0.438629 3.405884 -4.942535

H 0 1.877640 4.506180 -5.136887

H 0 5.101685 3.514771 3.382172

H 0 3.817245 4.663284 3.798920

H 0 5.215332 5.199707 2.865524

H 0 1.649979 2.481888 -6.291306

H 0 3.176514 2.325836 -5.440109

C 0 0.479736 -0.057953 1.534103

C 0 0.262482 -1.107025 0.672180

C 0 1.392944 -2.010100 0.240875

C 0 1.020889 -3.419128 -0.130493

O 0 2.564774 -1.591309 0.215500

H 0 -0.353012 0.473022 1.984543

H 0 1.476761 0.153183 1.911850

H 0 -0.752502 -1.423386 0.440979

H 0 1.907410 -3.966766 -0.452194

H 0 0.567352 -3.935776 0.725600

H 0 0.271225 -3.417236 -0.931763

C 0 -2.634094 -0.998047 -1.874329

C 0 -2.867859 -2.223831 -1.236649

C 0 -3.334106 -2.276703 0.079193

C 0 -3.600845 -1.084122 0.807312

C 0 -3.353043 0.153000 0.151917

C 0 -2.883636 0.184906 -1.163971

N 0 -4.089340 -1.127686 2.104600

C 0 -4.433823 0.112167 2.802460

C 0 -4.407913 -2.414368 2.728607

H 0 -2.306152 -0.966507 -2.907715

H 0 -2.709351 -3.154358 -1.774231

H 0 -3.525726 -3.243134 0.527313

H 0 -3.561325 1.088272 0.655685

H 0 -2.730225 1.147247 -1.643768

H 0 -5.214447 0.678528 2.275803

H 0 -4.804900 -0.129150 3.798767

H 0 -3.559341 0.767258 2.919830

H 0 -5.186234 -2.958328 2.175781

H 0 -3.522400 -3.059830 2.796600

H 0 -4.771591 -2.240128 3.741516

TS1_D_

Au 0 -2.060944 -0.733643 -0.432068

C 0 -5.521484 0.363693 -2.089400

C 0 -5.402451 2.476379 -0.716888

C 0 -4.909866 2.468842 1.640182

C 0 -5.217850 3.189362 0.476669

C 0 -5.001343 0.389297 0.421112

C 0 -4.455017 0.334442 2.914413

C 0 -5.306244 1.077652 -0.772186

C 0 -4.797500 1.071854 1.638580

N 0 -3.728180 -3.042084 0.536500

N 0 -4.920517 -1.064697 0.422012

C 0 -6.245255 -1.787354 0.459290

C 0 -3.362473 -3.961517 -0.576569

C 0 -4.558000 -3.947525 -1.561707

C 0 -4.854294 -3.578125 1.342361

C 0 -6.107162 -3.325607 0.496750

C 0 -3.747925 -1.699265 0.238403

C 0 -5.370544 4.692917 0.512756

C 0 -5.939011 -4.087097 -0.856720

C 0 0.701721 -0.682037 -0.964188

C 0 -0.263596 0.371628 -1.129883

C 0 -0.582245 0.789108 -2.525620

C 0 -1.414230 2.037582 -2.722717

O 0 -0.134323 0.147888 -3.505478

H 0 -6.530212 -0.064285 -2.164124

H 0 -4.803665 -0.449203 -2.237946

H 0 -5.411652 1.061203 -2.924179

H 0 -5.631042 3.018616 -1.629990

H 0 -4.754990 3.004440 2.572174

H 0 -4.398636 1.029861 3.755829

H 0 -3.488235 -0.178177 2.839813

H 0 -5.205917 -0.425186 3.164230

H 0 -6.812744 -1.452637 -0.414154

H 0 -6.775238 -1.443161 1.353241

H 0 -3.176453 -4.952240 -0.153152

H 0 -2.447021 -3.606216 -1.056976

H 0 -4.518951 -3.000229 -2.116089

H 0 -4.448486 -4.748611 -2.301941

H 0 -4.884888 -3.056671 2.303421

H 0 -4.667267 -4.637650 1.527359

H 0 -7.008057 -3.716125 0.984741

H 0 -5.081146 5.149231 -0.439209

H 0 -4.760712 5.139633 1.304184

H 0 -6.413635 4.976883 0.705400

H 0 -6.112213 -5.149216 -0.634216

H 0 -6.730804 -3.788467 -1.554153

H 0 0.658981 -1.496521 -1.679825

H 0 1.032806 -0.945953 0.034698

H 0 -0.225220 1.193207 -0.410904

H 0 -1.763733 2.084152 -3.755829

H 0 -2.267212 2.071213 -2.036789

H 0 -0.801117 2.928391 -2.527664

C 0 2.595184 -0.087219 -1.701492

C 0 2.978119 0.938492 -0.783798

C 0 3.941254 0.731689 0.180359

C 0 4.638718 -0.517090 0.261719

C 0 4.307144 -1.530441 -0.695160

C 0 3.345230 -1.303100 -1.655563

N 0 5.592789 -0.731552 1.214661

C 0 6.314468 -2.013626 1.279617

C 0 5.945114 0.327026 2.175461

H 0 2.092163 0.173584 -2.629730

H 0 2.478485 1.901245 -0.831451

H 0 4.180008 1.530670 0.869034

H 0 4.833100 -2.475327 -0.686615

H 0 3.136581 -2.075516 -2.389893

H 0 6.890000 -2.195496 0.364502

H 0 7.008072 -1.988724 2.118851

H 0 5.622787 -2.849869 1.432465

H 0 6.349274 1.209976 1.666779

H 0 5.074417 0.629913 2.768311

H 0 6.705231 -0.050980 2.857635

Int1_D_

Au 0 -1.263840 -2.194946 -0.438660

C 0 -5.136932 -1.202652 -0.453537

C 0 -4.783981 0.031326 1.717896

C 0 -3.592331 -0.928970 3.576065

C 0 -4.298355 0.151764 3.027359

C 0 -3.892426 -2.202148 1.548691

C 0 -2.602615 -3.252335 3.483582

C 0 -4.598663 -1.133835 0.958862

C 0 -3.378311 -2.113739 2.859055

N 0 -2.417389 -4.975723 -0.333725

N 0 -3.712814 -3.448822 0.819275

C 0 -4.907959 -4.369095 0.822266

C 0 -2.353149 -5.203079 -1.803131

C 0 -3.792542 -4.997849 -2.338196

C 0 -3.200500 -6.034012 0.349884

C 0 -4.667023 -5.694290 0.066116

C 0 -2.613266 -3.671646 0.070755

C 0 -4.547287 1.405197 3.835602

C 0 -4.878006 -5.714523 -1.481216

C 0 1.623062 -1.269363 -0.636612

C 0 0.235367 -0.686844 -0.904785

C 0 -0.043732 -0.290024 -2.289200

C 0 -1.281464 0.535141 -2.587097

O 0 0.727142 -0.600739 -3.253113

C 0 2.812561 -0.472244 -1.347504

C 0 2.918228 0.949234 -0.986100

C 0 4.101288 1.575439 -0.732758

C 0 5.341904 0.842728 -0.769012

C 0 5.288239 -0.574844 -1.045044

C 0 4.099686 -1.184158 -1.311218

N 0 6.518814 1.461670 -0.549805

C 0 6.577988 2.921661 -0.309800

C 0 7.790375 0.706223 -0.540253

H 0 -6.059265 -1.795380 -0.516769

H 0 -4.409882 -1.644806 -1.142319

H 0 -5.378372 -0.200455 -0.818573

H 0 -5.320068 0.863831 1.271118

H 0 -3.199524 -0.850906 4.585709

H 0 -2.331207 -3.011719 4.514862

H 0 -1.677002 -3.460266 2.933334

H 0 -3.182968 -4.183243 3.503723

H 0 -5.744537 -3.806839 0.396591

H 0 -5.149521 -4.584000 1.868469

H 0 -1.979782 -6.216263 -1.977097

H 0 -1.656631 -4.489304 -2.250595

H 0 -3.987640 -3.917160 -2.353912

H 0 -3.865646 -5.345474 -3.375504

H 0 -2.967377 -6.016357 1.418533

H 0 -2.896759 -7.000870 -0.056351

H 0 -5.345932 -6.438324 0.500122

H 0 -4.663269 2.281921 3.190643

H 0 -3.727020 1.601974 4.533478

H 0 -5.465729 1.314392 4.430862

H 0 -4.908737 -6.773956 -1.771927

H 0 -5.863266 -5.301224 -1.729034

H 0 1.697540 -2.289474 -1.029144

H 0 1.813919 -1.314529 0.440170

H 0 -0.048523 0.104156 -0.199800

H 0 -1.940002 -0.019592 -3.264313

H 0 -1.840118 0.787247 -1.682495

H 0 -0.991074 1.455811 -3.106323

H 0 2.403091 -0.521179 -2.400681

H 0 2.004272 1.532288 -0.957016

H 0 4.101212 2.636032 -0.522873

H 0 6.200638 -1.155441 -1.054535

H 0 4.087600 -2.248900 -1.529587

H 0 7.620224 3.235962 -0.313629

H 0 6.046600 3.463043 -1.097290

H 0 6.139191 3.177292 0.660600

H 0 7.774429 -0.077988 0.222672

H 0 7.984589 0.252930 -1.517654

H 0 8.604736 1.390320 -0.309784

C 0 3.122726 5.178284 -1.804779

C 0 4.247925 5.085190 -2.636017

C 0 4.246643 4.267731 -3.768768

C 0 3.097504 3.508972 -4.118240

C 0 1.959335 3.620377 -3.275223

C 0 1.980591 4.441223 -2.145584

N 0 3.083160 2.699173 -5.244583

C 0 4.234573 2.681152 -6.146103

C 0 1.867432 1.967178 -5.616104

H 0 3.122040 5.835632 -0.941910

H 0 5.131592 5.679993 -2.419144

H 0 5.128555 4.240051 -4.395813

H 0 1.051926 3.082184 -3.518570

H 0 1.084305 4.521240 -1.536407

H 0 5.143204 2.330780 -5.636963

H 0 4.031158 1.999039 -6.972137

H 0 4.444580 3.674377 -6.568481

H 0 1.557541 1.260666 -4.835968

H 0 1.028687 2.647568 -5.824051

H 0 2.063812 1.392349 -6.521973

TS2_D_

Au 0 0.269836 -2.742065 0.452560

C 0 3.181488 -4.160583 2.743912

C 0 4.855225 -2.834579 1.400040

C 0 4.869247 -2.826569 -1.007065

C 0 5.425659 -2.383408 0.202438

C 0 3.242249 -4.150940 0.184769

C 0 3.197739 -4.147720 -2.365738

C 0 3.766373 -3.719864 1.419891

C 0 3.780991 -3.707840 -1.041306

N 0 -0.070709 -5.669830 -0.188126

N 0 2.143097 -5.104980 0.152527

C 0 2.546631 -6.544769 0.350937

C 0 -1.123108 -5.909348 0.835861

C 0 -0.420334 -6.606277 2.027908

C 0 0.427002 -6.940674 -0.768875

C 0 1.359406 -7.530746 0.293777

C 0 0.858032 -4.694885 0.115997

C 0 6.619995 -1.455856 0.209000

C 0 0.525986 -7.764450 1.593994

C 0 -1.234741 -0.278000 -0.496400

C 0 -0.365845 -0.679825 0.698395

C 0 -1.007416 -0.654129 2.017761

C 0 -0.125397 -0.686493 3.257385

O 0 -2.263779 -0.608322 2.170059

C 0 -1.703659 1.234772 -0.570389

C 0 -1.043000 2.218903 0.274185

C 0 -0.865906 3.529770 -0.092773

C 0 -1.255539 3.989136 -1.395264

C 0 -1.794388 3.023500 -2.307358

C 0 -1.974640 1.720932 -1.919495

N 0 -1.109222 5.291519 -1.756897

C 0 -0.501450 6.268036 -0.834656

C 0 -1.552551 5.758148 -3.083572

H 0 3.432098 -5.202362 2.983536

H 0 2.089920 -4.072021 2.751938

H 0 3.573563 -3.543915 3.557373

H 0 5.263321 -2.489807 2.345795

H 0 5.290649 -2.479355 -1.946274

H 0 3.782104 -3.742325 -3.196075

H 0 2.163254 -3.802887 -2.483459

H 0 3.188690 -5.239716 -2.470383

H 0 3.073029 -6.599762 1.308624

H 0 3.265549 -6.792465 -0.437317

H 0 -1.903503 -6.530411 0.386780

H 0 -1.560776 -4.953690 1.135651

H 0 0.154648 -5.840317 2.565247

H 0 -1.163956 -6.994522 2.734024

H 0 0.947159 -6.725082 -1.706924

H 0 -0.432938 -7.577240 -0.987442

H 0 1.763977 -8.501831 -0.016525

H 0 6.752640 -0.980621 1.185532

H 0 6.517563 -0.665131 -0.542419

H 0 7.544952 -2.001236 -0.019958

H 0 -0.082062 -8.661453 1.410355

H 0 1.195511 -8.014084 2.426147

H 0 -2.123978 -0.914703 -0.533096

H 0 -0.666580 -0.471680 -1.412720

H 0 0.600769 -0.160645 0.711075

H 0 -0.412216 -1.531464 3.892166

H 0 0.936615 -0.768143 3.014008

H 0 -0.292831 0.223709 3.846695

H 0 -0.711868 1.903076 1.257339

H 0 -0.409622 4.217529 0.606567

H 0 -2.051376 3.316177 -3.316086

H 0 -2.359894 1.008575 -2.643921

H 0 -0.475708 7.243500 -1.317825

H 0 -1.084625 6.356918 0.088730

H 0 0.525574 5.985779 -0.578430

H 0 -0.951263 5.316147 -3.886307

H 0 -2.605957 5.508835 -3.250214

H 0 -1.445618 6.840759 -3.132568

H 0 -2.873642 1.191269 -0.085022

C 0 -6.156708 3.831268 -2.490143

C 0 -5.907288 2.486435 -2.784454

C 0 -5.343643 1.640823 -1.825562

C 0 -5.019791 2.126678 -0.542618

C 0 -5.283661 3.477585 -0.248520

C 0 -5.844360 4.317017 -1.216232

N 0 -4.365860 1.260712 0.416153

C 0 -4.861023 -0.151505 0.469131

C 0 -4.190674 1.810867 1.788330

H 0 -6.603210 4.482910 -3.233292

H 0 -6.156600 2.087540 -3.762405

H 0 -5.165298 0.604919 -2.084930

H 0 -5.069900 3.883057 0.731033

H 0 -6.051483 5.351746 -0.962753

H 0 -4.819153 -0.609833 -0.518280

H 0 -4.200119 -0.700287 1.138947

H 0 -5.896439 -0.179794 0.830841

H 0 -3.579681 2.715668 1.749359

H 0 -5.158539 2.047577 2.248032

H 0 -3.667068 1.060226 2.379608

Int2_D_

Au 0 1.203003 -0.659744 -0.087006

C 0 4.633392 -1.011505 -1.391846

C 0 5.634720 -1.218689 0.902771

C 0 5.120377 0.291702 2.705048

C 0 5.809952 -0.839279 2.238770

C 0 4.144440 0.636093 0.523666

C 0 3.555054 2.250870 2.411500

C 0 4.808090 -0.500961 0.021927

C 0 4.288361 1.043854 1.868210

N 0 1.297073 2.195084 -1.199478

N 0 3.325378 1.465637 -0.353577

C 0 4.101151 2.501328 -1.129349

C 0 0.694763 1.812607 -2.508743

C 0 1.857529 1.664780 -3.520264

C 0 1.907867 3.547684 -1.260971

C 0 3.216600 3.379852 -2.039307

C 0 2.025177 1.192032 -0.570236

C 0 6.736710 -1.611649 3.150375

C 0 2.872314 2.842667 -3.462921

C 0 0.502533 -2.657288 0.282883

C 0 -0.376343 -3.199982 -0.871689

C 0 -1.746796 -2.565186 -0.978363

C 0 -2.083191 -1.687531 -2.024948

C 0 -3.365173 -1.149368 -2.168976

C 0 -4.398773 -1.477554 -1.256744

C 0 -4.058716 -2.334290 -0.175537

C 0 -2.765945 -2.867661 -0.059540

N 0 -5.706284 -0.981476 -1.404892

C 0 -6.041214 -0.226608 -2.621033

C 0 -6.809128 -1.701248 -0.744385

C 0 1.789474 -3.395905 0.426056

C 0 2.399567 -3.493393 1.816360

O 0 2.367920 -3.929260 -0.552643

H 0 4.204910 -0.263474 -2.062881

H 0 3.966782 -1.883911 -1.401794

H 0 5.593964 -1.332413 -1.808640

H 0 6.147293 -2.100082 0.527985

H 0 5.236267 0.596130 3.741333

H 0 3.795090 2.401581 3.467529

H 0 2.467682 2.133362 2.326752

H 0 3.822754 3.173126 1.880142

H 0 4.860931 1.957474 -1.700745

H 0 4.623032 3.131119 -0.401627

H 0 -0.011154 2.596588 -2.801025

H 0 0.153687 0.869751 -2.390259

H 0 2.371384 0.720230 -3.298981

H 0 1.464218 1.576416 -4.540039

H 0 2.076675 3.908829 -0.241928

H 0 1.202393 4.219681 -1.755356

H 0 3.734436 4.338425 -2.165192

H 0 6.800156 -2.664459 2.858154

H 0 6.404175 -1.567184 4.192490

H 0 7.755005 -1.201752 3.115784

H 0 2.456650 3.692291 -4.023071

H 0 3.791992 2.552490 -3.984955

H 0 -0.045914 -2.672455 1.235718

H 0 -0.502731 -4.286102 -0.735200

H 0 0.163055 -3.086029 -1.816544

H 0 -1.330444 -1.448990 -2.771133

H 0 -3.571213 -0.520889 -3.026047

H 0 -4.819366 -2.658829 0.524506

H 0 -2.563980 -3.568893 0.746246

H 0 -7.069717 0.129181 -2.544708

H 0 -5.392792 0.649567 -2.722168

H 0 -5.949265 -0.831528 -3.535675

H 0 -6.836655 -2.764877 -1.020721

H 0 -6.732727 -1.632156 0.346558

H 0 -7.755005 -1.243637 -1.037354

H 0 3.472992 -3.678055 1.738037

H 0 2.223740 -2.586807 2.404495

H 0 1.945938 -4.338425 2.352768

C 0 -6.247635 2.560684 2.979828

C 0 -6.388504 1.793610 1.818420

C 0 -5.303711 1.065964 1.313065

C 0 -4.085709 1.120361 1.994125

C 0 -3.920853 1.888474 3.149170

C 0 -5.014755 2.608780 3.641327

N 0 -2.926239 0.360626 1.423813

C 0 -2.107468 -0.385590 2.463867

C 0 -2.033798 1.258530 0.572600

H 0 -7.091965 3.119324 3.367691

H 0 -7.340912 1.757706 1.301651

H 0 -5.414902 0.481277 0.401093

H 0 -2.971817 1.935272 3.670319

H 0 -4.900543 3.204132 4.540024

H 0 -3.314484 -0.372757 0.796463

H 0 -2.782257 -0.996200 3.062927

H 0 -1.384506 -1.006088 1.935440

H 0 -1.583481 0.332230 3.092636

H 0 -2.636429 1.661926 -0.240250

H 0 -1.660309 2.064072 1.204788

H 0 -1.203430 0.663437 0.186722

TS3_D_

Au 0 -2.528931 -0.428879 -0.613174

C 0 -2.102615 3.260910 -1.851944

C 0 -1.057800 3.822296 0.372986

C 0 -2.054214 3.194107 2.473419

C 0 -1.015930 3.828293 1.774506

C 0 -3.128754 2.590744 0.400864

C 0 -4.216812 1.894073 2.604492

C 0 -2.105300 3.219070 -0.338989

C 0 -3.120422 2.570740 1.811615

N 0 -5.437454 0.136780 -1.045959

N 0 -4.252960 1.974823 -0.292740

C 0 -5.337845 2.930130 -0.733414

C 0 -5.500320 -0.409180 -2.430038

C 0 -5.467880 0.810684 -3.384079

C 0 -6.667587 0.891098 -0.696869

C 0 -6.532486 2.234253 -1.422318

C 0 -4.237808 0.679642 -0.652161

C 0 0.103470 4.524506 2.514557

C 0 -6.435913 1.951828 -2.954605

H 0 -2.777481 4.035110 -2.241333

H 0 -2.405838 2.305054 -2.291214

H 0 -1.102097 3.496216 -2.224915

H 0 -0.258148 4.301147 -0.184479

H 0 -2.036819 3.185760 3.559235

H 0 -4.075700 2.067368 3.674591

H 0 -4.228806 0.809570 2.442566

H 0 -5.211044 2.274551 2.339691

H 0 -4.861572 3.664230 -1.390228

H 0 -5.682495 3.456741 0.161865

H 0 -6.420258 -0.992447 -2.524780

H 0 -4.645447 -1.069656 -2.599579

H 0 -4.436783 1.188690 -3.405869

H 0 -5.709717 0.497787 -4.406387

H 0 -6.719131 1.004272 0.389572

H 0 -7.529602 0.309174 -1.028458

H 0 -7.411270 2.869049 -1.258530

H 0 1.019791 4.557526 1.917100

H 0 0.331787 4.024277 3.461014

H 0 -0.168549 5.561523 2.752045

H 0 -7.451172 1.692856 -3.285461

H 0 -6.166443 2.871506 -3.487396

C 0 0.578659 -1.621979 0.560928

C 0 -0.428696 -1.535812 -0.640457

C 0 -0.021561 -2.333679 -1.852768

C 0 -0.094711 -1.671890 -3.210220

O 0 0.378860 -3.514404 -1.742493

C 0 1.897705 -0.930984 0.277573

C 0 2.081390 0.431671 0.567139

C 0 3.280975 1.086838 0.291580

C 0 4.369949 0.393188 -0.296494

C 0 4.184021 -0.982849 -0.587250

C 0 2.977386 -1.619736 -0.303223

N 0 5.571793 1.033173 -0.569122

C 0 5.749359 2.441300 -0.219818

C 0 6.685500 0.287186 -1.155319

H 0 0.758453 -2.674011 0.797226

H 0 0.103271 -1.161545 1.434982

H 0 -0.378250 -0.470932 -0.952484

H 0 -0.028885 -2.423203 -3.999435

H 0 -1.009918 -1.080719 -3.328369

H 0 0.755005 -0.982452 -3.312790

H 0 1.273666 0.994720 1.031158

H 0 3.375977 2.134583 0.546722

H 0 4.987106 -1.560883 -1.025604

H 0 2.873611 -2.675705 -0.535995

H 0 6.754333 2.756958 -0.502289

H 0 5.032425 3.085953 -0.747757

H 0 5.630997 2.614075 0.859436

H 0 7.017410 -0.533981 -0.504500

H 0 6.418350 -0.139252 -2.131683

H 0 7.529602 0.961731 -1.303680

C 0 -4.724335 -2.248520 3.425613

C 0 -3.339600 -2.062805 3.529068

C 0 -2.491364 -2.499954 2.509003

C 0 -3.018997 -3.137405 1.372620

C 0 -4.405441 -3.313843 1.263687

C 0 -5.250153 -2.869781 2.289841

N 0 -2.080811 -3.587479 0.332748

C 0 -2.729614 -4.103348 -0.911911

C 0 -1.103516 -4.619736 0.845825

H 0 -5.381302 -1.920700 4.223846

H 0 -2.916748 -1.585052 4.406403

H 0 -1.421997 -2.351624 2.602081

H 0 -4.841858 -3.802185 0.402695

H 0 -6.319916 -3.026779 2.200668

H 0 -3.343048 -3.316483 -1.357773

H 0 -1.940231 -4.387390 -1.606216

H 0 -3.352386 -4.978271 -0.699554

H 0 -0.654633 -4.274673 1.775330

H 0 -1.630997 -5.561523 1.033997

H 0 -0.329865 -4.752380 0.090027

H 0 -1.407227 -2.434326 -0.128220

Pro_D_

Au 0 -1.573608 -0.554047 0.050079

C 0 -4.840454 -2.587875 -0.886917

C 0 -5.512711 -0.680649 -2.392960

C 0 -5.455429 1.618027 -1.678665

C 0 -5.727646 0.674866 -2.680328

C 0 -4.790756 -0.130630 -0.159592

C 0 -4.684692 2.301895 0.625793

C 0 -5.051834 -1.110657 -1.140305

C 0 -4.987076 1.243652 -0.411362

N 0 -2.726700 -0.743118 2.810608

N 0 -4.342346 -0.538483 1.166122

C 0 -5.434494 -1.010956 2.094772

C 0 -1.981567 -1.969100 3.207413

C 0 -2.978134 -3.147873 3.079819

C 0 -3.793747 -0.409760 3.787659

C 0 -4.925461 -1.401733 3.499603

C 0 -3.036804 -0.622314 1.474213

C 0 -6.261810 1.108917 -4.026428

C 0 -4.370010 -2.846176 3.709091

C 0 1.121948 3.479660 0.819092

C 0 1.351120 2.991684 -0.626083

C 0 1.219406 1.491211 -0.804764

C 0 0.147827 0.926636 -1.485123

C 0 -0.009933 -0.495361 -1.620834

C 0 1.047500 -1.370697 -1.160782

C 0 2.127991 -0.771851 -0.450439

C 0 2.201614 0.604553 -0.296158

N 0 1.015564 -2.715363 -1.414246

C 0 -0.140747 -3.310211 -2.087875

C 0 2.069200 -3.599152 -0.890533

C 0 -0.312256 3.362991 1.309952

C 0 -0.561142 3.671021 2.769104

O 0 -1.238815 3.032364 0.546707

H 0 -5.648407 -3.024551 -0.284607

H 0 -3.897095 -2.780533 -0.364929

H 0 -4.819351 -3.136551 -1.832703

H 0 -5.710144 -1.424332 -3.159637

H 0 -5.609955 2.673065 -1.884964

H 0 -5.151428 3.250641 0.346313

H 0 -3.605545 2.480530 0.716049

H 0 -5.062149 2.027588 1.617828

H 0 -5.934143 -1.847046 1.596405

H 0 -6.158218 -0.193909 2.179443

H 0 -1.621597 -1.832581 4.230927

H 0 -1.120621 -2.099335 2.546234

H 0 -3.095673 -3.369598 2.010452

H 0 -2.560669 -4.049194 3.544098

H 0 -4.100250 0.629608 3.639908

H 0 -3.379517 -0.511581 4.792725

H 0 -5.764420 -1.267715 4.192780

H 0 -6.073029 0.354065 -4.795822

H 0 -5.806961 2.049652 -4.353668

H 0 -7.347076 1.270706 -3.985367

H 0 -4.301056 -2.994278 4.795807

H 0 -5.097046 -3.582367 3.345840

H 0 1.775558 2.943726 1.522980

H 0 1.411026 4.539078 0.902313

H 0 2.355728 3.309448 -0.934448

H 0 0.640472 3.501068 -1.284988

H 0 -0.606949 1.568237 -1.927521

H 0 -0.662476 -0.843750 -2.417480

H 0 2.936722 -1.379715 -0.067474

H 0 3.069427 1.010330 0.218964

H 0 -1.067184 -3.119308 -1.524033

H 0 -0.268768 -2.910660 -3.101196

H 0 0.003784 -4.387268 -2.165115

H 0 3.061554 -3.262177 -1.204712

H 0 2.053268 -3.643341 0.206741

H 0 1.911667 -4.604782 -1.279861

H 0 -0.070862 4.604782 3.067352

H 0 -1.633118 3.743500 2.961105

H 0 -0.140945 2.872772 3.395615

C 0 5.901077 -2.957611 -0.537354

C 0 6.118088 -1.854568 -1.372910

C 0 6.103668 -0.553421 -0.867661

C 0 5.865036 -0.311447 0.508484

C 0 5.649414 -1.434402 1.347260

C 0 5.668900 -2.730331 0.824966

N 0 5.843445 0.991806 1.021683

C 0 6.302185 2.101776 0.180496

C 0 5.818008 1.191574 2.473129

H 0 5.942474 -3.967392 -0.931549

H 0 6.317108 -2.005234 -2.429672

H 0 6.292175 0.270508 -1.543457

H 0 5.490005 -1.301987 2.409698

H 0 5.521300 -3.569916 1.498215

H 0 5.679459 2.201431 -0.716202

H 0 6.224304 3.032379 0.745010

H 0 7.347092 1.981400 -0.143158

H 0 4.919937 0.745834 2.916840

H 0 6.696121 0.757965 2.975723

H 0 5.797333 2.261871 2.685043

Rea_F_

Au 0 0.290359 -1.188354 -0.053085

C 0 0.297394 -3.779251 2.597580

C 0 2.686371 -3.258087 3.192780

C 0 4.433900 -3.056778 1.547089

C 0 4.030823 -3.004807 2.891800

C 0 2.191696 -3.623611 0.864655

C 0 4.010162 -3.410721 -0.918427

C 0 1.743240 -3.570633 2.200089

C 0 3.536911 -3.367279 0.518295

N 0 -0.128586 -3.463394 -1.974319

N 0 1.266068 -3.989273 -0.202103

C 0 1.091949 -5.474045 -0.423600

C 0 -1.617645 -3.380035 -1.971619

C 0 -2.113373 -4.494385 -1.017105

C 0 0.349274 -4.715820 -2.615707

C 0 0.116623 -5.814056 -1.571564

C 0 0.505768 -3.074707 -0.821060

C 0 5.030472 -2.710098 3.986710

C 0 -1.411545 -5.864426 -1.251740

H 0 -0.254562 -4.401566 1.888153

H 0 -0.228973 -2.817993 2.668121

H 0 0.234604 -4.255432 3.580688

H 0 2.355621 -3.214050 4.226425

H 0 5.470764 -2.856033 1.294067

H 0 5.070602 -3.153641 -0.979980

H 0 3.458817 -2.704910 -1.551376

H 0 3.891174 -4.407242 -1.362793

H 0 0.764313 -5.892578 0.533447

H 0 2.082108 -5.884400 -0.644119

H 0 -1.965958 -3.516922 -2.998642

H 0 -1.926514 -2.389282 -1.626282

H 0 -1.932205 -4.155273 0.011688

H 0 -3.197052 -4.623245 -1.118896

H 0 1.405151 -4.601486 -2.876419

H 0 -0.222443 -4.867264 -3.532898

H 0 0.405807 -6.799133 -1.955963

H 0 4.540573 -2.316544 4.882263

H 0 5.780914 -1.982681 3.660522

H 0 5.568588 -3.620728 4.281265

H 0 -1.889023 -6.359970 -2.108292

H 0 -1.590576 -6.512741 -0.385880

C 0 0.846603 0.795486 1.112411

C 0 -0.495148 0.900513 0.830750

C 0 -1.539536 0.506210 1.847290

C 0 -2.865250 1.210007 1.753800

O 0 -1.287918 -0.349000 2.715912

H 0 1.588409 1.245728 0.460251

H 0 1.184402 0.410049 2.070862

H 0 -0.827957 1.448761 -0.048111

H 0 -3.546249 0.825729 2.514008

H 0 -2.737823 2.291290 1.895523

H 0 -3.301422 1.071259 0.756516

C 0 -1.769531 0.673828 -2.804000

C 0 -2.351608 1.875168 -2.377609

C 0 -1.577545 3.019150 -2.168427

C 0 -0.173737 3.002274 -2.397766

C 0 0.405960 1.775787 -2.823318

C 0 -0.385056 0.641357 -3.024033

N 0 0.597885 4.140640 -2.218400

C 0 2.025742 4.119171 -2.540451

C 0 -0.036072 5.409973 -1.853394

H 0 -2.383240 -0.199188 -2.997971

H 0 -3.425797 1.934540 -2.227097

H 0 -2.068832 3.933945 -1.862991

H 0 1.466507 1.717560 -3.032608

H 0 0.086166 -0.272217 -3.375031

H 0 2.206573 3.876129 -3.596771

H 0 2.452271 5.102600 -2.341797

H 0 2.568649 3.387726 -1.925720

H 0 -0.757095 5.743134 -2.612640

H 0 -0.561264 5.336685 -0.892029

H 0 0.731583 6.177948 -1.755844

TS1_F_

Au 0 -1.810287 0.052094 -0.870956

C 0 -3.809021 -0.782669 2.197617

C 0 -5.175842 -2.488449 0.950700

C 0 -6.173492 -2.049774 -1.196335

C 0 -5.931595 -2.940353 -0.138092

C 0 -4.944412 -0.313339 -0.058350

C 0 -5.967377 0.191071 -2.343292

C 0 -4.667908 -1.180786 1.016876

C 0 -5.694962 -0.734406 -1.177811

N 0 -3.077530 2.794861 -0.609894

N 0 -4.498215 1.073334 -0.014648

C 0 -5.426453 2.008469 0.721924

C 0 -1.984802 3.395004 0.200119

C 0 -2.476318 3.394791 1.669739

C 0 -4.278488 3.665390 -0.639450

C 0 -4.948029 3.476791 0.725983

C 0 -3.277298 1.439026 -0.449066

C 0 -6.495483 -4.343124 -0.165024

C 0 -3.934341 3.917938 1.828033

C 0 -0.352692 -1.473740 -1.390915

C 0 0.745834 -1.729568 -0.462463

C 0 0.402939 -2.565384 0.749100

C 0 1.158066 -3.864670 0.906815

O 0 -0.474442 -2.205811 1.553284

H 0 -3.830521 0.292648 2.394409

H 0 -2.761566 -1.062790 2.024689

H 0 -4.140060 -1.298080 3.104675

H 0 -4.969100 -3.167435 1.773071

H 0 -6.749222 -2.385452 -2.054031

H 0 -6.564743 -0.317184 -3.104675

H 0 -5.037781 0.527756 -2.817703

H 0 -6.520111 1.088989 -2.038879

H 0 -5.533768 1.609421 1.736100

H 0 -6.404892 1.942215 0.235779

H 0 -1.791626 4.403198 -0.177597

H 0 -1.079361 2.794022 0.083618

H 0 -2.412125 2.362030 2.038025

H 0 -1.807327 3.996994 2.296036

H 0 -4.923065 3.355469 -1.466919

H 0 -3.952454 4.691910 -0.819244

H 0 -5.840164 4.106277 0.828110

H 0 -5.925842 -5.016678 0.482529

H 0 -6.488861 -4.758728 -1.178162

H 0 -7.536667 -4.356018 0.183853

H 0 -3.915802 5.016678 1.808334

H 0 -4.312561 3.636108 2.818161

H 0 -0.016098 -1.280884 -2.411774

H 0 -1.087463 -2.292236 -1.362045

H 0 1.725098 -1.948563 -0.887756

H 0 0.814255 -4.385849 1.801743

H 0 1.004761 -4.507996 0.031143

H 0 2.236603 -3.676682 0.981476

C 0 1.426254 -0.035828 0.468811

C 0 2.463928 -0.454254 1.373062

C 0 3.795242 -0.304337 1.068451

C 0 4.201340 0.366226 -0.136688

C 0 3.173203 0.892685 -0.989960

C 0 1.845718 0.721771 -0.678131

N 0 5.516296 0.504776 -0.452652

C 0 5.927826 1.236603 -1.666000

C 0 6.567490 -0.049515 0.421921

H 0 0.437332 0.132141 0.881256

H 0 2.181107 -0.918365 2.312729

H 0 4.542633 -0.660904 1.764175

H 0 3.441208 1.431564 -1.888367

H 0 1.082000 1.113144 -1.342896

H 0 5.592560 2.278915 -1.632568

H 0 7.014389 1.229782 -1.732559

H 0 5.525467 0.762512 -2.567856

H 0 6.579636 0.450043 1.397263

H 0 6.423798 -1.124115 0.574921

H 0 7.536667 0.099503 -0.051041

Int1_F_

Au 0 2.367371 -0.875809 -1.450073

C 0 3.717987 0.631700 1.942245

C 0 4.891373 2.472717 0.696762

C 0 6.222763 2.083145 -1.270035

C 0 5.690536 2.983643 -0.332077

C 0 5.195419 0.224274 -0.120148

C 0 6.573074 -0.229614 -2.220718

C 0 4.626694 1.098236 0.825394

C 0 5.992706 0.705566 -1.183319

N 0 4.124557 -3.237289 -0.647644

N 0 5.020874 -1.214981 0.005341

C 0 5.945038 -1.871445 0.999588

C 0 3.031555 -4.010529 -0.006760

C 0 3.193298 -3.814545 1.522247

C 0 5.454285 -3.825989 -0.356720

C 0 5.774765 -3.404449 1.080460

C 0 3.999146 -1.859848 -0.599670

C 0 5.992554 4.462631 -0.425797

C 0 4.662506 -3.980316 2.013153

C 0 0.802277 0.145767 -2.431641

C 0 -0.115738 1.116776 -1.619217

C 0 -1.241165 0.386703 -0.808487

C 0 -0.771561 -0.411606 0.352539

C 0 -1.456039 -0.500275 1.527618

C 0 -2.643585 0.281067 1.749786

C 0 -3.057358 1.203918 0.716629

C 0 -2.393021 1.264816 -0.464188

N 0 -3.348709 0.172012 2.892212

C 0 -2.898468 -0.720078 3.982574

C 0 -4.607559 0.925873 3.088806

C 0 0.716309 2.008804 -0.705215

C 0 1.622696 3.021835 -1.359436

O 0 0.672287 1.877594 0.533798

H 0 3.565369 -0.449295 1.928650

H 0 2.733383 1.103500 1.842438

H 0 4.122253 0.910431 2.923294

H 0 4.455353 3.154984 1.421600

H 0 6.833832 2.461456 -2.084686

H 0 7.192123 0.322937 -2.932480

H 0 5.784241 -0.739517 -2.787048

H 0 7.202255 -1.007080 -1.769638

H 0 5.764328 -1.388702 1.966156

H 0 6.970947 -1.634583 0.698334

H 0 3.125916 -5.058334 -0.306808

H 0 2.069931 -3.627808 -0.358459

H 0 2.837402 -2.803223 1.762115

H 0 2.550873 -4.516068 2.068802

H 0 6.181122 -3.438248 -1.076141

H 0 5.386017 -4.908752 -0.482407

H 0 6.732452 -3.817093 1.420578

H 0 5.257233 5.058334 0.123962

H 0 5.999725 4.805954 -1.465942

H 0 6.980270 4.691132 -0.003784

H 0 4.875275 -5.053543 2.118011

H 0 4.757935 -3.549911 3.017685

H 0 0.152298 -0.573914 -2.949295

H 0 1.293839 0.732666 -3.221451

H 0 -0.638504 1.774612 -2.334839

H 0 -1.651657 -0.351364 -1.547989

H 0 0.134872 -0.991455 0.207840

H 0 -1.080917 -1.140457 2.314178

H 0 -3.909393 1.846970 0.883850

H 0 -2.732193 1.959503 -1.227188

H 0 -3.585144 -0.626297 4.821625

H 0 -2.889084 -1.764725 3.655029

H 0 -1.897308 -0.441452 4.324356

H 0 -4.417191 2.001373 3.163452

H 0 -5.297638 0.734253 2.261536

H 0 -5.075195 0.593552 4.013794

H 0 1.994598 3.725403 -0.613113

H 0 2.480545 2.505737 -1.807785

H 0 1.107040 3.562119 -2.160812

C 0 -6.336227 -0.571777 -0.438644

C 0 -6.021255 0.141418 -1.600952

C 0 -4.931915 -0.223343 -2.395477

C 0 -4.114090 -1.325378 -2.043152

C 0 -4.453583 -2.053421 -0.877090

C 0 -5.544739 -1.675232 -0.092682

N 0 -2.984802 -1.668015 -2.810745

C 0 -2.844772 -1.099152 -4.161285

C 0 -2.332809 -2.964600 -2.571945

H 0 -7.202271 -0.302811 0.156937

H 0 -6.637787 0.981094 -1.906830

H 0 -4.736740 0.338669 -3.299728

H 0 -3.880768 -2.924667 -0.587326

H 0 -5.792969 -2.267273 0.783493

H 0 -2.786118 -0.007034 -4.121353

H 0 -1.915970 -1.462341 -4.602692

H 0 -3.679596 -1.376068 -4.821609

H 0 -1.959976 -3.028229 -1.544098

H 0 -3.008850 -3.814545 -2.747360

H 0 -1.475586 -3.056992 -3.239944

TS2_F_

Au 0 -0.449203 -1.009949 -1.094604

C 0 -0.740829 -2.411087 -4.757843

C 0 -1.498764 -0.150574 -5.582993

C 0 -3.237656 1.109360 -4.495209

C 0 -2.293716 1.002900 -5.526779

C 0 -2.602005 -1.056305 -3.639908

C 0 -4.424652 0.254990 -2.433487

C 0 -1.634888 -1.194794 -4.656616

C 0 -3.409058 0.095383 -3.542938

N 0 -2.611237 -3.064758 -0.577225

N 0 -2.812622 -2.129013 -2.679886

C 0 -3.675278 -3.262115 -3.174835

C 0 -1.645386 -4.103714 -0.122925

C 0 -1.451416 -5.067047 -1.320892

C 0 -3.962921 -3.634842 -0.792191

C 0 -3.881546 -4.378891 -2.128357

C 0 -2.131546 -2.165741 -1.513229

C 0 -2.151718 2.093048 -6.565186

C 0 -2.788406 -5.486969 -2.000687

C 0 1.286194 0.115723 -0.656250

C 0 2.188385 -0.378387 0.521072

C 0 1.711075 0.026459 1.940292

C 0 0.305618 -0.076691 2.273621

C 0 -0.145400 -0.291611 3.555817

C 0 0.769165 -0.532074 4.627258

C 0 2.168700 -0.559738 4.313339

C 0 2.600082 -0.339371 3.029587

N 0 0.331085 -0.745209 5.900192

C 0 -1.109222 -0.739075 6.208618

C 0 1.280304 -1.062393 6.981430

C 0 2.339035 -1.906067 0.421555

C 0 3.116974 -2.437592 -0.762573

O 0 1.834549 -2.671112 1.261108

H 0 -1.278503 -3.292007 -5.132889

H 0 -0.310211 -2.673737 -3.785843

H 0 0.083633 -2.221054 -5.450760

H 0 -0.750366 -0.242508 -6.365112

H 0 -3.855164 2.000580 -4.428482

H 0 -4.973694 1.193878 -2.546585

H 0 -3.943192 0.261246 -1.448074

H 0 -5.160187 -0.559097 -2.427811

H 0 -3.208694 -3.644821 -4.087845

H 0 -4.646744 -2.838715 -3.452286

H 0 -2.069733 -4.605743 0.751770

H 0 -0.703629 -3.631180 0.168060

H 0 -0.810181 -4.556778 -2.052048

H 0 -0.911500 -5.966766 -1.002426

H 0 -4.694794 -2.821747 -0.814636

H 0 -4.196838 -4.289764 0.049942

H 0 -4.827530 -4.877213 -2.373749

H 0 -2.440063 3.069839 -6.163849

H 0 -2.792892 1.895737 -7.434738

H 0 -1.122482 2.166245 -6.931381

H 0 -3.240784 -6.296219 -1.409958

H 0 -2.572937 -5.913742 -2.988068

H 0 0.995956 1.157639 -0.467041

H 0 1.893341 0.135574 -1.571533

H 0 3.203461 0.041534 0.395462

H 0 -0.416580 0.045486 1.473984

H 0 -1.210938 -0.318344 3.738708

H 0 2.891251 -0.800003 5.081696

H 0 3.663940 -0.416748 2.815445

H 0 -1.242157 -0.840515 7.284912

H 0 -1.572144 0.202316 5.894318

H 0 -1.629059 -1.569443 5.716095

H 0 1.811935 -2.000641 6.785370

H 0 2.015900 -0.260483 7.109589

H 0 0.730682 -1.170197 7.915421

H 0 2.591980 -2.201706 -1.695313

H 0 4.108826 -1.972687 -0.819885

H 0 3.225693 -3.520111 -0.675674

H 0 1.874161 1.370392 1.911255

C 0 -1.644547 4.550125 3.012115

C 0 -0.544357 4.723450 3.856277

C 0 0.697830 4.164352 3.531921

C 0 0.842834 3.420929 2.351791

C 0 -0.263992 3.241196 1.504913

C 0 -1.497910 3.806747 1.834976

N 0 2.117615 2.798813 1.975662

C 0 3.197647 2.891678 2.998520

C 0 2.636139 3.266235 0.645004

H 0 -2.602463 4.990982 3.265091

H 0 -0.642746 5.303818 4.767502

H 0 1.534317 4.327362 4.198990

H 0 -0.171127 2.659271 0.595337

H 0 -2.343445 3.663849 1.170654

H 0 2.849228 2.477951 3.944443

H 0 4.048859 2.303619 2.649857

H 0 3.522125 3.928528 3.141144

H 0 1.865311 3.168823 -0.115585

H 0 2.948425 4.314682 0.712600

H 0 3.490631 2.647125 0.364578

Int2_F_

Au 0 1.139069 -0.267227 -0.732193

C 0 4.097855 0.485504 1.621078

C 0 5.560760 1.047562 -0.352005

C 0 5.691681 -0.340683 -2.314651

C 0 6.083908 0.820831 -1.631851

C 0 4.316315 -1.012680 -0.449844

C 0 4.400589 -2.502655 -2.518921

C 0 4.673035 0.150269 0.262466

C 0 4.814011 -1.271027 -1.743866

N 0 1.414841 -3.061996 0.490768

N 0 3.452072 -2.009583 0.169678

C 0 4.171570 -2.973328 1.077332

C 0 0.471512 -2.809692 1.613235

C 0 1.329300 -2.523392 2.870789

C 0 2.182053 -4.315628 0.699219

C 0 3.233704 -3.987854 1.763535

C 0 2.111176 -1.939850 0.052704

C 0 7.066452 1.788010 -2.253250

C 0 2.491608 -3.540665 3.061493

C 0 0.258011 1.457916 -1.564850

C 0 -0.404526 2.494171 -0.581543

C 0 -1.689468 1.981491 0.053574

C 0 -1.722748 1.072388 1.129929

C 0 -2.926773 0.639664 1.690353

C 0 -4.176956 1.094437 1.196838

C 0 -4.148621 1.979538 0.088043

C 0 -2.926895 2.415649 -0.447647

N 0 -5.392090 0.683151 1.767746

C 0 -5.365128 -0.082336 3.022369

C 0 -6.606461 1.463242 1.480911

C 0 0.653397 2.966568 0.420715

C 0 1.386581 4.241989 0.072922

O 0 0.935867 2.324127 1.449142

H 0 4.022964 -0.386765 2.278275

H 0 3.089645 0.910431 1.531982

H 0 4.722244 1.227646 2.127121

H 0 5.845749 1.946945 0.186478

H 0 6.078293 -0.529144 -3.312256

H 0 4.911224 -2.536728 -3.485077

H 0 3.320511 -2.510971 -2.709106

H 0 4.643326 -3.430100 -1.985000

H 0 4.723068 -2.370956 1.806183

H 0 4.908371 -3.509644 0.469482

H 0 -0.161469 -3.695053 1.734894

H 0 -0.156097 -1.949829 1.363159

H 0 1.735519 -1.509125 2.767868

H 0 0.700897 -2.521988 3.769897

H 0 2.633820 -4.617905 -0.250366

H 0 1.484665 -5.096008 1.013474

H 0 3.839966 -4.866302 2.016754

H 0 6.965607 2.790955 -1.827042

H 0 6.924255 1.863251 -3.336472

H 0 8.101685 1.462875 -2.083542

H 0 2.085953 -4.455811 3.516205

H 0 3.211838 -3.134506 3.782227

H 0 -0.499725 1.196930 -2.321045

H 0 1.057114 1.976364 -2.118118

H 0 -0.683121 3.377609 -1.176987

H 0 -0.785461 0.745026 1.560074

H 0 -2.888153 -0.018433 2.549072

H 0 -5.067368 2.393860 -0.308655

H 0 -2.947388 3.137863 -1.260345

H 0 -6.387192 -0.347000 3.297104

H 0 -4.802856 -1.013550 2.897141

H 0 -4.916763 0.482117 3.853333

H 0 -6.492477 2.524643 1.743912

H 0 -6.877335 1.400452 0.420502

H 0 -7.436066 1.052231 2.057632

H 0 0.697784 5.096008 0.116638

H 0 2.206573 4.407684 0.773697

H 0 1.777557 4.197067 -0.950912

C 0 -7.202042 -3.167938 -1.965668

C 0 -7.002365 -2.283020 -0.901138

C 0 -5.844086 -1.497421 -0.838623

C 0 -4.900787 -1.615800 -1.860794

C 0 -5.075974 -2.500443 -2.928070

C 0 -6.238220 -3.277100 -2.975739

N 0 -3.655258 -0.787003 -1.778305

C 0 -3.380035 0.003647 -3.046951

C 0 -2.443787 -1.623459 -1.376541

H 0 -8.101685 -3.771423 -2.009018

H 0 -7.744659 -2.200073 -0.115311

H 0 -5.687698 -0.821762 0.000595

H 0 -4.335083 -2.596268 -3.714005

H 0 -6.387360 -3.964432 -3.800613

H 0 -3.801559 -0.079559 -1.032059

H 0 -4.270584 0.580307 -3.294952

H 0 -2.535355 0.663712 -2.854202

H 0 -3.139053 -0.687042 -3.853317

H 0 -2.670776 -2.108658 -0.428131

H 0 -2.271881 -2.370544 -2.151459

H 0 -1.572891 -0.972687 -1.270386

TS3_F_

Au 0 1.804900 -0.749908 -0.280594

C 0 5.184570 -0.508316 1.522903

C 0 5.559265 1.823349 0.634750

C 0 5.305908 2.383300 -1.693283

C 0 5.615555 2.784348 -0.385010

C 0 4.918137 0.120682 -0.946900

C 0 4.639800 0.666641 -3.425003

C 0 5.220596 0.485413 0.381882

C 0 4.959320 1.059402 -1.998779

N 0 3.123291 -2.957260 -1.799942

N 0 4.598450 -1.262177 -1.268326

C 0 5.796738 -2.163391 -1.456787

C 0 2.592651 -4.035934 -0.915558

C 0 3.754776 -4.437332 0.026947

C 0 4.173600 -3.466309 -2.715607

C 0 5.428452 -3.611633 -1.849258

C 0 3.333725 -1.729507 -1.215469

C 0 6.028580 4.207947 -0.086838

C 0 5.113083 -4.628342 -0.707916

C 0 0.179016 0.239944 1.014816

C 0 -0.847244 -0.806137 1.540070

C 0 -1.955429 -1.217926 0.561996

C 0 -1.679993 -1.895432 -0.638443

C 0 -2.696396 -2.288223 -1.509308

C 0 -4.061005 -2.032364 -1.205276

C 0 -4.337082 -1.358566 0.013855

C 0 -3.304840 -0.971542 0.867004

N 0 -5.077393 -2.425247 -2.059357

C 0 -4.764572 -3.137604 -3.299240

C 0 -6.475098 -2.165161 -1.708527

C 0 -0.209839 -2.087524 2.090836

C 0 -0.999374 -2.857498 3.120773

O 0 0.908722 -2.492523 1.710663

H 0 6.033539 -1.203781 1.489410

H 0 4.267471 -1.106949 1.517502

H 0 5.237823 0.012466 2.482819

H 0 5.788895 2.117706 1.654770

H 0 5.344620 3.113312 -2.496841

H 0 4.716980 1.531998 -4.088700

H 0 3.626617 0.258057 -3.520523

H 0 5.329956 -0.097580 -3.803741

H 0 6.368088 -2.129547 -0.524002

H 0 6.414642 -1.715408 -2.241440

H 0 2.261215 -4.861633 -1.551590

H 0 1.744385 -3.660400 -0.340485

H 0 3.846756 -3.652023 0.788528

H 0 3.500931 -5.359558 0.562393

H 0 4.311493 -2.753250 -3.533783

H 0 3.829987 -4.414276 -3.134094

H 0 6.271378 -4.012222 -2.425140

H 0 5.593460 4.911758 -0.803391

H 0 7.119476 4.320068 -0.142900

H 0 5.723480 4.513062 0.919296

H 0 5.124130 -5.623932 -1.173355

H 0 5.926041 -4.626205 0.028214

H 0 -0.318039 1.219345 1.107132

H 0 1.004120 0.339172 1.740112

H 0 -1.361694 -0.348129 2.398682

H 0 -0.651200 -2.125381 -0.903244

H 0 -2.431000 -2.814377 -2.417389

H 0 -5.358353 -1.144608 0.300827

H 0 -3.557602 -0.455780 1.789749

H 0 -4.124191 -2.538223 -3.960815

H 0 -4.257690 -4.092972 -3.105408

H 0 -5.691116 -3.350403 -3.832916

H 0 -6.670609 -1.090271 -1.594269

H 0 -6.761688 -2.666428 -0.774017

H 0 -7.119476 -2.540604 -2.503693

H 0 -0.936539 -2.340881 4.088700

H 0 -2.057602 -2.915024 2.849411

H 0 -0.588364 -3.861725 3.238174

H 0 0.178894 0.747955 -0.357864

C 0 -3.560150 3.953079 -0.365402

C 0 -3.654770 2.596497 -0.696075

C 0 -2.514481 1.866333 -1.038910

C 0 -1.255783 2.490707 -1.057434

C 0 -1.158997 3.850861 -0.722580

C 0 -2.308594 4.572815 -0.377884

N 0 -0.067688 1.691345 -1.398315

C 0 -0.223740 0.912384 -2.671036

C 0 1.208954 2.466248 -1.426071

H 0 -4.448120 4.516998 -0.101837

H 0 -4.616531 2.095413 -0.683975

H 0 -2.615372 0.814651 -1.271408

H 0 -0.208817 4.367340 -0.733582

H 0 -2.217270 5.623932 -0.125824

H 0 0.659485 0.281418 -2.793762

H 0 -1.100555 0.270767 -2.612564

H 0 -0.318817 1.592880 -3.524170

H 0 1.182419 3.243271 -2.197525

H 0 1.387848 2.921478 -0.450638

H 0 2.025436 1.773804 -1.632645

Pro_F_

Au 0 1.853714 -0.887329 0.050735

C 0 5.658400 -1.395676 1.507645

C 0 5.730591 1.119247 1.585068

C 0 4.947495 2.501175 -0.222641

C 0 5.510941 2.400879 1.054230

C 0 4.861984 0.098800 -0.422577

C 0 4.013397 1.575989 -2.357269

C 0 5.417038 -0.043335 0.871475

C 0 4.619553 1.368210 -0.986160

N 0 3.024475 -2.569458 -2.145706

N 0 4.540573 -1.098770 -1.190857

C 0 5.712600 -1.791870 -1.838943

C 0 2.611969 -3.902618 -1.627167

C 0 3.876068 -4.533813 -0.990860

C 0 3.971451 -2.690857 -3.283218

C 0 5.317993 -3.047806 -2.646591

C 0 3.297531 -1.608643 -1.199188

C 0 5.887772 3.637146 1.838500

C 0 5.147583 -4.398407 -1.880753

C 0 -0.347748 1.994537 -2.829285

C 0 -0.759735 2.555008 -1.446900

C 0 -0.750305 1.450424 -0.382828

C 0 0.287369 1.263000 0.521729

C 0 0.323029 0.136917 1.417953

C 0 -0.813293 -0.754318 1.482910

C 0 -1.863129 -0.544113 0.540222

C 0 -1.823639 0.522400 -0.341675

N 0 -0.883896 -1.747040 2.419464

C 0 0.241135 -1.981262 3.328003

C 0 -2.025818 -2.676407 2.439240

C 0 0.139954 3.726486 -1.038284

C 0 -0.510254 4.891754 -0.334991

O 0 1.363724 3.702225 -1.266602

H 0 6.594391 -1.854935 1.162200

H 0 4.845627 -2.098007 1.296265

H 0 5.739456 -1.295639 2.593628

H 0 6.149948 1.022110 2.582458

H 0 4.743423 3.482162 -0.641296

H 0 4.707611 2.122635 -3.007126

H 0 3.103210 2.181366 -2.273972

H 0 3.757156 0.638702 -2.855209

H 0 6.423065 -2.029282 -1.042130

H 0 6.187164 -1.061920 -2.502838

H 0 2.225204 -4.489105 -2.464890

H 0 1.814300 -3.773376 -0.890381

H 0 4.046341 -4.036469 -0.026443

H 0 3.701111 -5.593689 -0.771973

H 0 4.002304 -1.741470 -3.825745

H 0 3.596024 -3.463165 -3.957291

H 0 6.095734 -3.195129 -3.405441

H 0 5.743225 3.490524 2.913986

H 0 5.295197 4.503418 1.529221

H 0 6.944809 3.892426 1.685333

H 0 5.122086 -5.184952 -2.647659

H 0 6.036377 -4.596252 -1.269684

H 0 -1.007767 1.170166 -3.117706

H 0 -0.407837 2.770569 -3.599976

H 0 0.679840 1.623825 -2.794830

H 0 -1.787994 2.935257 -1.515594

H 0 1.114365 1.963593 0.566300

H 0 0.966446 0.223022 2.289948

H 0 -2.726425 -1.195511 0.524765

H 0 -2.660782 0.649338 -1.024048

H 0 1.165787 -2.181778 2.764725

H 0 0.420059 -1.119385 3.982513

H 0 0.022507 -2.843994 3.956055

H 0 -2.973587 -2.137344 2.526276

H 0 -2.064560 -3.289948 1.530350

H 0 -1.927948 -3.336182 3.300873

H 0 0.247665 5.593689 0.016495

H 0 -1.188660 5.413925 -1.023026

H 0 -1.118240 4.545013 0.509659

C 0 -5.575943 -2.776840 1.016876

C 0 -5.948944 -1.453964 1.286530

C 0 -5.854004 -0.463654 0.306427

C 0 -5.372803 -0.770309 -0.991272

C 0 -5.004425 -2.113815 -1.256363

C 0 -5.106094 -3.092133 -0.264206

N 0 -5.260086 0.219864 -1.976135

C 0 -5.856888 1.538330 -1.739426

C 0 -4.988602 -0.173538 -3.362228

H 0 -5.677414 -3.546265 1.774811

H 0 -6.336441 -1.187988 2.265503

H 0 -6.169418 0.542557 0.549789

H 0 -4.656754 -2.402237 -2.239990

H 0 -4.833359 -4.115326 -0.506027

H 0 -5.409088 2.021194 -0.863083

H 0 -5.666458 2.175537 -2.604553

H 0 -6.944824 1.487732 -1.581039

H 0 -4.026703 -0.693253 -3.443420

H 0 -5.767349 -0.832779 -3.774918

H 0 -4.935089 0.722565 -3.982513
